# TDG is a pig-specific epigenetic regulator with insensitivity to H3K9 and H3K27 demethylation in nuclear transfer embryos

**DOI:** 10.1016/j.stemcr.2021.09.012

**Published:** 2021-10-21

**Authors:** Xin Liu, Lu Chen, Tao Wang, Jilong Zhou, Zhekun Li, Guowei Bu, Jingjing Zhang, Shuyuan Yin, Danya Wu, Chengli Dou, Tian Xu, Hainan He, Wei Zhu, Longtao Yu, Zhiting Liu, Xia Zhang, Zhen-Xia Chen, Yi-Liang Miao

**Affiliations:** 1 Institute of Stem Cell and Regenerative Biology, College of Animal Science and Veterinary Medicine, Huazhong Agricultural University, Wuhan 430070, China; 2 Key Laboratory of Agricultural Animal Genetics, Breeding and Reproduction (Huazhong Agricultural University), Ministry of Education, Wuhan 430070, China; 3 Hubei Key Laboratory of Agricultural Bioinformatics, College of Life Science and Technology, Huazhong Agricultural University, Wuhan 430070, China; 4 The Cooperative Innovation Center for Sustainable Pig Production, Wuhan 430070, China; 5 Hubei Hongshan Laboratory, Wuhan 430070, China

**Keywords:** somatic cell nuclear transfer, embryonic genome activation, ULI-NChIP-seq, *TDG*, pig, nuclear reprogramming, histone methylation, DNA methylation, donor cell, maternal-to-zygotic transition

## Abstract

Pig cloning by somatic cell nuclear transfer (SCNT) frequently undergoes incomplete epigenetic remodeling during the maternal-to-zygotic transition, which leads to a significant embryonic loss before implantation. Here, we generated the first genome-wide landscapes of histone methylation in pig SCNT embryos. Excessive H3K9me3 and H3K27me3, but not H3K4me3, were observed in the genomic regions with unfaithful embryonic genome activation and donor-cell-specific gene silencing. A combination of H3K9 demethylase KDM4A and GSK126, an inhibitor of H3K27me3 writer, were able to remove these epigenetic barriers and restore the global transcriptome in SCNT embryos. More importantly, thymine DNA glycosylase (TDG) was defined as a pig-specific epigenetic regulator for nuclear reprogramming, which was not reactivated by H3K9me3 and H3K27me3 removal. Both combined treatment and transient *TDG* overexpression promoted DNA demethylation and enhanced the blastocyst-forming rates of SCNT embryos, thus offering valuable methods to increase the cloning efficiency of genome-edited pigs for agricultural and biomedical purposes.

## Highlights

• Identification of reprogramming-resistant genes and regions in porcine SCNT embryos
• H3K9me3 and H3K27me3 are enriched in reprogramming-resistant genes and regions
• Removing H3K9me3 and H3K27me3 by *KDM4A* and GSK126 facilitates nuclear reprogramming
• Transient *TDG* overexpression promotes DNA demethylation and improves reprogramming

## Graphical abstract

**Figure:**
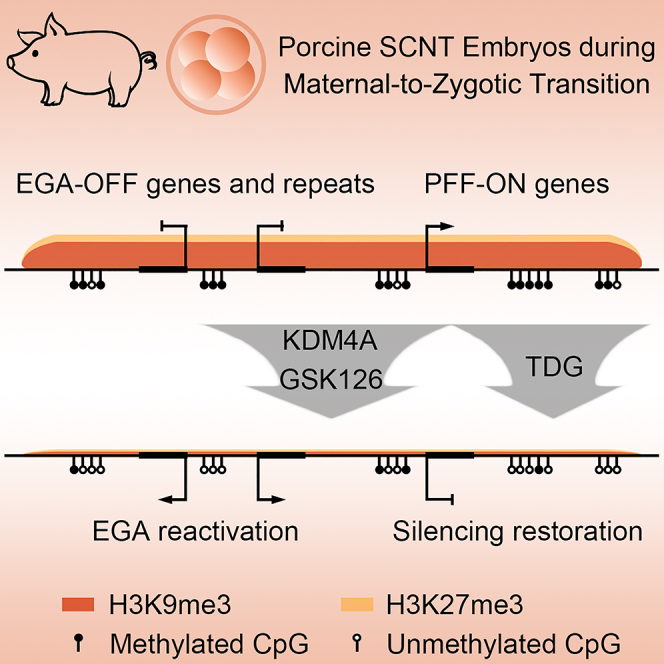

## Teaser

In this article, Miao and colleagues show that H3K9me3 and H3K27me3 are epigenetic barriers to porcine SCNT-mediated reprogramming. Removal of these modifications by *KDM4A* and GSK126 improves SCNT embryonic development, but does not reactivate *TDG* during embryonic genome activation. Meanwhile, transient *TDG* overexpression promotes DNA demethylation and also improves SCNT embryonic development in pigs.

## Introduction

Pig cloning by somatic cell nuclear transfer (SCNT) holds great promise for agriculture and biomedicine and has been widely employed to produce genome-edited pigs for breed improvement (Xu et al., 2020a; Zheng et al., 2017), human disease research (Yan et al., 2018; Zhang et al., 2018), and organ donation (Niu et al., 2017; Yue et al., 2020). For example, by deleting *CD163* and *pAPN* in donor cells, cloned pigs exhibited resistance to porcine viruses such as PRRSV, TGEV, and PDCoV (Xu et al., 2020a). In addition, PERVKO·3KO·9TG recloned pigs were created for safe and efficient xenotransplantation, which carries 3KO to eliminate xenoantigens, 9TG to enhance immunological compatibility to humans, and PERVKO to prevent viral transmission (Yue et al., 2020). However, despite tremendous efforts over the last two decades, such as modulating embryonic epigenetic modifications (Zhao et al., 2010) and knocking out *XIST* in donor cells (Ruan et al., 2018), the birth of full-term cloned pigs remains inefficient (< 3%). Notably, SCNT embryos still exhibit a developmental block during the maternal-to-zygotic transition (MZT), which is considered the consequence of incomplete epigenetic reprogramming in somatic genomes.

In mouse cloning, this incomplete reprogramming has been successfully rectified. Histone 3 lysine 9 trimethylation (H3K9me3) was the first confirmed epigenetic barrier to prevent MZT. Overexpression with H3K9 demethylases in SCNT embryos, such as lysine-specific demethylase 4D (KDM4D) and KDM4B, was able to improve embryonic genome activation (EGA) and the blastocyst rate (∼90%) (Liu et al., 2016; Matoba et al., 2014). Another repressive mark, H3K27me3, turned out to be more complicated. Injection of either *KDM6A* mRNA or *KDM6B* small interfering RNA reduced H3K27me3 enrichments in two-cell embryos and promoted blastocyst formation (Yang et al., 2018). A previous report also demonstrated that H3K4me3 impeded *Xenopus* nuclear reprogramming by maintaining the transcriptional memory of somatic cells (Hormanseder et al., 2017). However, anomalous H3K4me3 might not lead to the EGA arrest of mouse SCNT embryos (Liu et al., 2016). Before histone methylation resetting, DNA hypermethylation was identified in mouse cloning without an effective rescue approach (Tsuji et al., 2009; Yang et al., 2007). Recently, knocking down of DNA methyltransferase was determined to improve mouse cloned EGA and the blastocyst rate, and this rate was even higher (> 95%) when combined with histone demethylase overexpression (Gao et al., 2018). All these studies inspired us to investigate epigenetic barriers and defective factors during pig cloned MZT, which previously have not been comprehensively elucidated.

In the present study, we combined RNA sequencing (RNA-seq), ultra-low-input native chromatin immunoprecipitation sequencing (ULI-NChIP-seq), and whole-genome bisulfite sequencing (WGBS) to investigate histone modification and DNA methylation on the donor genome in pig SCNT embryos. More importantly, insufficient activation of thymine DNA glycosylase (TDG), a DNA demethylation regulator specifically expressed during the pig MZT, was defined as a novel barrier for nuclear reprogramming. Taken together, our study reveals a unique interplay between histone modification and DNA methylation in pig nuclear reprogramming and provides effective methods to overcome multiple epigenetic barriers in this process.

## Results

### Identification of reprogramming-resistant genes and regions in pig SCNT embryos

Large-scale analyses of the embryonic transcriptomes have reported different MZT timing between *in vivo*-fertilized (IVO) embryos (four-cell stage) and SCNT embryos (eight-cell stage) (Cao et al., 2014a; He et al., 2019). Here, we investigated the RNA-seq profiles of two- to eight-cell IVO embryos (IVO2c, IVO4c, IVO8c), four- to eight-cell SCNT embryos (SCNT4c, SCNT8c), and the donor pig fetal fibroblasts (PFF) (Table S1). Both hierarchical clustering and principal component analysis (PCA) (Figures S1A and S1B) indicated that the transcriptional patterns of SCNT8c, but not SCNT4c, were close to IVO4c, reconfirming the MZT delay in SCNT embryos. Nevertheless, 93.4% of SCNT embryos displayed a developmental block at the four-cell stage when treated with transcriptional inhibitor α-amanitin (Table S2), indicating that the newly synthesized RNA of SCNT4c is critical for the subsequent cleavage. Thus, we decided to analyze the transcriptional difference between IVO and SCNT embryos at the same four-cell stage.

By comparative analysis, 3,070 differentially expressed genes (DEGs) (fold change [FC] > 3, fragments per kilobase of exon per million mapped fragments [FPKM] > 5 in IVO4c, p < 0.05) were highly expressed in IVO4c compared with IVO2c, termed EGA-ON genes (Figure S1C and Table S3). Among these, 734 and 1,106 genes were fully (FC ≤ 2, IVO4c versus SCNT4c) and partially (2 < FC ≤ 5) activated in SCNT4c, while 1,230 genes failed to be activated (EGA-OFF; FC > 5). Gene ontology (GO) analysis suggested that SCNT4c displayed competence for maternal mRNA decay and the defects for activating genes enriched in DNA-dependent transcription (Figure S1D). Next, 4,947 downregulated DEGs were identified in IVO4c compared with PFF (FC > 3, FPKM > 5 in PFF, p < 0.05), termed PFF-OFF genes (Figure S1E and Table S3). Most genes were fully (3,678; FC ≥ 5, PFF versus SCNT4c) and partially (740; 2 ≤ FC < 5) decreased in SCNT4c, which are implicated in the Wnt signaling pathway, cell junction, and epithelium morphogenesis. Only 529 genes (PFF-ON; FC < 2) were still highly expressed during cloned MZT, and were partially involved in DNA damage response and protein ubiquitination (Figure S1F).

**Figure 1:**
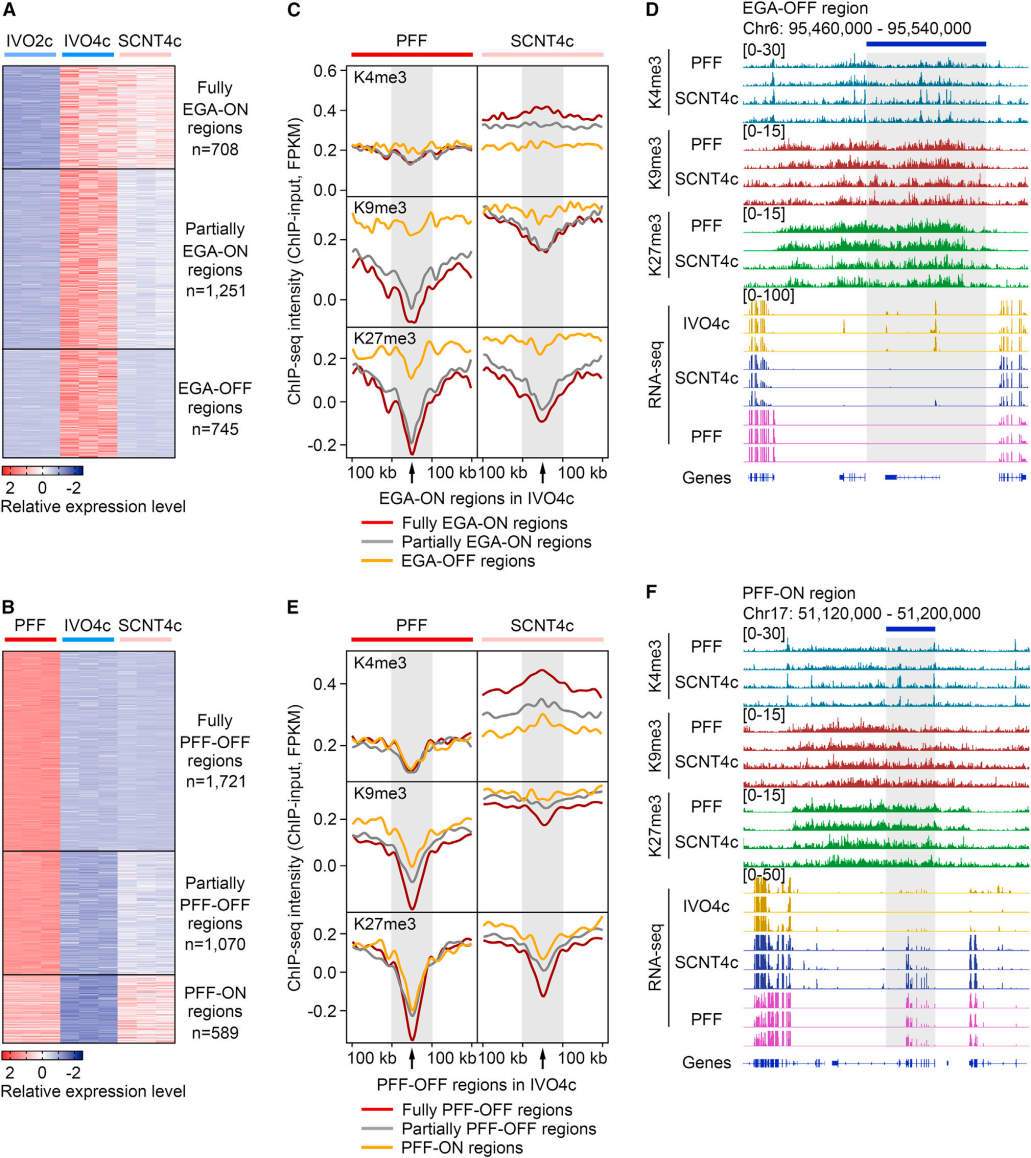
Reprogramming-resistant regions are enriched for H3K9me3 and H3K27me3 (A and B) Heatmaps showing the expression levels of EGA-ON regions (A) and PFF-OFF regions (B) in IVO4c. Each row represents the normalized RPM of a region and each column represents a replicate. EGA-ON regions, ranging from 50 to 575 kb, are classified into three groups based on the fold change (FC) in expression differences between IVO4c and SCNT4c (Fully EGA-ON, FC ≤ 2; Partially EGA-ON, 2 < FC ≤ 5; EGA-OFF, FC > 5). PFF-OFF regions, ranging from 50 to 560 kb, are classified into three groups based on their expression differences between PFF and SCNT4c (Fully PFF-OFF, FC ≥ 5; Partially PFF-OFF, 2 ≤ FC < 5; PFF-ON, FC < 2). (C and E) ChIP-seq intensity of H3K4me3, H3K9me3, and H3K27me3 are shown within EGA-ON/OFF (C) and PFF-ON/OFF (E) regions, and their 100-kb flanking regions. Read counts are normalized by input, total mapped reads, and region length. (D and F) Genome browser view of H3K4me3, H3K9me3, and H3K27me3 enrichments and transcriptional levels in the representative EGA-OFF (D) and PFF-ON (F) regions. See also Figure S2; Tables S1 and S4.

To comprehensively dissect the difference of protein-coding and non-coding transcripts, we also performed a sliding window strategy as previously reported (Chung et al., 2015; Matoba et al., 2014). Compared with IVO2c, IVO4c harbored 2,704 EGA-ON genomic regions (FC > 5, reads of exon per million mapped reads [RPM] > 10 in IVO4c, p < 0.01) (Figure 1A and Table S4). Among these, 708 regions were fully activated (FC ≤ 2, IVO4c versus SCNT4c), 1,251 regions were partially activated (2 < FC ≤ 5), and 745 regions were deficiently activated in SCNT4c (EGA-OFF; FC > 5). We also identified 3,380 PFF-OFF regions that were silenced in IVO4c compared with PFF (FC > 5, RPM > 10 in PFF, p < 0.01) (Figure 1B and Table S4). Among these, 589 regions kept their expression levels in SCNT4c as those in PFF (FC < 2, PFF versus SCNT4c), termed PFF-ON regions. Meanwhile, 1,721 and 1,070 regions were fully (FC ≥ 5) or partially (2 ≤ FC < 5) restored to the transcriptional quiescence state in SCNT4c. Here, all these EGA-OFF/PFF-ON genes and regions are termed “reprogramming-resistant genes and regions” in SCNT4c.

### EGA-OFF regions are enriched for H3K9me3 and H3K27me3 in SCNT embryos

Having ascertained reprogramming-resistant genes and regions in SCNT4c, we then asked whether their anomalous expression patterns are stabilized by epigenetic modifications from donor cells. Therefore, we performed ULI-NChIP-seq on PFF and SCNT4c with 200 cells, and generated the genome-wide maps of H3K4me3, H3K9me3 and H3K27me3 (Table S1). Strikingly, compared with the chromatin immunoprecipitation sequencing (ChIP-seq) using 10e7 PFF (Gao et al., 2019), over 70% of H3K4me3 peaks were detected in our low-input ChIP-seq data (Figures S2A and S2B). The correlation coefficients between two replicates ranged from 0.85 to 0.98 (Figure S2C), indicating the high reproducibility of this procedure.

We first assessed the histone methylation on EGA-ON/OFF regions. As shown in Figure 1C, the ChIP-seq intensities of all three marks in PFF were obviously higher on EGA-OFF regions compared with those on EGA-ON regions. Notably, EGA-OFF regions still preserved H3K9 and H3K27 hypermethylation in SCNT4c, suggesting that these two modifications are pre-existing epigenetic barriers for cloned EGA. A representative region shows the H3K9me3 and H3K27me3 depositions in PFF and SCNT4c with unfaithful gene activation in SCNT4c (Figure 1D). Simultaneously, EGA-OFF regions were deficiently enriched for H3K4me3 in SCNT4c (Figure 1C), suggesting that H3K4 hypomethylation is also responsible for EGA failure. We next determined the chromatin structures of EGA-ON/OFF regions by analyzing the distributions of protein-coding genes and repetitive sequences. As expected, EGA-OFF regions contained relatively few genes compared with EGA-ON regions, and they were enriched for repetitive sequences such as LINE and LTR (Figures S2D and S2E). These findings indicate that EGA-OFF regions are generally located in heterochromatin regions, which prevents transcriptional activation of the donor genome.

### PFF-ON genes and regions are not involved in excessive H3K4me3 in SCNT embryos

By further assessing the histone methylation on PFF-ON/OFF regions, no difference in H3K4me3 was detected among these regions in PFF, and its enrichments were even lower on PFF-ON regions in SCNT4c (Figure 1E). Moreover, except for H3K27me3 in PFF between partially PFF-OFF and PFF-ON regions, H3K9me3 and H3K27me3 levels in PFF and SCNT4c were significantly higher on PFF-ON regions than those on PFF-OFF regions (Figure 1E), an example of which is shown in Figure 1F. Since H3K9me3 and H3K27me3 are associated with gene repression (Dambacher et al., 2010), we thus wondered whether transcriptional activation still occurred in these H3K9me3/H3K27me3-marked regions. Perhaps PFF-ON regions also possess heterochromatin features to maintain the leaky expression from PFF (Saksouk et al., 2015; Vanrobays et al., 2017), because we confirmed that PFF-ON regions were also relatively gene-poor regions enriched with LINE and LTR (Figures S2D and S2E).

**Figure 2:**
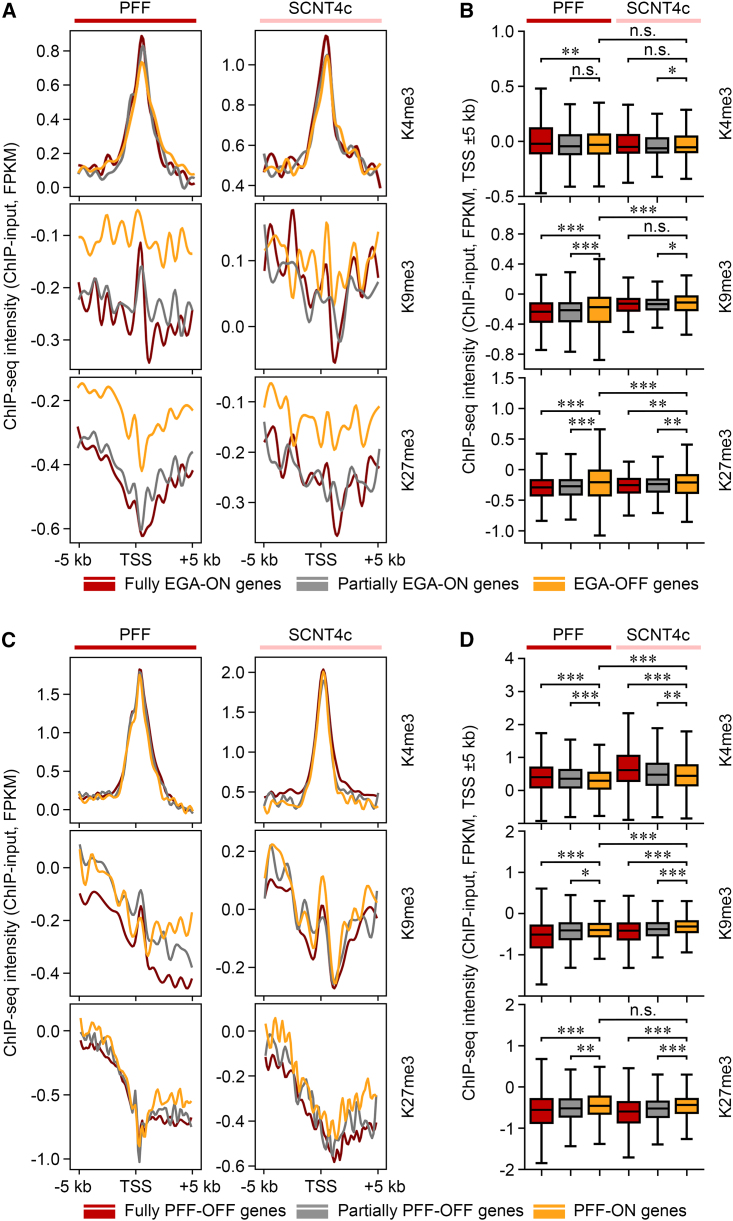
Reprogramming-resistant genes are enriched for H3K9me3 and H3K27me3 around their TSSs (A and C) ChIP-seq intensity of H3K4me3, H3K9me3, and H3K27me3 are shown around the transcription start sites (TSSs; ±5 kb) of EGA-ON/OFF (A) and PFF-ON/OFF (C) genes. Read counts are normalized by input and total mapped reads. (B and D) Box plots comparing the average intensity of H3K4me3, H3K9me3, and H3K27me3 around the TSSs (±5 kb) of EGA-ON/OFF (B) and PFF-ON/OFF (D) genes. ^∗^p < 0.05, ^∗∗^p < 0.01, ^∗∗∗^p < 0.001; n.s., not significant; two-tailed Student's t test. Reads counts are normalized by input and total mapped reads. See also Figure S1 and Table S3.

Recently, donor-cell-specific genes expressed in *Xenopus* SCNT embryos have been reported to exhibit H3K4me3 hypermethylation on their transcription start sites (TSSs) (Hormanseder et al., 2017). However, PFF-ON genes in pigs exhibited the lowest H3K4me3 enrichments around their TSSs (±5 kb) in PFF and SCNT4c when compared with PFF-OFF genes, while EGA-OFF genes only exhibited relatively low H3K4me3 enrichments in PFF when compared with fully EGA-ON genes (Figures 2A–2D). Meanwhile, high levels of H3K9me3 and H3K27me3 were observed around the TSSs of EGA-OFF/PFF-ON genes (Figures 2A–2D), consistent with the results of EGA-OFF/PFF-ON regions. Taking these data together, we propose that PFF transcriptional profiles preserved in SCNT4c are stabilized by H3K9me3 and H3K27me3, but not H3K4me3.

### *KDM4A* overexpression and GSK126 incubation improve SCNT developmental potential

To overcome aberrant H3K9 and H3K27 methylation on EGA-OFF/PFF-ON regions, we injected 1,000 ng/μL mRNA encoding the pig histone demethylases, KDM4A and KDM6A, into one-cell SCNT embryos (Figure S3A and Table S6). Global histone modifications were then determined by immunofluorescence in SCNT4c (Figures S3B and S3C). In the control group, 100% and 65.2% of embryos were positively stained with H3K9me3 and H3K27me3, respectively. These percentages were decreased to ∼5%–10% when embryos were injected with *KDM4A* and *KDM6A* individually or in combination (Figure S3C). Nonetheless, compared with the controls (16.6%), *KDM6A* injection showed no improvement on blastocyst formation (19.5%), and even impaired the beneficial effect of *KDM4A* (27.5%) when both mRNAs were injected (15.3%) (Figures S3D and S3E; Table S2). Thus, we decided to find another approach to reduce H3K27me3.

**Figure 3:**
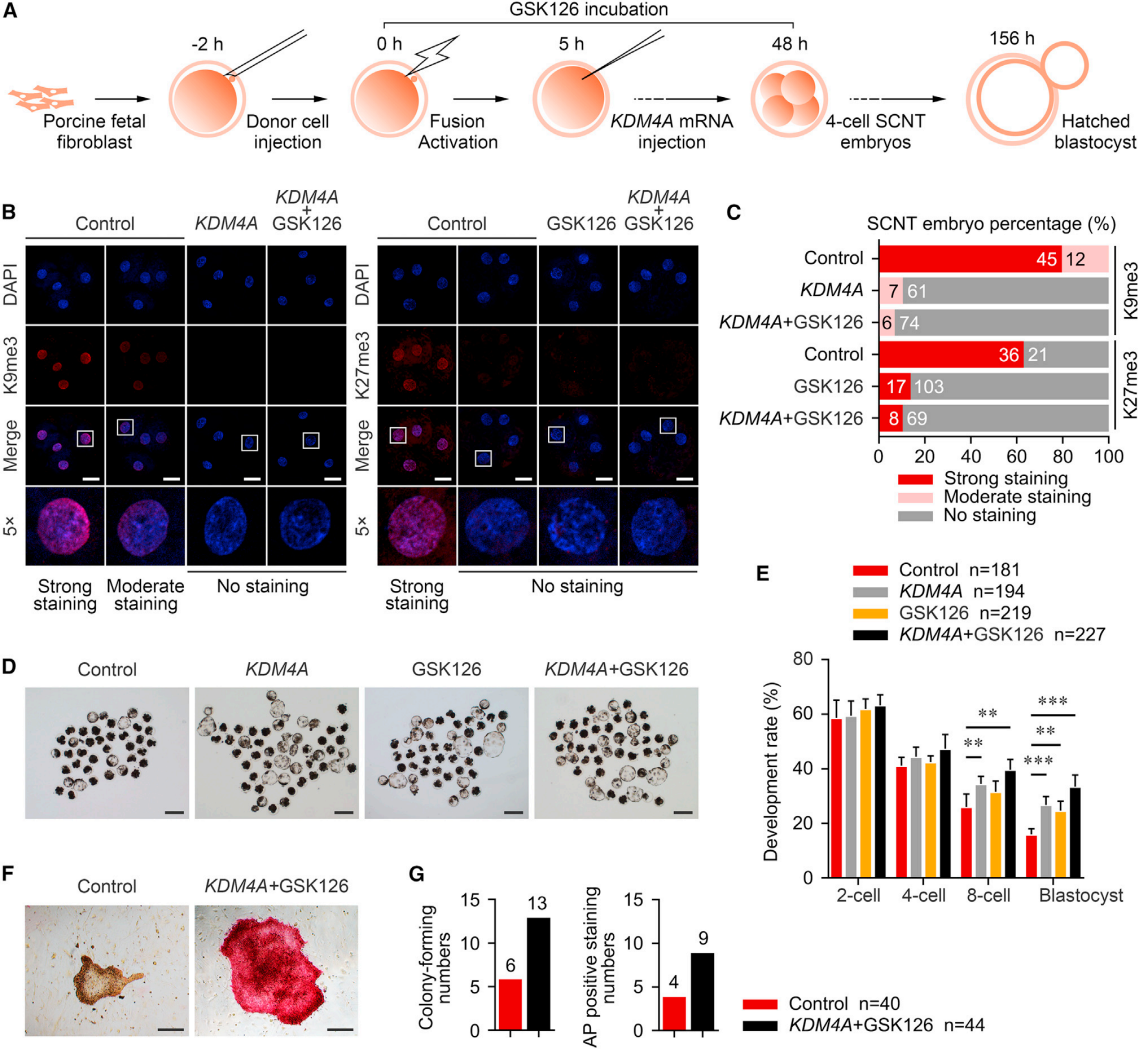
*KDM4A* + GSK126 treatment improves SCNT embryonic development (A) Experimental design of the treatment procedure. SCNT embryos were injected with *KDM4A* mRNA at 5 h after activation, or treated by GSK126 at 0–48 h. (B) Immunostaining of H3K9me3 and H3K27me3 (red) and DNA (blue) in SCNT4c derived from 1,000 ng/μL *KDM4A*-injected, 0.1 μM GSK126-treated, combined-treated, and non-treated groups. One of the nuclei in SCNT4c is magnified 5-fold. Scale bars, 50 μm. (C) Bar plot showing the percentage of SCNT4c with positive H3K9me3 and H3K27me3 staining and no staining in different groups. Numbers of the total embryos analyzed from 3 to 5 replicates are shown in the bars. (D) Representative images of different groups after culturing for 6.5 days *in vitro*. Scale bars, 200 μm. (E) Bar plot showing the development rates in different groups. Error bars represent the SD. Numbers of the total embryos analyzed from five replicates are shown in the legend. ^∗∗^p < 0.01, ^∗∗∗^p < 0.001; two-tailed Student's t test. (F) Representative images of outgrowth colonies in combined-treated group with alkaline phosphatase (AP)-positive staining and non-treated group with AP-negative staining. Scale bars, 200 μm. (G) Bar plots comparing the colony-forming and AP-positive staining numbers in combined-treated and non-treated groups. Numbers of the total blastocysts analyzed from five replicates are shown in the legend. See also Figure S3 and Table S2.

Suppression of EZH2 activity, the methyltransferase responsible for establishing H3K27me3, also contributed to nuclear reprogramming (Xie et al., 2016; Zhou et al., 2019). Hence, an EZH2-specific inhibitor, GSK126, was used during cloned MZT (Figure 3A). As shown in Figures 3B and 3C, the majority of embryos (85.8%) exhibited no H3K27me3 staining when they were treated with 0.1 μM GSK126 for 48 h. More importantly, comparable blastocyst rates were observed in the GSK126-treated (24.7%) and *KDM4A*-injected groups (26.8%), and this rate was even higher when combining these two methods (33.5%) (Figures 3D and 3E; Table S2). Blastocyst quality was then evaluated by SOX2 immunostaining and TUNEL assay. The results revealed a significant increase of total cell number and inner cell mass cell number in the experimental group, while the number of apoptotic cells showed no decline (Figures S3F and S3G). Furthermore, the experimental group also showed an increased outgrowth colony-forming number with alkaline phosphatase-positive staining (Figures 3F and 3G), suggesting its improved competence for post-implantation development.

### Combination of *KDM4A* and GSK126 restores the transcriptome of SCNT embryos

**Figure 4:**
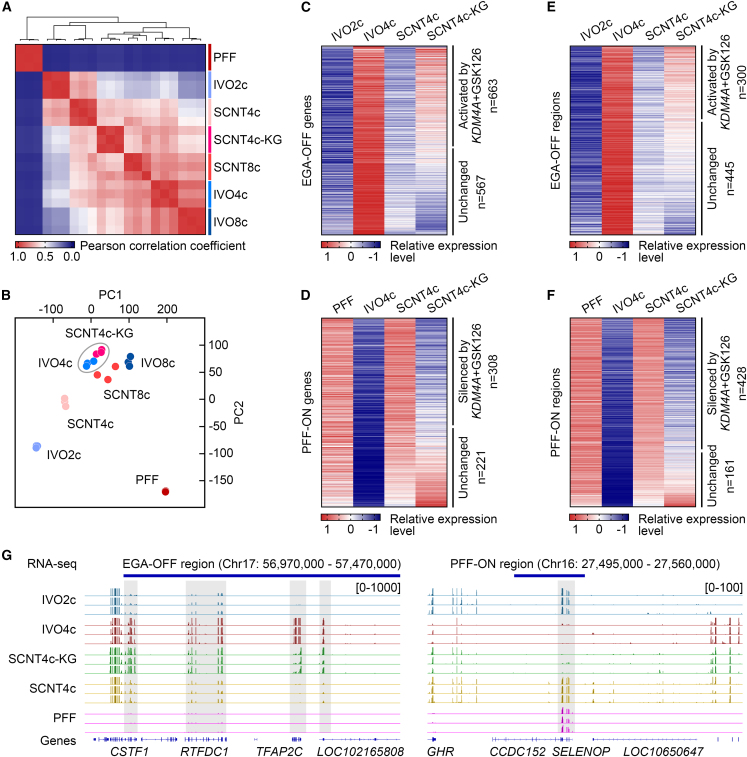
*KDM4A* + GSK126 treatment facilitates EGA initiation and somatic cell memory silencing (A) Heatmap comparing the Pearson correlation coefficients of PFF, IVO, and SCNT embryos at different stages, and SCNT4c derived from combined *KDM4A* + GSK126 treatment group (SCNT4c-KG). Hierarchical clustering is shown in the top panel. (B) Principal component analysis of expression patterns among different samples. (C and D) Heatmap showing the expression levels of EGA-OFF genes (Figure S1C) among IVO2c, IVO4c, SCNT4c, and SCNT4c-KG (C), and PFF-ON genes (Figure S1E) among PFF, IVO4c, SCNT4c, and SCNT4c-KG (D). Each row represents the normalized FPKM of a transcript. FC > 2 are EGA-OFF genes reactivated in SCNT4c-KG (SCNT4c-KG versus SCNT4c), FC < 0.5 are PFF-ON genes silenced in SCNT4c-KG. (E and F) Heatmap showing the expression levels of EGA-OFF regions (Figure 1A) among IVO2c, IVO4c, SCNT4c, and SCNT4c-KG (E), and PFF-ON regions (Figure 1B) among PFF, IVO4c, SCNT4c, and SCNT4c-KG (F). Each row represents the normalized RPM of a region. FC > 2 are EGA-OFF regions reactivated in SCNT4c-KG (SCNT4c-KG versus SCNT4c), FC < 0.5 are PFF-ON regions silenced in SCNT4c-KG. (G) Genome browser view of transcriptional levels in representative EGA-OFF/PFF-ON regions. See also Figures S4 and S5; Tables S3 and S4.

To determine the effects of *KDM4A* + GSK126 treatment, we performed RNA-seq of combined-treated SCNT4c (SCNT4c-KG). The hierarchical clustering and PCA analysis results revealed that the transcriptional patterns of SCNT4c-KG were close to IVO4c, but different from SCNT4c (Figures 4A and 4B). By profiling the data, as much as 53.9% of the 1,230 EGA-OFF genes (FC > 2, SCNT4c-KG versus SCNT4c) and 58.2% of the 529 PFF-ON genes (FC < 0.5) were restored by *KDM4A* + GSK126 treatment, and it also upregulated 40.3% of the 745 EGA-OFF regions and downregulated 72.7% of the 589 PFF-ON regions in SCNT4c (Figures 4C–4F and S4A; Tables S3 and S4). Figure 4G displays the representative genome browser view of two candidate reprogramming-resistant regions containing several restored genes in SCNT4c-KG. We also analyzed the expression of six EGA-OFF genes (Figures S5A and S5B) via qPCR, and found that *KDM4A* + GSK126 treatment successfully upregulated *KLF17*, *PNRC1*, *SUPT4H1*, *TFAP2C*, and *ZSCAN4* (Figure S5C and Table S6), partially confirming our RNA-seq results (Figure S5B).

**Figure 5:**
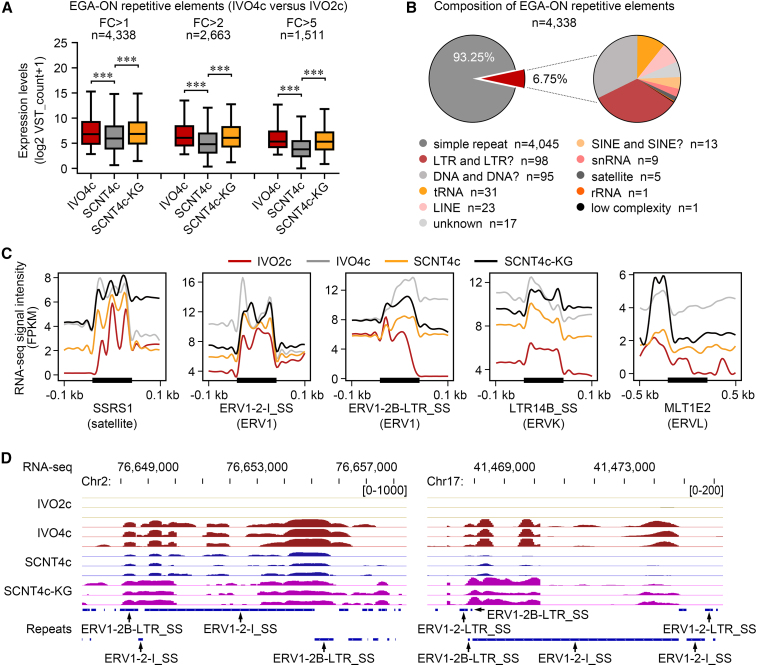
*KDM4A* + GSK126 treatment reactivates repetitive element transcription (A) Box plots comparing the normalized VST count of EGA-ON repetitive elements among IVO4c, SCNT4c, and SCNT4c-KG. Repetitive elements are shown in three groups based on their expression differences between IVO2c and IVO4c (FC > 1, 2, 5). ^∗∗∗^p < 0.001; two-tailed Student's t test. (B) The composition of 4,338 EGA-ON repetitive elements. (C) RNA read enrichments (FPKM) around candidate repetitive elements among IVO2c, IVO4c, SCNT4c, and SCNT4c-KG. (D) Genome browser view of transcriptional levels in two genomic regions containing ERV1-2-I_SS and ERV1-2B-LTR_SS. See also Figure S4 and Table S5.

In addition to protein-coding genes, repeatome also exhibits a shift during the MZT. Previous studies have identified several repetitive elements that possess EGA-ON profiles in mouse (MERVL and GSAT), human (HERVL and HSATII), and cattle (MLT1A0 and ERV1-2-I_BT) embryos (Halstead et al., 2020; Hendrickson et al., 2017). Moreover, the repression of satellite DNA and MERVL in mouse SCNT embryos was relieved by *KDM4D* overexpression (Matoba et al., 2014). In the pig, we identified 4,338 repeat sequences that were highly expressed in IVO4c compared with IVO2c (FC > 1, variance stabilizing transformation [VST] count > 5 in IVO4c) (Figures 5A and 5B; Table S5). Strikingly, these repeats were poorly activated in SCNT4c, and *KDM4A* + GSK126 treatment improved their transcriptions (Figure 5A). In detail, eight types of repetitive elements were successfully reactivated, including LTR and satellite, while LINE and DNA transposons were insensitive to the combined treatment (Figure S4B and Table S5). In particular, we defined SSRS1 (satellite), ERV1-2-I_SS (ERV1), ERV1-2B-LTR_SS (ERV1), MLT1E2 (ERVL), and LTR14B_SS (ERVK) as EGA indicators for pig IVO and SCNT embryos during the MZT (Figures 5C and 5D).

### Unfaithful activation of *TDG* results in abnormal DNA demethylation in SCNT embryos

**Figure 6:**
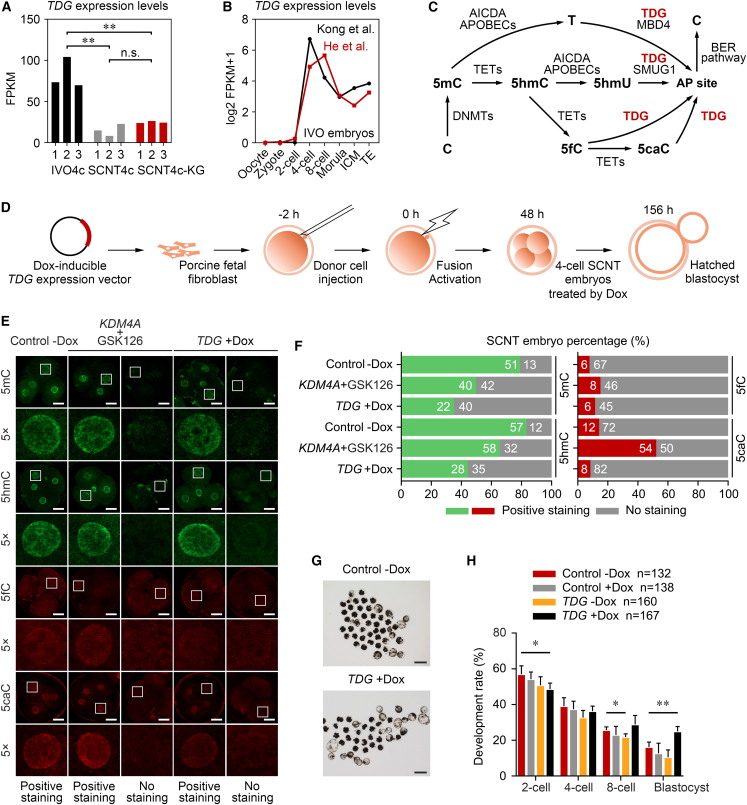
Transient *TDG* expression improves SCNT embryonic development (A) Bar plots showing the FPKM of *TDG* in the RNA-seq data of IVO4c, SCNT4c, and SCNT4c-KG. ^∗∗^p < 0.01; n.s., not significant; two-tailed Student's t test. (B) Line plots illustrating the dynamic transcriptional changes of *TDG* in pig IVO embryos (He et al., 2019; Kong et al., 2020). (C) Schematic illustration of regulators related to the DNA methylation (C→5mC) and demethylation (5mC→C) process. AP site, abasic site; BER, base excision repair. (D) Experimental design of the treatment procedure. SCNT embryos were reconstructed using PFF transfected with *TDG*-inducible expression vector, and induced to transiently express *TDG* by doxycycline (*TDG* + Dox) at 40–72 h after activation. (E) Immunostaining of 5mC (green), 5hmC (green), 5fC (red), and 5caC (red) in SCNT4c derived from *TDG* + Dox, combined-treated, and non-treated groups. One of the nuclei in SCNT4c is magnified 5-fold. Scale bars, 50 μm. (F) Bar plots showing the percentage of SCNT4c derived from different groups with positive and no staining of DNA methylation. Numbers of the total embryos analyzed from 3 to 4 replicates are shown in the bars. (G) Representative images of *TDG* + Dox and non-treated groups after culturing for 6.5 days *in vitro*. Scale bars, 200 μm. (H) Bar plot showing the development rates in the *TDG* ± Dox, Dox-treated, and non-treated groups. Error bars represent the SD. Numbers of the total embryos analyzed from four replicates are shown in the legend. ^∗^p < 0.05, ^∗∗^p < 0.01; two-tailed Student's t test. See also Figures S4–S7 and Table S2.

Among the EGA-OFF genes, we surprisingly found that the deficient *TDG* expression could not be rescued by *KDM4A* + GSK126 treatment (Figure 6A). Notably, *TDG* is specifically transcribed during pig MZT (He et al., 2019; Kong et al., 2020) and is barely detected at the MZT stage in mouse (Deng et al., 2014; Liu et al., 2016), human (Hendrickson et al., 2017; Yan et al., 2013), and cattle embryos (Graf et al., 2014; Jiang et al., 2014) (Figures 6B and S4C). TDG is a well-known enzyme for excising thymine, 5-hydroxymethyluracil (5hmU), 5-formylcytosine (5fC), and 5-carboxylcytosine (5caC), which are generated from either 5-methylcytosine (5mC) deamination by AICDA/APOBECs or 5mC hydroxylation by TET proteins (Figure 6C) (Cortellino et al., 2011; Maiti and Drohat, 2011). Accordingly, we surmise that insufficient activation of *TDG* may lead to an abnormal DNA demethylation in pig SCNT4c.

We first determined that *TDG* mRNA injection before MZT worsened SCNT blastocyst formation in a dose-dependent manner (Tables S2 and S6). A doxycycline-inducible donor cell line was then used to achieve transient *TDG* expression during cloned MZT (Figure 6D). By immunostaining, when compared with the controls (5mC, 79.7%; 5-hydroxymethylcytosine [5hmC], 82.6%), SCNT4c-KG exhibited loss of 5mC (48.8%) and 5hmC (64.4%), and these percentages (5mC, 35.5%; 5hmC, 44.4%) were much lower in SCNT4c with *TDG* overexpression (SCNT4c-TDG; Figures 6E and 6F). SCNT4c-KG also showed an obvious 5caC accumulation (51.9%), perhaps due to the upstream DNA demethylation process but still lacking *TDG* expression. By contrast, SCNT4c-TDG showed a small proportion of positive 5caC staining (8.9%) (Figures 6E and 6F). Excitingly, qPCR results revealed that transient *TDG* expression could reactivate several candidate EGA-OFF genes (Figure S5C and Table S6) and attenuate the developmental defects of SCNT embryos with an ∼10% elevation in blastocyst rate (Figures 6G and 6H; Table S2).

**Figure 7:**
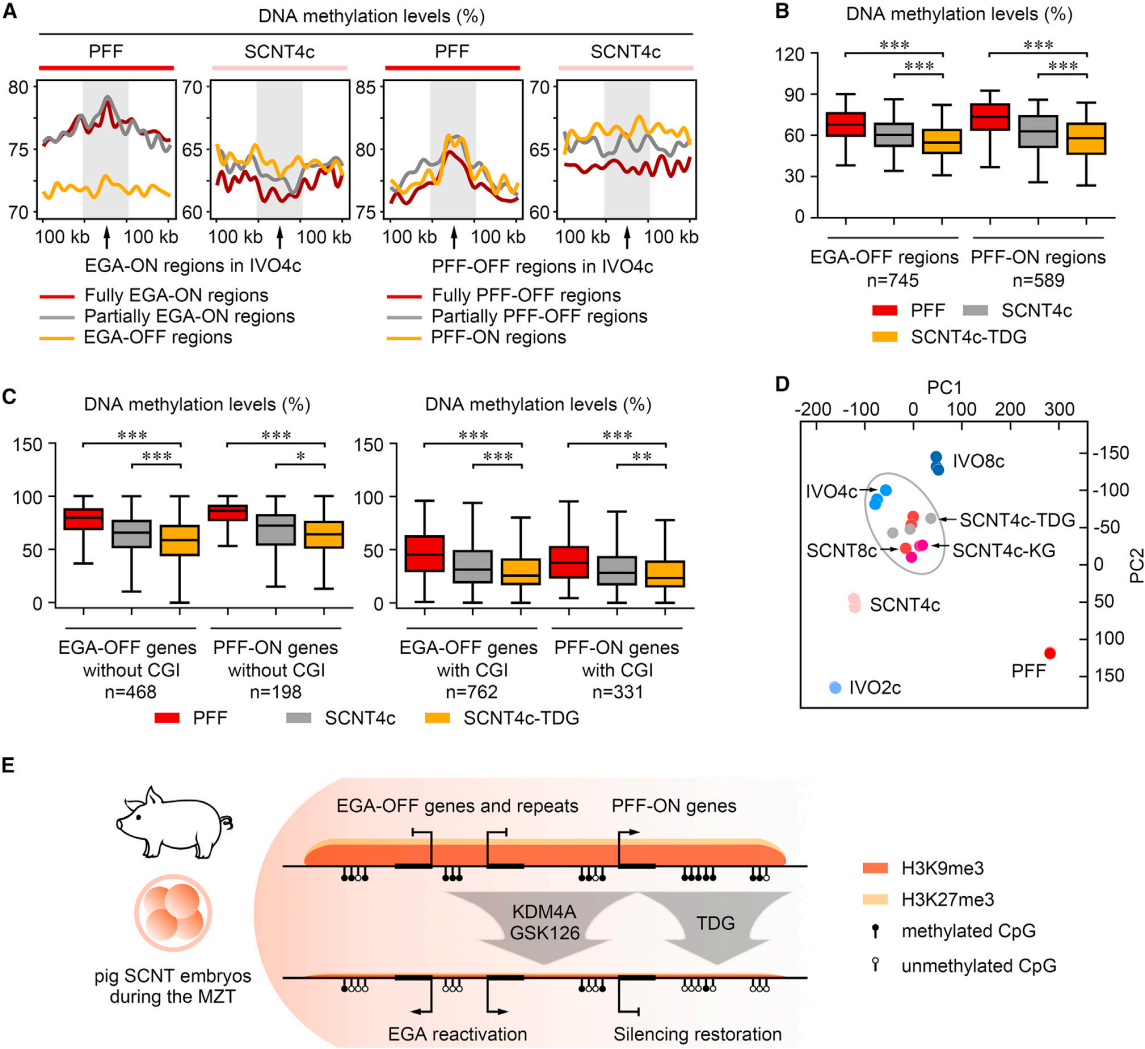
TDG regulates aberrant DNA methylation and transcription in SCNT embryos (A) DNA methylation levels of EGA-ON/OFF and PFF-ON/OFF regions with their 100-kb flanking regions in PFF and SCNT4c. These levels are calculated as the average ratios of the methylated CpG to all CpG. (B) DNA methylation levels of EGA-OFF and PFF-ON regions in PFF, SCNT4c, and SCNT4c overexpressed with *TDG* (SCNT4c-TDG). EGA-OFF regions are ranging from 50 to 575 kb and PFF-ON regions are ranging from 50 to 425 kb. These levels are calculated as the average ratios of the methylated CpG to all CpG. ^∗∗∗^p < 0.001; two-tailed Student's t test. (C) DNA methylation levels of EGA-OFF and PFF-ON genes in PFF, SCNT4c, and SCNT4c-TDG. These levels are calculated as the average ratios of the methylated CpG to all CpG. EGA-OFF and PFF-ON genes are shown in two groups: gene promoters (TSSs ±5 kb) with or without CpG island (CGI). ^∗^p < 0.05, ^∗∗^p < 0.01, ^∗∗∗^p < 0.001; two-tailed Student's t test. (D) Principal component analysis of expression patterns among different samples. (E) A schematic illustrating how multiple epigenetic barriers in pig cloning can be overcome. Abnormal H3K9me3, H3K27me3, and DNA methylation are enriched in EGA-OFF and PFF-ON regions during cloned MZT, resulting in the failure of embryonic genome activation and somatic cell memory silencing. Combining *KDM4A* overexpression and GSK126 incubation could remove H3K9me3, H3K27me3, and DNA methylation, restore the global transcriptome and repeatome, and provide a double increase for cloned blastocyst formation. Moreover, DNA demethylation and developmental improvement could also be achieved by *TDG* overexpression. See also Figures S6 and S7; Tables S3 and S4.

To precisely analyze the DNA methylation changes among PFF, SCNT4c, and SCNT4c-TDG, we performed post-bisulfite adapter tagging sequencing (PBAT-seq), an ultra-low-input WGBS method, to generate their DNA methylation maps on a genome-wide scale. First, when compared with EGA-ON/PFF-OFF regions, DNA methylation levels were higher in EGA-OFF/PFF-ON regions in SCNT4c (Figure 7A). This confirms that DNA methylation exhibits the same reprogramming-resistant feature as H3K9me3 and H3K27me3 in SCNT4c. Second, the DNA methylation levels of EGA-OFF/PFF-ON regions in SCNT4c-TDG were significantly lower than those in PFF and SCNT4c, indicating an effective DNA demethylation achieved by transient *TDG* expression (Figure 7B). DNA demethylation by TDG could also be observed at EGA-OFF/PFF-ON gene promoters, no matter whether these promoters contained a CpG island or not (Figure 7C). Third, as examples, the DNA methylation levels of satellite DNA, candidate EGA-OFF gene promoters (*TFAP2C*, *SUPT4H1*, *KLF17*), and PFF-ON gene promoters (*UBC*, *USP5*, *TRIM8*) were examined by bisulfite sequencing PCR (Table S6), and their downtrends of DNA methylation were correlated with their transcriptional restorations in SCNT4c-TDG (Figures S5C and S6A–S6C; Tables S3 and S5). Fourth, we determined the transcriptional profile of SCNT4c-TDG, which was also close to IVO4c, like SCNT4c-KG (Figure 7D). Moreover, we found that most restored genes and regions were shared in SCNT4c-KG and SCNT4c-TDG (Figures S7A–S7E; Tables S3 and S4), suggesting that the detailed gene network regulated by H3K9me3, H3K27me3, and DNA methylation are highly similar during pig cloned EGA.

## Discussion

In fertilized embryos, histone methylation and DNA methylation undergo a widespread decrease at the pre-implantation stage in mouse (Gao et al., 2018; Liu et al., 2004; Xu et al., 2020b), human (Xu et al., 2020b; Zhu et al., 2018), cattle (Liu et al., 2018; Zhou et al., 2019, 2020), and pig (Cao et al., 2014b, 2015). By contrast, SCNT embryos frequently exhibit abnormal remodeling of these modifications on the donor genome, which is believed to be the primary reason for the significant embryonic losses during cleavage. In this study, we demonstrated that H3K9me3 and H3K27me3, but not H3K4me3, were epigenetic barriers to prevent EGA initiation and somatic cell memory silencing during pig cloned MZT. Moreover, DNA methylation was reduced along with H3K9me3 and H3K27me3 removal, whereas DNA demethylation could also be achieved by overexpressing a pig-specific epigenetic regulator, TDG (Figure 7E).

H3K9me3 and H3K27me3 have been verified as conserved reprogramming barriers among different species. In pig cloning, aberrant H3K9me3 and H3K27me3 have been observed at the one- to two-cell stage via immunofluorescence, while their detrimental effects to MZT remain unknown (Ruan et al., 2018; Xie et al., 2016). By using ULI-NChIP-seq, we showed for the first time that both H3K9me3 and H3K27me3 still existed during the MZT. More precisely, they were deposited on the genomic regions with unfaithful transcriptional patterns, namely EGA-OFF and PFF-ON regions. By contrast, EGA-OFF regions in mouse and human SCNT embryos were only enriched for H3K9me3, but not H3K27me3, and the information on PFF-ON regions was not mentioned (Chung et al., 2015; Matoba et al., 2014). Additionally, most studies utilized the corresponding demethylases to overcome these barriers, such as KDM4A for H3K9me3 in mouse and human (Chung et al., 2015), and KDM6A for H3K27me3 in mouse and cattle (Yang et al., 2018; Zhou et al., 2019). Our results confirmed their demethylation functions in pig SCNT embryos, but the blastocyst rate was not elevated by KDM6A, regardless of mRNA concentrations (Table S2). Previous studies have individually utilized KDM4A and GSK126 to increase the pig SCNT blastocyst rate (Ruan et al., 2018; Xie et al., 2016). Here, we combined these two methods with a longer GSK126 treatment time (up to 48 h) to further facilitate blastocyst formation, which might be due to an obvious reduction in cloned MZT defect.

In addition to EGA failure, persistent expression of donor-cell-specific genes, namely PFF-ON genes, also derives from incomplete reprogramming. Currently, excessive H3K4me3 has been detected in cattle eight-cell SCNT embryos (Zhou et al., 2020) and on the TSSs of PFF-ON genes in *Xenopus* donor cells (Hormanseder et al., 2017). Here, we found that PFF-ON regions or the TSSs of PFF-ON genes in pig cloning were enriched for H3K9me3 and H3K27me3, but not H3K4me3. Remarkably, most PFF-ON genes and regions were silenced through H3K9me3 and H3K27me3 removal. We also noticed that PFF-ON genes in *Xenopus* and cattle SCNT embryos were involved in donor cell identity, such as endoderm- and fibroblast-specific genes (Hormanseder et al., 2017; Zhou et al., 2020). In contrast, only nine PFF-ON genes were fibroblast-specific genes when we compared our PFF-ON genes with a list of 563 marker genes for mouse fibroblasts (Table S3) (Kim et al., 2010). Therefore, although SCNT embryos harbor PFF-ON genes in many species, the composition of PFF-ON genes and how they maintain their transcriptions are likely different between pig and other species. Another difference between our work and previous studies is the effect of H3K4me3 removal for reprogramming. H3K4 demethylation has been proved to promote development in mouse, cattle, and *Xenopus* cloning (Hormanseder et al., 2017; Liu et al., 2016; Zhou et al., 2020). Accordingly, we injected *KDM5A* mRNA (Table S6) and tested its H3K4 demethylase function in four-cell embryos (Figures S7F and S7G). However, *KDM5A* overexpression could not rescue the EGA defect and the poor developmental phenotype of pig SCNT embryos (Figure S5C and Table S2).

Soon after the establishment of the SCNT technology, DNA hypermethylation was the first determined epigenetic barrier. Deficient 5mC and 5hmC demethylation has been found in pig and cattle SCNT embryos by immunostaining (Cao et al., 2014b; Zhou et al., 2020). Most studies utilized 5-aza-2-deoxycytidine (5AZA), a DNA methyltransferase inhibitor, to deplete 5mC. However, improvements could be observed in human and pig (Huan et al., 2013; Sun et al., 2012) but not in mouse and cow (Ding et al., 2008; Tsuji et al., 2009). In this study, we investigated the interplay between histone modification and DNA methylation, since *KDM4A* + GSK126 treatment decreased the numbers of embryos with 5mC- and 5hmC-positive staining. Of note, *DNMT1* and *DNMT3B* are downregulated by this treatment (Table S3), similar to the effect of 5AZA. Furthermore, TDG is likely to serve as a rate-limiting factor for DNA demethylation in pig nuclear reprogramming, because its expression not only removes 5caC but also removes the upstream 5mC and 5hmC. In this study, we also combined *KDM4A* + GSK126 treatment with transient *TDG* expression to verify whether this triple treatment could raise the efficiency of nuclear reprogramming. Unexpectedly, it showed no more improvement of cloned blastocyst formation (Table S2). We speculate that the developmental improvement achieved by TDG is mainly related to 5mC demethylation, which is shared by *KDM4A* + GSK126 treatment. Perhaps 5caC accumulation in SCNT4c-KG may not be a reprogramming barrier for MZT, thus further removing 5caC by TDG may not show a further improvement by triple treatment. Future studies also should elucidate two additional questions: (1) 5mC and 5hmC were comparably enriched in SCNT embryos when compared with PFF, while 5fC and 5caC were reduced in SCNT embryos (Figures 6F and S7H). What causes this difference? (2) AICDA and SMUG1 process EGA-specific profiles and fail to be activated in SCNT embryos (Table S3). What is the role of 5mC deamination in nuclear reprogramming?

In summary, our study advances the understanding of epigenetic remodeling in pig SCNT embryos on a genome-wide scale and provides an effective combination strategy to remove multiple barriers during the MZT. Meanwhile, we defined TDG as a novel and species-specific regulator to potentiate the developmental competence of SCNT embryos. Recently, the H3K9me3 methyltransferase inhibitor chaetocin exhibited a beneficial effect in pig cloning (Weng et al., 2020). Therefore, combinational use of chaetocin, GSK126, and 5AZA will require optimization to further improve the cloning efficiency. This work will accelerate the practical use of the SCNT technique for pig model production and contribute to the studies of human disease, xenotransplantation, and molecular breeding in agriculture.

## Experimental procedures

All experimental procedures were approved by the Animal Care Commission of Huazhong Agriculture University, Wuhan, China.

### RNA sequencing and data processing

The total RNA of PFF and 30 SCNT embryos were extracted by TRIzol (Invitrogen) or a PicoPure RNA Isolation Kit (Applied Biosystems), respectively. Double-stranded cDNA was then synthesized by a SMARTer Pico PCR cDNA Synthesis Kit (Clontech) and an Advantage 2 PCR Kit (Clontech), and purified by AMPure XP beads (Beckman Coulter). After fragmentation with a Bioruptor Sonication System (Diagenode), cDNA libraries were generated by a VAHTS Universal V6 RNA-seq Library Prep Kit (Vazyme). Paired-end 150-bp sequencing was performed on an Illumina HiSeq X Ten platform.

Illumina Casava (v1.8.2) was used for base calling. Low-quality reads were removed by Trimmomatic (v0.39). The filtered reads were mapped to the pig reference genome (Sscrofa11.1) with STAR (v2.5.3a) to obtain uniquely mapped reads. Transcripts were reconstructed by StringTie (v2.1.2) to define protein-coding novel transcripts by GffCompare (v0.10.2) and CPC2. Complete reference annotation consisted of novel and known transcripts. Gene expression was then quantified to FPKM using Cufflinks (v2.2.1). The expression levels of repetitive elements were assessed by bedtools (v2.29.2), and normalized by VST count. The RNA-seq signal intensities of repetitive elements were quantified and visualized by deeptools (v3.1.3). RepeatMasker annotation was downloaded from the UCSC Genome Browser. Pearson correlation calculation, hierarchical clustering analysis, and PCA were performed using cor, hclust, and princomp functions in R (v3.5.2), respectively. Mapping reads of genes and repeats were visualized using Integrative Genomics Viewer (IGV) software (v2.7.2).

### Identification of differentially expressed genes

FC and adjusted p values among samples were analyzed by DESeq2 (v1.22.1), based on the reads count obtained by HTSeq (v0.9.1). DEGs in Figure S1 were extracted using the following criteria: EGA-ON genes in IVO4c, FC^IVO4c/IVO2c^ > 3, average FPKM in IVO4c > 5; PFF-OFF genes in IVO4c, FC^PFF/IVO4c^ > 3, average FPKM in PFF > 5. Both genes were classified into three groups based on the expression differences among IVO4c, PFF, and SCNT4c. We also defined FC^SCNT4c-KG/SCNT4c^ > 2 and FC^SCNT4c-TDG/SCNT4c^ > 2 as EGA-OFF genes reactivated in SCNT4c-KG/TDG, while FC^SCNT4c/SCNT4c-KG^ > 2 and FC^SCNT4c/SCNT4c-TDG^ > 2 were PFF-ON genes silenced in SCNT4c-KG/TDG. Adjusted p values of all DEGs were < 0.05. GO analysis was performed using clusterProfiler function in R.

### Identification of differentially expressed regions

The expression levels of genomic regions among samples were assessed through a sliding window (size 50 kb, step size 20 kb) made by bedtools, and normalized by RPM. Differentially expressed regions (DERs) in Figure 1 were extracted using the following criteria: EGA-ON regions in IVO4c, FC^IVO4c/IVO2c^ > 5, average RPM in IVO4c > 5; PFF-OFF regions in IVO4c, FC^PFF/IVO4c^ > 5, average RPM in PFF > 5. Both regions were classified into three groups based on the expression differences among IVO4c, PFF, and SCNT4c. We also defined FC^SCNT4c-KG/SCNT4c^ > 2 and FC^SCNT4c-TDG/SCNT4c^ > 2 as EGA-OFF regions reactivated in SCNT4c-KG/TDG, while FC^SCNT4c/SCNT4c-KG^ > 2 and FC^SCNT4c/SCNT4c-TDG^ > 2 were PFF-ON regions silenced in SCNT4c-KG/TDG. Fisher's exact test p values of all DERs were < 0.05.

### ULI-NChIP sequencing and data processing

ULI-NChIP was performed as previously described (Brind'Amour et al., 2015). For each immunoprecipitation reaction, 200 PFF or 50 four-cell embryos were added in Nuclear Isolation buffer and MNase Master Mix to digest chromatin for 7 min at 25°C. Chromatin was diluted in Complete Immunoprecipitation buffer, and incubated with 2 μg of antibody-bead complexes (H3K4me3, ab8580; H3K9me3, ab8898; H3K27me3, 07-449; Dynabeads Protein G, Invitrogen) overnight at 4°C. After washing with Low Salt Wash buffer and High Salt Wash buffer, DNA was eluted in Elution buffer for 2 h at 65°C, and purified by phenol chloroform. Raw ChIP material was then used for library construction by a KAPA HyperPrep Kit (Roche). Paired-end 150-bp sequencing was performed on an Illumina HiSeq X Ten platform.

Illumina Casava (v1.8.2) was used for base calling. Sequenced reads were trimmed for adapter sequence, low-quality, and low-complexity reads, then mapped to Sscrofa11.1 reference genome using Bowtie2 (v2.3.3.1). Multiple mapped reads, PCR duplicates, and reads with a map quality score of less than 30 were removed. The correlation coefficients of replicates, ChIP-seq intensity, and the occupying rates of exonic and repetitive sequences were quantified and visualized by deeptools (v3.1.3). Peak calling was performed by MACS2 (v2.2.5) with the parameters "macs2 callpeak –n Sample –g 2.2e9 -B -p 0.05" relative to input samples. Mapping reads of representative genomic regions were visualized using IGV software.

### PBAT sequencing and data processing

A pool of PFF and four-cell SCNT embryos (50 cells) was harvested for PBAT-seq as previously reported (Miura et al., 2012; Smallwood et al., 2014). In brief, cell lysate was bisulfite converted using a MethylCode Bisulfite Conversion Kit (Invitrogen), performing ten-round random priming by Klenow exo- (NEB) and scBS-seq-bio-P5-N9-oligo1 (CTA CAC GAC GCT CTT CCG ATC TNN NNN NNN N). After capturing biotinylated DNA on Dynabeads M-280 Streptavidin (Invitrogen), the second strand was synthesized using scBS-seq-P7-N9-oligo2 (AGA CGT GTG CTC TTC CGA TCT NNN NNN NNN). DNA was then amplified using 2× KAPA HiFi HotStart ReadyMix (Roche) and the index/universal primers (NEB). Paired-end 150-bp sequencing was performed on an Illumina NovaSeq 6000 platform.

Illumina Casava (v1.8.2) was used for base calling. Trimmed reads were then mapped to Sscrofa11.1 reference genome using bismark (v0.22.3). Multiple mapped reads, PCR duplicates, and reads with a map quality score of less than 30 were removed. The DNA methylation levels were quantified and visualized by bismark and deeptools (v3.1.3), respectively.

### Statistical analysis

All experiments were repeated at least three times. All middle lines in the box-whisker plots indicate the median, the edges indicate the 25^th^/75^th^ percentiles, and the whiskers indicate the 2.5^th^/97.5^th^ percentiles. Development rates and qPCR results are presented as the mean ± SD. p values were calculated using two-tailed Student's t test with SPSS Statistics 20 software (IBM). Differences are shown with asterisks indicating ^∗^p < 0.05, ^∗∗^p < 0.01, and ^∗∗∗^p < 0.001.

### Data and code availability

The Gene Expression Omnibus accession numbers for the data reported in this paper are GEO: GSE161527 and GSE139512.

## Author contributions

Conceptualization, Y.-L.M., Z.-X.C., and X.L.; methodology, X.L., T.W., L.C., and J. Zhou; software, Z.-X.C., L.C., J. Zhang, and W.Z.; validation, X.L. and J. Zhou; formal analysis, X.L., T.W., L.C., and J. Zhou; investigation, T.W., Z. Li, G.B., S.Y., D.W., C.D., T.X., H.H., L.Y., Z. Liu, and X.Z.; resources, T.W., Z. Li, G.B., S.Y., D.W., C.D., T.X., H.H., L.Y., Z. Liu, and X.Z.; data curation, L.C. and J. Zhang; writing – original draft, X.L., T.W., and L.C.; writing – review and editing, Y.-L.M., X.L., T.W., and L.C.; visualization, X.L., L.C., J. Zhang, and W.Z.; supervision and project administration, Y.-L.M. and Z.-X.C.; funding acquisition, Y.-L.M.

## Conflicts of interests

The authors declare no competing interests.

## Supplemental information

Document S1. Figures S1–S7, Tables S2 and S6

Table S1. Summary of RNA-seq and ChIP-seq experiments, related to Figure 1

Table S3. Expression levels of EGA-ON and PFF-OFF genes among porcine PFF, IVO, and SCNT embryos, related to Figures 2, 4, and 7

Table S4. Expression levels of EGA-ON and PFF-OFF regions among porcine PFF, IVO, and SCNT embryos, related to Figures 1, 4, and 7

Table S5. Expression levels of porcine EGA-ON repetitive elements, related to Figure 5

Document S2. Article plus supplemental information
