# Supplementary material for: TDG is a pig-specific epigenetic regulator with insensitivity to H3K9 and H3K27 demethylation in nuclear transfer embryos
Source: Stem Cell Reports. 2021 Oct 21;16(11):2674–89. doi: 10.1016/j.stemcr.2021.09.012 (PMC8581057; doi:10.1016/j.stemcr.2021.09.012)
Supplement: Document S1. Figures S1–S7, Tables S2 and S6 [file mmc1.pdf]

Stem Cell Reports, Volume *16*

Supplemental Information

TDG is a pig-specific epigenetic regulator with insensitivity to H3K9 and H3K27 demethylation in nuclear transfer embryos

Xin Liu, Lu Chen, Tao Wang, Jilong Zhou, Zhekun Li, Guowei Bu, Jingjing Zhang, Shuyuan Yin, Danya Wu, Chengli Dou, Tian Xu, Hainan He, Wei Zhu, Longtao Yu, Zhiting Liu, Xia Zhang, Zhen-Xia Chen, and Yi-Liang Miao

Supplemental Figures

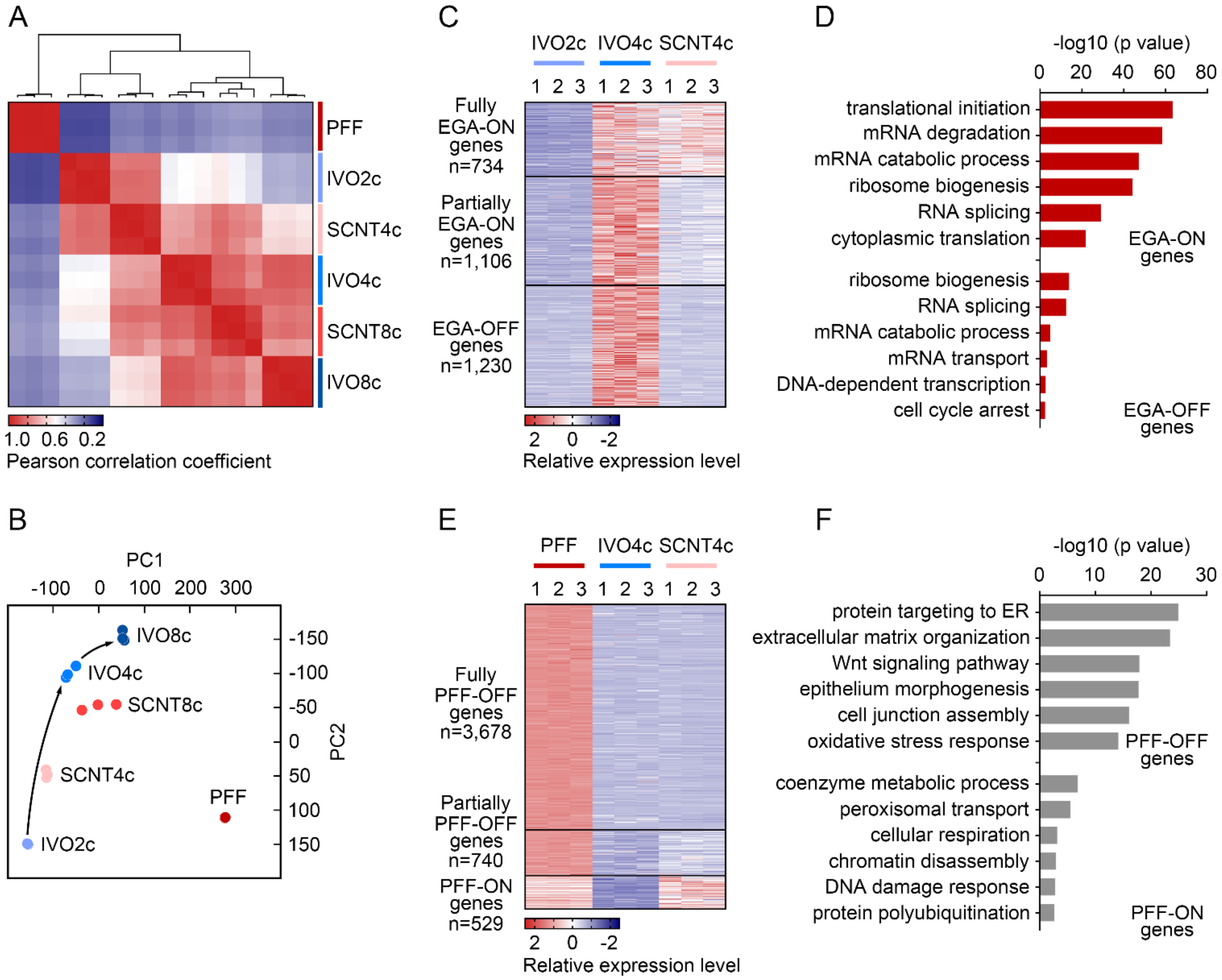

Figure S1. Differentially expressed genes in four-cell SCNT embryos, related to Figure 2.

(A) Heatmap showing the Pearson correlation coefficients in PFF, IVO and SCNT embryos at different stages. Hierarchical clustering is shown in the top panel. 2c, 4c, 8c indicates 2-cell, 4-cell, 8-cell embryos, respectively. RNA-seq data of IVO embryos are from a previous study (Kong et al., 2020).

(B) Principal component analysis of expression patterns among all samples.

(C and E) Heatmap showing the expression levels of differentially expressed genes (DEGs) [fold change (FC) > 3] between IVO2c/PFF and IVO4c, which are classified into six groups by comparing SCNT4c with IVO4c (Fully EGA-ON, FC ≤ 2; Partially EGA-ON, 2 < FC ≤ 5; EGA-OFF, FC > 5) and PFF (Fully PFF-OFF, FC ≥ 5; Partially PFF-OFF, 2 ≤ FC < 5; PFF-ON, FC < 2). Each row represents the normalized FPKM (fragments per kilobase of exon per million mapped fragments) of a transcript and each column represents a replicate.

(D and F) Bar plots showing the gene ontology analysis of EGA-ON/OFF and PFF-ON/OFF genes.

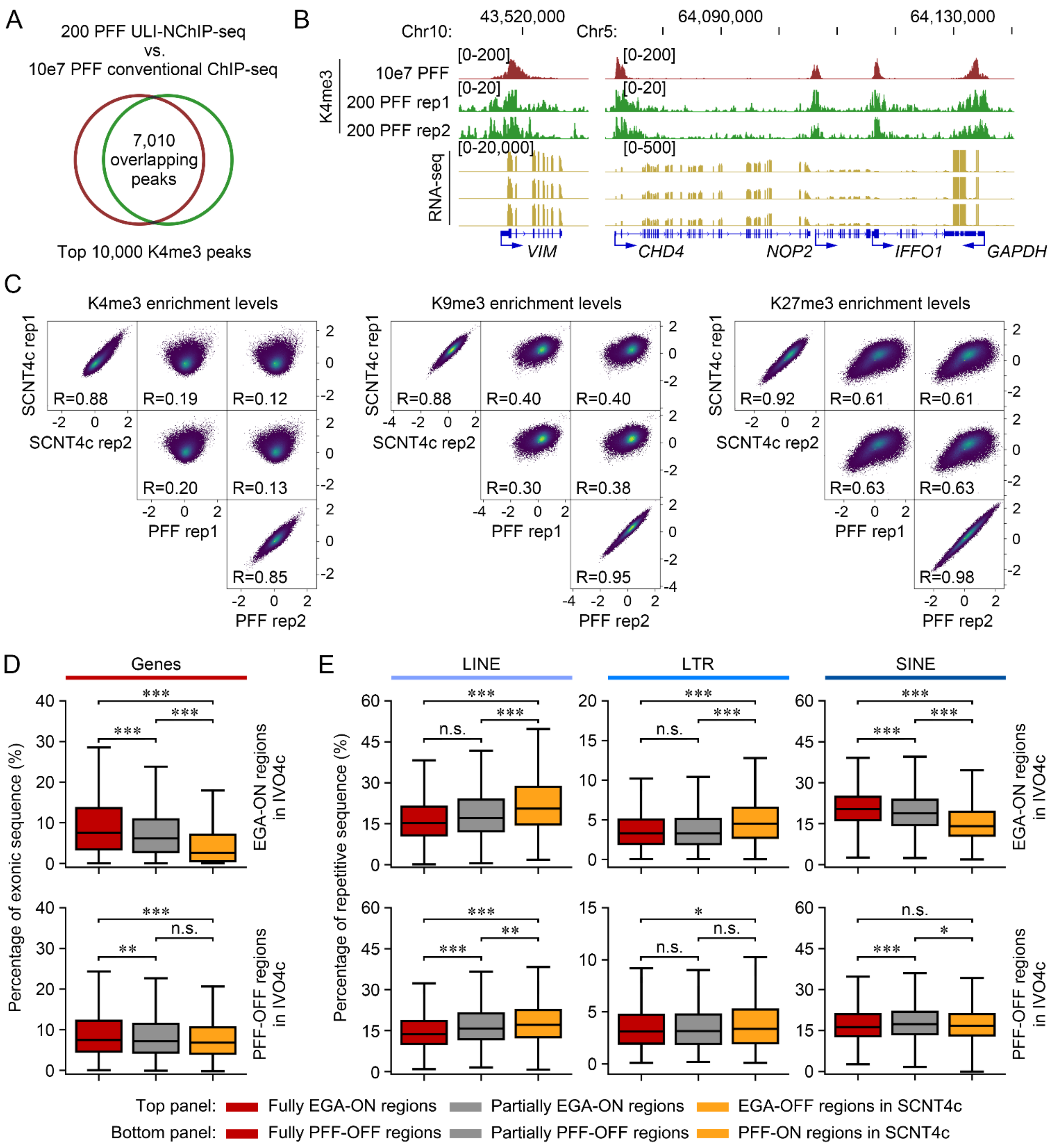

Figure S2. Validation and high reproducibility of ULI-NChIP-seq, related to Figure 1.

(A) The number of overlapping peaks between the top 10,000 H3K4me3 peaks detected from ultra-low-input native chromatin immunoprecipitation sequencing (ULI-NChIP-seq) using 200 PFF and conventional ChIP-seq using 10e7 PFF (Gao et al., 2019).

(B) Genome browser view showing H3K4me3 peaks on the transcription start sites (TSSs) of representative genes from ULI-NChIP-seq and conventional ChIP-seq.

(C) Scatter plots showing the correlations of H3K4me3, H3K9me3, H3K27me3 enrichments among ULI-NChIP-seq samples.

(D) Box plots comparing the average percentage of exonic sequences in EGA-ON/OFF and PFF-ON/OFF

regions in SCNT4c. **p < 0.01, *** p < 0.001; n.s., not significant; two-tailed Student's *t* test.

(E) Box plots comparing the average percentage of repetitive sequences in EGA-ON/OFF and PFF-ON/OFF regions in SCNT4c. *p < 0.05, **p < 0.01, ***p < 0.001; n.s., not significant; two-tailed Student's *t* test.

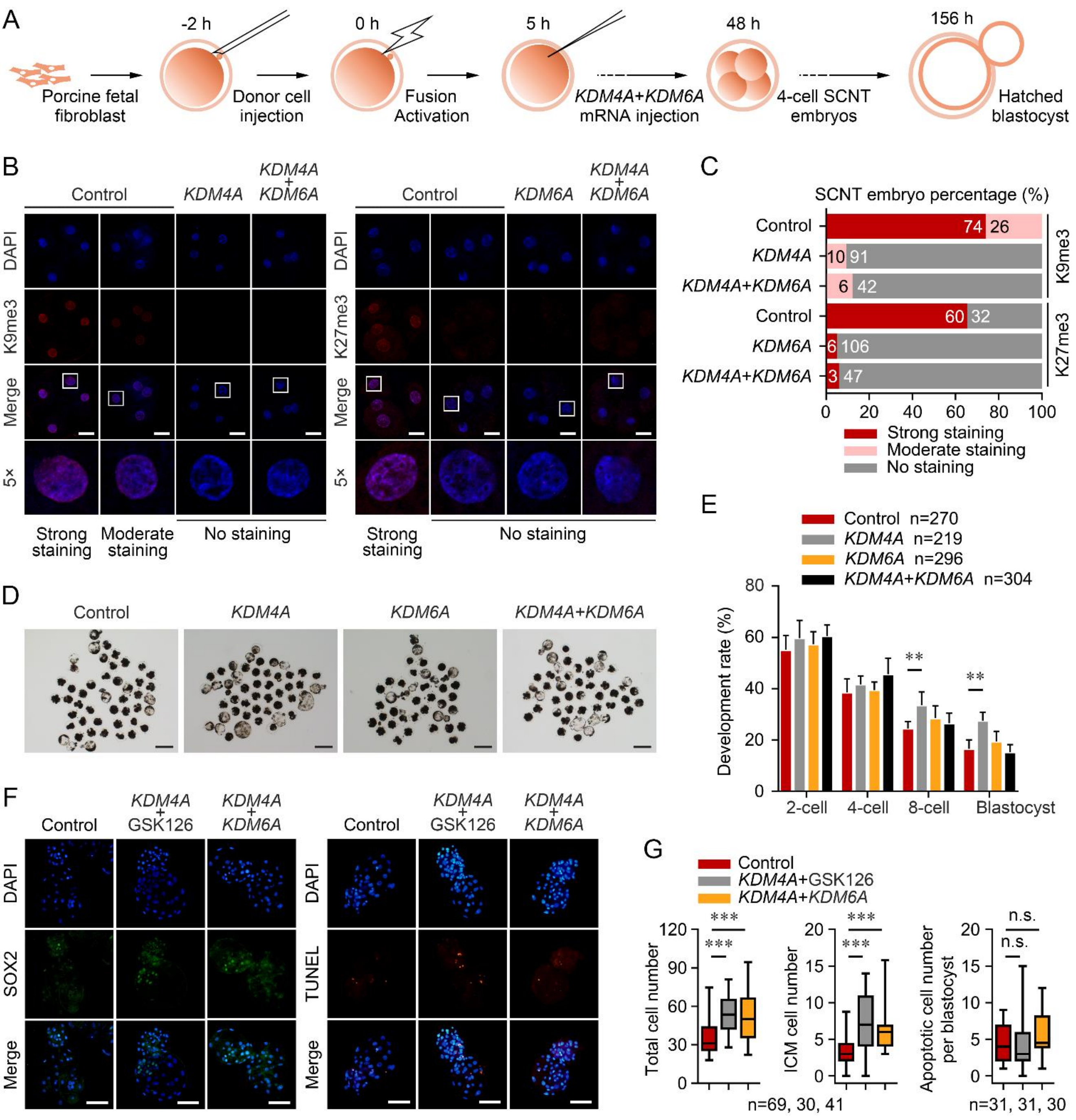

Figure S3. *KDM4A+KDM6A* co-injection cannot improve SCNT embryonic development, related to Figure 3.

(A) Experimental design of the microinjection procedure. SCNT embryos were injected with *KDM4A* and *KDM6A* mRNA at 5 h after activation.

(B) Immunostaining of H3K9me3 and H3K27me3 (red) and DNA (blue) in SCNT4c derived from 1,000 ng/μL *KDM4A*-injected, *KDM6A*-injected, co-injected and non-injected groups. One of the nuclei in SCNT4c is magnified five-fold. Scale bars, 50 μm.

(C) Bar plot showing the percentage of SCNT4c with positive H3K9me3 and H3K27me3 staining and no staining in different groups. Numbers of the total embryos analyzed from 4-5 replicates are shown in the bars.

(D) Representative images of different groups after culturing for 6.5 d *in vitro.* Scale bar, 200 μm.

(E) Bar plot showing the development rates in different groups. Error bars represent the SD. Numbers of the total

embryos analyzed from five replicates are shown in the legend. $**p < 0.01$; two-tailed Student's *t* test.

(F) Labeling of SOX2 (green), apoptotic cells (red) and DNA (blue) in blastocysts derived from *KDM4A*+GSK126 combined-treated, *KDM4A+KDM6A* co-injected and non-treated groups. Scale bars, 100 μm.

(G) Box plots comparing the total cell numbers, inner cell mass (ICM) cell numbers, and apoptotic cell numbers of blastocysts derived from different groups. Numbers of the total blastocysts analyzed from three replicates are shown under the plot. $***p < 0.001$; n.s., not significant; two-tailed Student's *t* test.

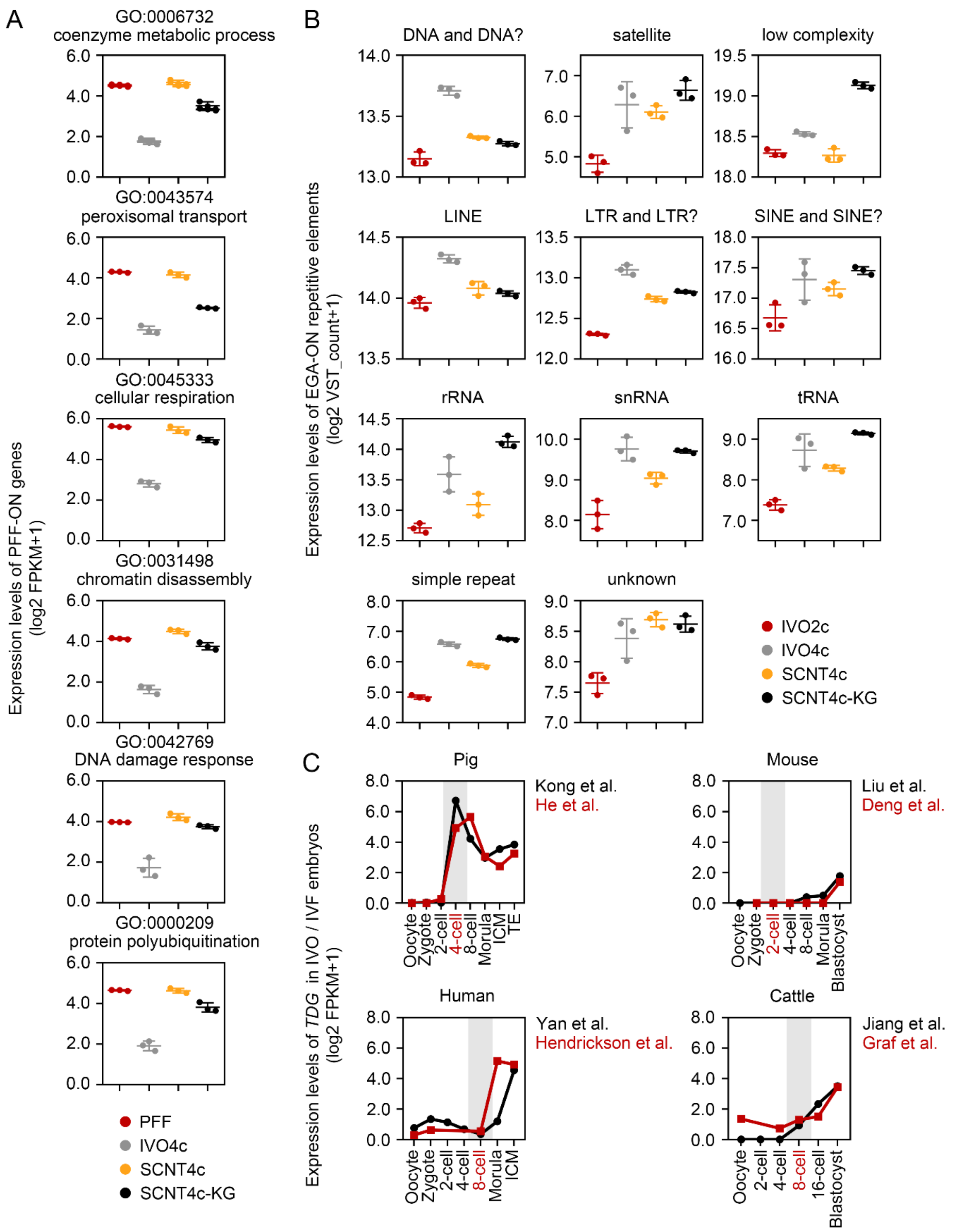

Figure S4. Expression levels of PFF-ON genes, EGA-ON repetitive elements and *TDG*, related to Figures 4-6.

(A) Scatter dot plots comparing the expression levels of candidate PFF-ON genes (Figure S1F) in PFF, IVO4c, SCNT4c and SCNT4c with combined *KDM4A*+GSK126 treatment (SCNT4c-KG). Each plot shows the mean and the SD error bars in three replicates.

(B) Scatter dot plots comparing the expression levels of each EGA-ON repetitive element in IVO2c, IVO4c, SCNT4c and SCNT4c-KG. Each plot shows the mean and the SD error bars in three replicates.

(C) Line plots illustrating the dynamic transcriptional changes of *TDG* in the fertilized embryos of pig (He et al., 2019; Kong et al., 2020), mouse (Deng et al., 2014; Liu et al., 2016), human (Hendrickson et al., 2017; Yan et al., 2013) and cattle (Graf et al., 2014; Jiang et al., 2014). Developmental stage colored in red with gray shade indicates the EGA timing in each species.

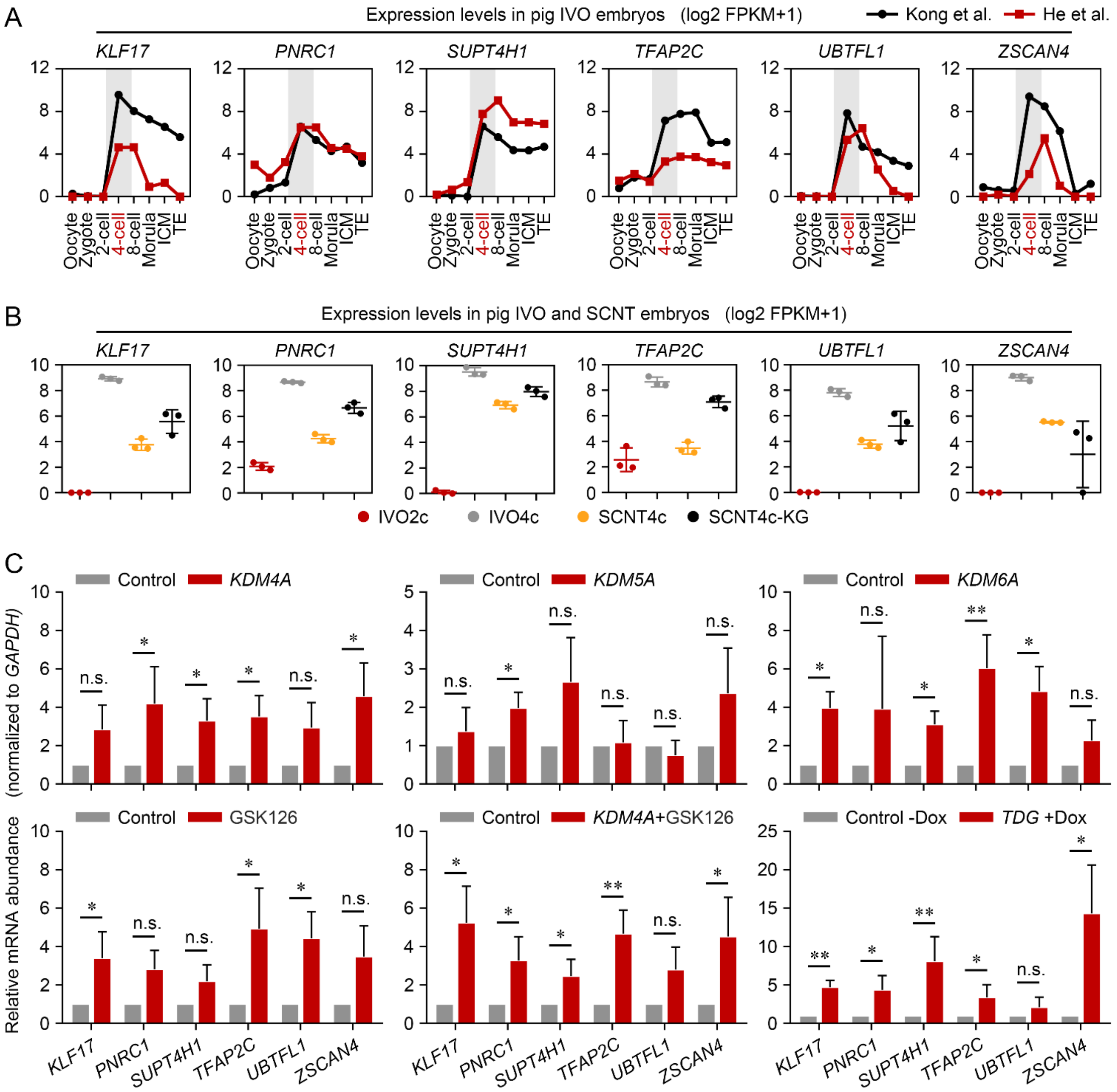

Figure S5. Expression levels of six EGA-OFF genes in four-cell SCNT embryos, related to Figures 4 and 6.

(A) Line plots illustrating the dynamic transcriptional changes of six genes in porcine IVO embryos (He et al., 2019; Kong et al., 2020). Developmental stage colored in red with gray shade indicates the EGA timing in porcine IVO embryos.

(B) Scatter dot plots comparing the expression levels of six genes in IVO2c, IVO4c, SCNT4c and SCNT4c-KG. Each plot shows the mean and the SD error bars in three replicates.

(C) Bar plots showing the mRNA abundances of six EGA-OFF genes in SCNT4c derived from *KDM4A*+GSK126 combined-treated, *TDG* +Dox, *KDM4A*-injected, *KDM5A*-injected, *KDM6A*-injected, GSK126-treated and their corresponding non-treated groups. The results from non-treated groups are set as 1. Error bars represent the SD in 3-4 replicates. *p < 0.05, **p < 0.01; n.s., not significant; two-tailed Student's *t* test.

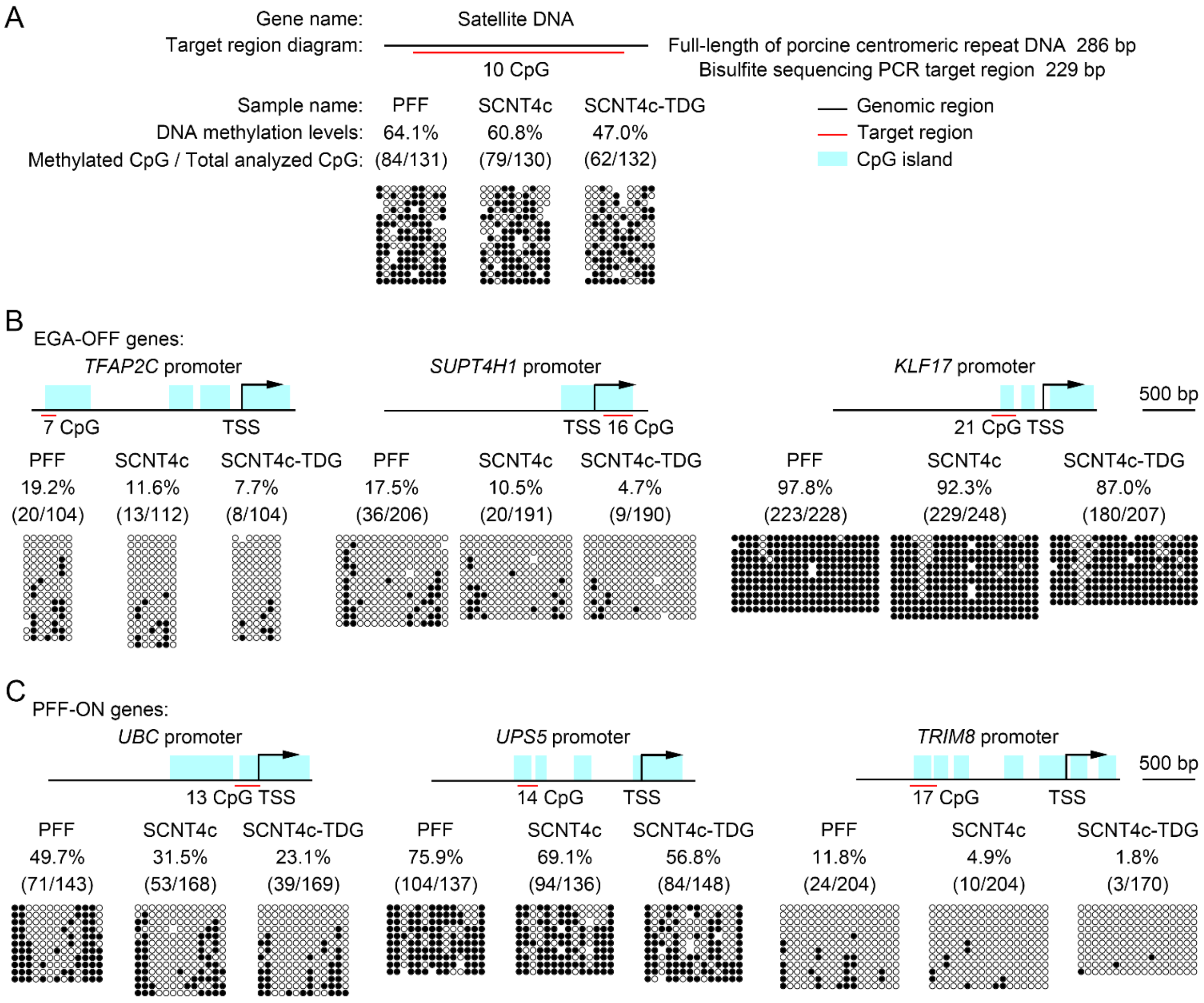

Figure S6. TDG reduces DNA methylation levels in four-cell SCNT embryos, related to Figures 6 and 7. (A-C) DNA methylation statuses of satellite DNA (A), EGA-OFF gene promoters (B) and PFF-ON gene promoters (C) in PFF, SCNT4c and SCNT4c overexpressed with *TDG* (SCNT4c-TDG). DNA methylation levels are determined by bisulfite sequencing PCR (BS-PCR), and are shown as the percentage of methylated CpG relative to the total analyzed CpG. Numbers in parentheses indicate methylated CpG number and total analyzed CpG number, respectively. White and black circles represent unmethylated and methylated CpG, respectively. Each horizontal line represents one individual clone. The diagram in each figure denotes the BS-PCR target regions (red lines) near CpG islands (blue regions) in gene promoters (upstream 2,000 bp and downstream 500 bp from TSSs). CpG number in target region is shown under red lines.

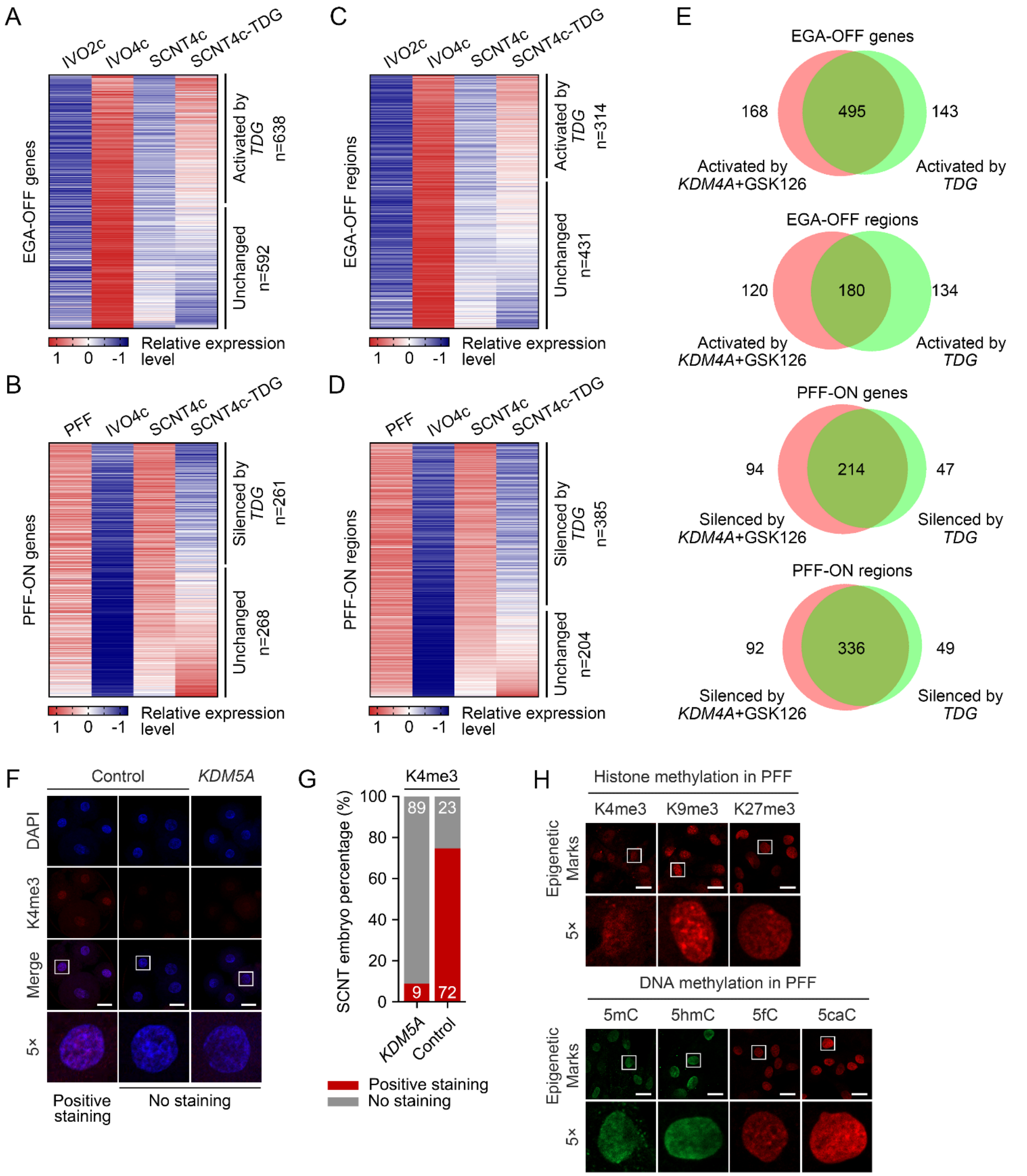

Figure S7. TDG facilitates EGA initiation and somatic cell memory silencing, related to Figures 6 and 7.

(A and B) Heatmap showing the expression levels of EGA-OFF genes (Figure S1C) among IVO2c, IVO4c, SCNT4c, SCNT4c-TDG, and PFF-ON genes (Figure S1E) among PFF, IVO4c, SCNT4c, SCNT4c-TDG. Each row represents the normalized FPKM of a transcript. FC > 2 are EGA-OFF genes reactivated in SCNT4c-TDG (SCNT4c-TDG versus SCNT4c), FC < 0.5 are PFF-ON genes silenced in SCNT4c-TDG.

(C and D) Heatmap showing the expression levels of EGA-OFF regions (Figure 1A) among IVO2c, IVO4c, SCNT4c, SCNT4c-TDG, and PFF-ON regions (Figure 1B) among PFF, IVO4c, SCNT4c, SCNT4c-TDG. Each

row represents the normalized RPM of a region. FC > 2 are EGA-OFF regions reactivated in SCNT4c-TDG (SCNT4c-TDG versus SCNT4c), FC < 0.5 are PFF-ON regions silenced in SCNT4c-TDG.

(E) The overlapping number of restored genes and regions between SCNT4c-KG and SCNT4c-TDG.

(F) Immunostaining of H3K4me3 (red) and DNA (blue) in SCNT4c derived from 1,000 ng/μL *KDM5A*-injected and non-injected groups. One of the nuclei in SCNT4c is magnified five-fold. Scale bars, 50 μm.

(G) Bar plot showing the percentage of SCNT4c derived from different groups with positive H3K4me3 staining and no staining. Numbers of the total embryos analyzed from four replicates are shown in the bars.

(H) Immunostaining of H3K4me3 (red), H3K9me3 (red), H3K27me3 (red), 5mC (green), 5hmC (green), 5fC (red), and 5caC (red) in donor cell PFF. One of the nuclei in PFF is magnified five-fold. Scale bars, 20 μm.

Supplemental Tables

Table S1. RNA-seq and ChIP-seq experiment summary, related to Figure 1.

See supplemental Excel file.

Table S2. ***In vitro*** **development of SCNT embryos with different treatments, related to Figures 3 and 6.**

Group	Treatment	Number of replicates	Number of fused one-cell embryos	Cleaved per one-cell (%)	Four-cell per one-cell (%)	Eight-cell per one-cell (%)	Blastocyst per one-cell (%)
Experiment 1	α-amanitin treatment 20 μg/mL	5	276	60.42 ± 5.03 [a]	42.28 ± 4.64 [a]	6.63 ± 1.04 [a]	0.86 ± 1.23 [a]
	DMSO treatment	5	229	54.99 ± 4.79 [a]	40.42 ± 5.46 [a]	27.76 ± 4.38 [b]	20.98 ± 4.18 [b]
	No treatment Control	5	208	58.48 ± 6.36 [a]	43.01 ± 4.03 [a]	26.48 ± 3.61 [b]	18.01 ± 3.47 [b]
Experiment 2	*KDM4A* mRNA injection						
	1,000 ng/uL	3	139	47.66 ± 4.18 [a]	N.A.	N.A.	23.57 ± 4.01 [ab]
	500 ng/uL	3	109	54.01 ± 4.47 [a]	N.A.	N.A.	25.94 ± 3.11 [a]
	100 ng/uL	3	110	53.70 ± 6.42 [a]	N.A.	N.A.	21.66 ± 6.58 [ab]
	20 ng/uL	3	111	55.37 ± 8.06 [a]	N.A.	N.A.	16.30 ± 4.63 [bc]
	Non-injected Control	3	135	48.77 ± 2.93 [a]	N.A.	N.A.	12.44 ± 3.80 [c]
Experiment 3	*KDM5A* mRNA injection						
	1,000 ng/uL	3	142	58.43 ± 4.94 [a]	N.A.	N.A.	12.84 ± 3.94 [a]
	500 ng/uL	3	100	56.32 ± 4.51 [a]	N.A.	N.A.	14.21 ± 2.63 [a]
	100 ng/uL	3	110	55.42 ± 7.01 [a]	N.A.	N.A.	19.58 ± 4.17 [a]
	20 ng/uL	3	104	62.71 ± 2.82 [a]	N.A.	N.A.	18.54 ± 3.44 [a]
	Non-injected Control	3	130	61.12 ± 6.33 [a]	N.A.	N.A.	14.57 ± 4.04 [a]

Experiment 4	*KDM6A* mRNA injection						
	1,000 ng/uL	3	138	56.10 ± 7.96 ^{ab}	N.A.	N.A.	19.02 ± 5.46 ^{a}
	500 ng/uL	3	129	54.77 ± 7.77 ^{ab}	N.A.	N.A.	21.69 ± 2.15 ^{a}
	100 ng/uL	3	120	64.90 ± 1.14 ^{a}	N.A.	N.A.	24.62 ± 3.53 ^{a}
	20 ng/uL	3	150	52.62 ± 2.92 ^{b}	N.A.	N.A.	23.28 ± 4.63 ^{a}
	Non-injected Control	3	120	57.08 ± 5.20 ^{ab}	N.A.	N.A.	17.11 ± 4.69 ^{a}
Experiment 5	*KDM4A* mRNA injection 1,000 ng/uL	5	219	59.75 ± 6.91 ^{a}	41.69 ± 3.31 ^{ab}	33.61 ± 5.14 ^{a}	27.53 ± 3.30 ^{a}
	KDM6A mRNA injection 1,000 ng/uL	5	296	57.28 ± 4.95 ^{a}	39.50 ± 3.11 ^{ab}	28.51 ± 4.80 ^{ab}	19.46 ± 3.89 ^{b}
	KDM4A + *KDM6A* mRNA co-injection	5	304	60.53 ± 4.29 ^{a}	45.64 ± 6.25 ^{a}	26.51 ± 3.92 ^{b}	15.29 ± 2.87 ^{b}
	Non-injected Control	5	270	55.16 ± 5.59 ^{a}	38.58 ± 5.28 ^{b}	24.51 ± 2.66 ^{b}	16.56 ± 3.51 ^{b}
Experiment 6	GSK126 treatment						
	0.05 μM for 48 h	3	130	57.10 ± 4.31 ^{a}	N.A.	N.A.	24.09 ± 2.07 ^{ab}
	0.1 μM for 48 h	3	110	61.84 ± 5.47 ^{a}	N.A.	N.A.	28.68 ± 3.67 ^{b}
	0.5 μM for 48 h	3	135	62.65 ± 7.16 ^{a}	N.A.	N.A.	25.85 ± 4.90 ^{b}
	No treatment Control	3	120	54.61 ± 7.41 ^{a}	N.A.	N.A.	16.82 ± 3.38 ^{a}
Experiment 7	*KDM4A* mRNA injection 1,000 ng/uL	5	194	59.47 ± 5.41 ^{a}	44.40 ± 3.55 ^{ab}	34.43 ± 2.77 ^{a}	26.78 ± 3.04 ^{a}
	GSK126 treatment 0.1 μM for 48 h	5	219	61.89 ± 3.63 ^{a}	42.42 ± 2.34 ^{ab}	31.60 ± 3.93 ^{a}	24.71 ± 3.47 ^{a}
	KDM4A mRNA injection + GSK126 treatment	5	227	63.31 ± 3.78 ^{a}	47.34 ± 5.21 ^{a}	39.70 ± 3.70 ^{b}	33.49 ± 4.22 ^{b}

	No treatment Control	5	181	58.67 ± 6.48 ^{a}	41.13 ± 3.09 ^{b}	26.04 ± 4.76 ^{c}	15.97 ± 2.07 ^{c}
Experiment 8	*TDG* mRNA injection						
	1,000 ng/uL	3	156	26.52 ± 6.53 ^{a}	N.A.	N.A.	0.00 ± 0.00 ^{a}
	500 ng/uL	3	109	46.82 ± 4.50 ^{b}	N.A.	N.A.	0.93 ± 1.60 ^{a}
	100 ng/uL	3	93	50.57 ± 5.42 ^{bc}	N.A.	N.A.	12.11 ± 4.98 ^{b}
	20 ng/uL	3	157	54.11 ± 4.34 ^{bc}	N.A.	N.A.	15.49 ± 3.43 ^{b}
	Non-injected Control	3	148	58.67 ± 7.83 ^{c}	N.A.	N.A.	18.34 ± 4.60 ^{b}
Experiment 9	Dox-inducible *TDG* expression						
	TDG donor cell +Dox	4	167	48.64 ± 3.37 ^{a}	36.29 ± 2.85 ^{a}	28.84 ± 5.04 ^{a}	24.97 ± 2.71 ^{a}
	TDG donor cell -Dox	4	160	50.76 ± 4.81 ^{ab}	32.75 ± 3.94 ^{a}	21.83 ± 1.81 ^{b}	10.49 ± 4.11 ^{b}
	Control donor cell +Dox	4	138	54.17 ± 4.02 ^{ab}	37.34 ± 4.55 ^{a}	23.01 ± 4.80 ^{ab}	12.67 ± 5.70 ^{b}
	Control donor cell -Dox	4	132	56.94 ± 4.73 ^{b}	39.06 ± 4.77 ^{a}	25.70 ± 1.76 ^{ab}	16.20 ± 2.81 ^{b}
Experiment 10	*KDM4A* + GSK126 + *TDG* (Triple Treatment)	3	128	64.00 ± 5.12 ^{a}	52.34 ± 5.05 ^{a}	43.00 ± 3.28 ^{a}	34.44 ± 3.09 ^{a}
	KDM4A mRNA injection + GSK126 treatment	3	121	69.75 ± 3.81 ^{a}	55.66 ± 4.29 ^{a}	44.69 ± 2.26 ^{a}	38.65 ± 3.87 ^{a}
	Non-treated Control	3	115	66.95 ± 6.55 ^{a}	49.76 ± 3.04 ^{a}	27.52 ± 2.40 ^{b}	17.36 ± 3.87 ^{b}

Data are shown as the mean ± SD. N.A., not available.

[a, b, c] Values with different superscripts differ significantly ($p < 0.05$) in the same group from each other (one-way ANOVA with Duncan test).

Table S3. Expression levels of EGA-ON and PFF-OFF genes among porcine PFF, IVO and SCNT embryos, related to Figures 2, 4, 7.

See supplemental Excel file.

Table S4. Expression levels of EGA-ON and PFF-OFF regions among porcine PFF, IVO and SCNT embryos, related to Figures 1, 4, 7.

See supplemental Excel file.

Table S5. Expression levels of porcine EGA-ON repetitive elements, related to Figure 5.

See supplemental Excel file.

Table S6. Sequences of cDNA amplification primers, quantitative PCR primers, and bisulfite sequencing PCR primers, related to Supplemental Experimental Procedures.

Name	Gene Accession	Experiments	Sense (5′ to 3′)	Antisense (5′ to 3′)
KDM4A	XM_021096835.1	Full-length cDNA amplification	GGAGGGAAATGGCTTCTGAATCTGAAAC	CCGCTGGAAGCACCTACTCCATGATG
		Amplification with T7 promotor primers	GCGAAATTAATACGACTCACTATAGGGAGAGCCACCATGGCTTCTGAATCTGAAAC	GCCGGTTTAGACTACTCCATGATGGCCCGGTACAGCGCAGGCTCGATGTAATC
KDM5A	XM_021092486.1	Full-length cDNA amplification	GCCCCGACCTGAGAGGAAAAG	CCAGCCACCCCAGAACTCAATC
		Amplification with T7 promotor primers	GGCAGCTCGAAATTAATACGACTCACTATAGGGAGACCCAAGCCACCATGGCGGGCATTGGGCCGGGGGGCTACGCGGCGGAGTTTG	GCGGGTTTAAACTAACTGGTCTCTTTAAGATCCTCCACTGGTAGTTTG
KDM6A	XM_021079778.1	Full-length cDNA amplification	GGTGTCGGCGTTGGAGTTGTGA	GTCTGTATGATGTTGCCAGTTTTG
		Amplification with T7 promotor primers	GCGAAATTAATACGACTCACTATAGGGAGAGCCACCATGAAATCCTGCGGAGTGTCGCTCGCTACCGCCGCCGCTGCCGCCGCCGCTTTCGGTGATGAGGAAAAG	GCGGGTTTGAATCAAGATGAGGAGGATGGTAATGGAGGAGCTAATG
TDG	XM_021092634.1	Full-length cDNA amplification	GCCATCGAGTCCAAGAGACTG	GCAGGGCTGAGAAACACTAGTC
		Amplification with T7 promotor primers	GCGAAATTAATACGACTCACTATAGGGAGAGCCACCATGGAAGCAGAGAACGCGAGCAGCTATTC	GCGGGTTTAACTTAAGCACAGTTTCCTTCTTCCTGCTCTTGCGTCCCACGGTGATTGTTAAAG
KLF17	NM_001164010.1	qPCR	AAGATTATGAGGCAGGGTGGAA	CGGAAAACTGTCAAAGACCAAGA
PNRC1	XM_021089951.1	qPCR	CCACCCTCTTTCTCCCTCACT	CTTCTTTCGCCGCTTCTTTGG

SUPT4H1	XM_003131646.4	qPCR	GCTTGTTTGCTGTGTTCGCT	TGTAGGCCACTCCTCGACTT
TFAP2C	NM_001123201.1	qPCR	GATCAGACGGTCATTCGCAAAG	AGAAAACCTCGCTGGGATTCAT
UBTFL1	XM_021102676.1	qPCR	GGCAGTGGGGATGAAGATGAAT	CTTTTGGTGGGAAGTCCTCTGT
ZSCAN4	XM_021097584.1	qPCR	ACAGACGATGGCATGAAGCA	TCGAGGAGTCCAGAGAGGTG
GAPDH	NM_001206359.1	qPCR	TCGGAGTGAACGGATTTG	CCTGGAAGATGGTGATGG
Satellite DNA	Z75640.1	BS-PCR	TTTGTAGAATGTAGTTTTTAGAAG	AAAATCTAAACTACCTCTAACTC
TFAP2C promoter	NM_001123201.1	BS-PCR	AAGGGTAATTAGGATTTGGGGT	CCTAAAATACAACTTAAACAAATTATCT
KLF17 promoter	NM_001164010.1	BS-PCR	TGATGAAAGTTTGATTTTTTTTGTTAGT	CCCTACTCCATCTCAACCTAAAAT
SUPT4H1 promoter	XM_003131646.4	BS-PCR	AGAGGATGGTTTTGGAGATAGTGT	CCTATACTAAAAACTAAAATTACAAAAACT
TRIM8 promoter	XM_001928904.7	BS-PCR	TTTAATTTTAGATAGGGAAAAGGGG	AAAAATAATATCCCTACATCAAAAC
UBC promoter	XM_003483411.4	BS-PCR	TATAGGAAAGTATTTTAGGAGTGAAA	CCTCACCAAATAACAATAACAACAA
USP5 promoter	XM_021092411.1	BS-PCR	GTTTAGGGTATTTGGATGGGAGT	ACCTAAATCACTTCTCACAAACATC

Supplemental Experimental Procedures

Donor cell preparation

Passage 2-5 PFFs were cultured in DMEM medium (Gibco) containing 10% (v/v) fetal bovine serum (Hyclone) for 72 h to induce contact inhibition, and digested by trypsin-EDTA solution to use as donor cells. For cell line establishment, PFFs were transfected with *TDG*-inducible expression vector by using the U023 program on Nucleofector 2b (Lonza), and then selected in culture medium containing 2 μg/mL puromycin for 7 d. The vector construction is based on TLCV2 backbone (Addgene plasmid # 87360).

SCNT

Matured oocytes were collected as a previous report (Liu et al., 2020). Enucleation was then performed with a 20-μm glass pipette by aspirating the first polar body and adjacent cytoplasm of oocytes in manipulation medium containing 7.5 mg/mL cytochalasin B, and a single donor cell was injected into the perivitelline space. Reconstructed embryos were placed in an activation chamber with electrodes 1 mm apart containing activating medium. Two successive direct current pulses (1.2 kV/cm, 30 μs) were applied on an Electro-Cell Manipulator ECM2100 (BTX). After activation (0 h), embryos were cultured in porcine zygote medium-3 (PZM-3) medium (Liu et al., 2020) for 6.5 d under 5% CO_2 in air at 38.5°C, and captured by using Ti-E microscopy (Nikon).

Microinjection and treatment

Full lengths of *KDM4A*, *KDM5A*, *KDM6A* and *TDG* were amplified from pig ovarian cDNA with primers containing T7 promoters by PrimeSTAR GXL DNA Polymerase (Takara) (Table S6). mRNAs were synthesized by mMESSAGE mMACHINE T7 Ultra Kit (Invitrogen) according to the manufacturer's instructions. For microinjection, 5 h-post-activated SCNT embryos were placed in manipulation medium and injected with 20-1,000 ng/μl mRNAs by FemtoJet 4i (Eppendorf), and cultured in PZM-3 medium for 6.5 d. For treatment, reconstructed embryos were cultured in PZM-3 medium containing 0.05-0.5 μM GSK126 (S7061, Selleck) at 0-48 h, 500 ng/mL doxycycline (631311, Clontech) at 40-72 h, or 20 μg/mL α-amanitin (A2236, Sigma-Aldrich) for 6.5 d.

Outgrowth

Outgrowth experiment of pig blastocysts was performed according to a previous study (Zhang et al., 2019). Briefly, SCNT blastocysts cultured for 7 d were seeded on mitotically inactivated mouse embryonic fibroblasts in KO-DMEM-based pig pluripotent stem cell medium. After culturing for 7 d under 5% O_2, 5% CO_2, 90% N_2 at 39°C, the outgrowth colonies were fixed in 4% paraformaldehyde for 15 min, stained with alkaline phosphatase by Fast Red TR/α-Naphthol AS-MX mixture (Sigma-Aldrich) for 15 min, and captured under bright-field microscopy.

Quantitative PCR

Total RNA was isolated from 30 four-cell embryos by RNAprep Pure Micro Kit (TIANGEN). First-strand cDNAs were synthesized by HiScript II Q RT SuperMix Kit plus gDNA wiper (Vazyme), and quantified by ChamQ Universal SYBR qPCR Master Mix (Vazyme) on CFX96 Real-Time PCR Detection System (Bio-Rad). The results were normalized to the internal control gene *GAPDH* (Kuijk et al., 2007). Primer sequences are listed in Table S6. Data are shown as the fold change = $2^{-\Delta\Delta Ct}$ mean ± SD.

Immunofluorescence

For histone methylation staining, samples were fixed in 4% paraformaldehyde for 30 min, permeabilized in 0.5% Triton X-100 for 30 min, blocked in 5% bovine serum albumin for 2 h, and incubated with primary antibodies (H3K4me3, ab8580, Abcam; H3K9me3, ab8898, Abcam; H3K27me3, 07-449, Merck; 1:500) overnight at 4°C. After three washes, embryos were incubated with corresponding secondary antibodies (Abbkine) for 1 h. For

DNA methylation staining, permeabilized samples were denatured with 4 N HCl for 10 min, neutralized with 100 mM Tris-HCl (pH 8.5) for 20 min, and incubated with primary (5mC, 39649; 5hmC, 39999; 5fC, 61228; 5caC, 61230; Active Motif; 1:100) and secondary antibodies as mentioned above. For cell number counting, permeabilized blastocysts were blocked and incubated with SOX2 antibody (sc-365823, Santa Cruz; 1:200). The TUNEL signals of permeabilized blastocysts were detected by using One Step TUNEL Apoptosis Assay Kit (Beyotime). All samples were mounted on glass slides in VECTASHIELD antifade medium (Vector Laboratories) containing 4,6-diamidino-2-phenylindole (DAPI), and captured on an LSM 800 confocal microscope (ZEISS). All steps were performed at room temperature unless stated otherwise.

Bisulfite sequencing PCR

A pool of 30 SCNT embryos and 96-well PFFs was harvested and bisulfite conversion was conducted by using EZ DNA Methylation-Direct Kit (Zymo Research) according to the manufacturer's instructions. Bisulfite sequencing PCR (BS-PCR) was then performed by using ZymoTaq DNA Polymerase (Zymo Research) and the primers designed from MethPrimer (v2.0 and v1.0) based on candidate gene promoter sequences (upstream 2,000 bp and downstream 500 bp from the TSSs) (Table S6). BS-PCR primers for porcine satellite DNA was from a previous study (Kang et al., 2001) (Table S6). We sequenced 10-16 clones from three independent samples. DNA methylation levels were evaluated as the ratio of methylated CpG number to the total CpG number by BiQ Analyzer (v2.02).

Supplemental References

Deng, Q., Ramskold, D., Reinius, B., and Sandberg, R. (2014). Single-cell RNA-seq reveals dynamic, random monoallelic gene expression in mammalian cells. Science *343*, 193-196.

Gao, X., Nowak-Imialek, M., Chen, X., Chen, D., Herrmann, D., Ruan, D., Chen, A.C.H., Eckersley-Maslin, M.A., Ahmad, S., Lee, Y.L., et al. (2019). Establishment of porcine and human expanded potential stem cells. Nat. Cell Biol. *21*, 687-699.

Graf, A., Krebs, S., Zakhartchenko, V., Schwalb, B., Blum, H., and Wolf, E. (2014). Fine mapping of genome activation in bovine embryos by RNA sequencing. Proc. Natl. Acad. Sci. U S A *111*, 4139-4144.

He, X., Tan, C., Li, Z., Zhao, C., Shi, J., Zhou, R., Wang, X., Jiang, G., Cai, G., Liu, D., et al. (2019). Characterization and comparative analyses of transcriptomes of cloned and in vivo fertilized porcine pre-implantation embryos. Biol. Open *8*, bio039917.

Hendrickson, P.G., Dorais, J.A., Grow, E.J., Whiddon, J.L., Lim, J.W., Wike, C.L., Weaver, B.D., Pflueger, C., Emery, B.R., Wilcox, A.L., et al. (2017). Conserved roles of mouse DUX and human DUX4 in activating cleavage-stage genes and MERVL/HERVL retrotransposons. Nat. Genet. *49*, 925-934.

Jiang, Z., Sun, J., Dong, H., Luo, O., Zheng, X., Obergfell, C., Tang, Y., Bi, J., O'Neill, R., Ruan, Y., et al. (2014). Transcriptional profiles of bovine in vivo pre-implantation development. BMC Genomics *15*, 756.

Kang, Y.K., Koo, D.B., Park, J.S., Choi, Y.H., Kim, H.N., Chang, W.K., Lee, K.K., and Han, Y.M. (2001). Typical demethylation events in cloned pig embryos. Clues on species-specific differences in epigenetic reprogramming of a cloned donor genome. J. Biol. Chem. *276*, 39980-39984.

Kong, Q., Yang, X., Zhang, H., Liu, S., Zhao, J., Zhang, J., Weng, X., Jin, J., and Liu, Z. (2020). Lineage specification and pluripotency revealed by transcriptome analysis from oocyte to blastocyst in pig. FASEB J. *34*, 691-705.

Kuijk, E.W., du Puy, L., van Tol, H.T., Haagsman, H.P., Colenbrander, B., and Roelen, B.A. (2007). Validation of reference genes for quantitative RT-PCR studies in porcine oocytes and preimplantation embryos. BMC Dev. Biol. *7*, 58.

Liu, W., Liu, X., Wang, C., Gao, Y., Gao, R., Kou, X., Zhao, Y., Li, J., Wu, Y., Xiu, W., et al. (2016). Identification

of key factors conquering developmental arrest of somatic cell cloned embryos by combining embryo biopsy and single-cell sequencing. Cell Discov. *2*, 16010.

Liu, X., Hao, Y., Li, Z., Zhou, J., Zhu, H., Bu, G., Liu, Z., Hou, X., Zhang, X., and Miao, Y.L. (2020). Maternal Cytokines CXCL12, VEGFA, and WNT5A Promote Porcine Oocyte Maturation via MAPK Activation and Canonical WNT Inhibition. Front. Cell Dev. Biol. *8*, 578.

Yan, L., Yang, M., Guo, H., Yang, L., Wu, J., Li, R., Liu, P., Lian, Y., Zheng, X., Yan, J., et al. (2013). Single-cell RNA-Seq profiling of human preimplantation embryos and embryonic stem cells. Nat. Struct. Mol. Biol. *20*, 1131-1139.

Zhang, X., Xue, B., Li, Y., Wei, R., Yu, Z., Jin, J., Zhang, Y., and Liu, Z. (2019). A novel chemically defined serum- and feeder-free medium for undifferentiated growth of porcine pluripotent stem cells. J. Cell Physiol. *234*, 15380-15394.
