# Supplementary material for: TDG is a pig-specific epigenetic regulator with insensitivity to H3K9 and H3K27 demethylation in nuclear transfer embryos
Source: Stem Cell Reports. 2021 Oct 21;16(11):2674–89. doi: 10.1016/j.stemcr.2021.09.012 (PMC8581057; doi:10.1016/j.stemcr.2021.09.012)
Supplement: Document S2. Article plus supplemental information [file mmc6.pdf]

Stem Cell Reports
Article
OPEN ACCESS

TDG is a pig-specific epigenetic regulator with insensitivity to H3K9 and H3K27 demethylation in nuclear transfer embryos

Xin Liu,[1,2,6] Lu Chen,[3,6] Tao Wang,[1,2,6] Jilong Zhou,[1,2] Zhekun Li,[1,2] Guowei Bu,[1,2] Jingjing Zhang,[1,2] Shuyuan Yin,[1,2] Danya Wu,[1,2] Chengli Dou,[1,2] Tian Xu,[1,2] Hainan He,[1,2] Wei Zhu,[1,2] Longtao Yu,[1,2] Zhiting Liu,[1,2] Xia Zhang,[1,4] Zhen-Xia Chen,[3,*] and Yi-Liang Miao[1,2,4,5,*]

[1]Institute of Stem Cell and Regenerative Biology, College of Animal Science and Veterinary Medicine, Huazhong Agricultural University, Wuhan 430070, China
[2]Key Laboratory of Agricultural Animal Genetics, Breeding and Reproduction (Huazhong Agricultural University), Ministry of Education, Wuhan 430070, China
[3]Hubei Key Laboratory of Agricultural Bioinformatics, College of Life Science and Technology, Huazhong Agricultural University, Wuhan 430070, China
[4]The Cooperative Innovation Center for Sustainable Pig Production, Wuhan 430070, China
[5]Hubei Hongshan Laboratory, Wuhan 430070, China
[6]These authors contributed equally
*Correspondence: zhen-xia.chen@mail.hzau.edu.cn (Z.-X.C.), miaoyl@mail.hzau.edu.cn (Y.-L.M.)
https://doi.org/10.1016/j.stemcr.2021.09.012

SUMMARY

Pig cloning by somatic cell nuclear transfer (SCNT) frequently undergoes incomplete epigenetic remodeling during the maternal-to-zygotic transition, which leads to a significant embryonic loss before implantation. Here, we generated the first genome-wide landscapes of histone methylation in pig SCNT embryos. Excessive H3K9me3 and H3K27me3, but not H3K4me3, were observed in the genomic regions with unfaithful embryonic genome activation and donor-cell-specific gene silencing. A combination of H3K9 demethylase KDM4A and GSK126, an inhibitor of H3K27me3 writer, were able to remove these epigenetic barriers and restore the global transcriptome in SCNT embryos. More importantly, thymine DNA glycosylase (TDG) was defined as a pig-specific epigenetic regulator for nuclear reprogramming, which was not reactivated by H3K9me3 and H3K27me3 removal. Both combined treatment and transient *TDG* overexpression promoted DNA demethylation and enhanced the blastocyst-forming rates of SCNT embryos, thus offering valuable methods to increase the cloning efficiency of genome-edited pigs for agricultural and biomedical purposes.

INTRODUCTION

Pig cloning by somatic cell nuclear transfer (SCNT) holds great promise for agriculture and biomedicine and has been widely employed to produce genome-edited pigs for breed improvement (Xu et al., 2020a; Zheng et al., 2017), human disease research (Yan et al., 2018; Zhang et al., 2018), and organ donation (Niu et al., 2017; Yue et al., 2020). For example, by deleting *CD163* and *pAPN* in donor cells, cloned pigs exhibited resistance to porcine viruses such as PRRSV, TGEV, and PDCoV (Xu et al., 2020a). In addition, PERVKO·3KO·9TG recloned pigs were created for safe and efficient xenotransplantation, which carries 3KO to eliminate xenoantigens, 9TG to enhance immunological compatibility to humans, and PERVKO to prevent viral transmission (Yue et al., 2020). However, despite tremendous efforts over the last two decades, such as modulating embryonic epigenetic modifications (Zhao et al., 2010) and knocking out *XIST* in donor cells (Ruan et al., 2018), the birth of full-term cloned pigs remains inefficient (< 3%). Notably, SCNT embryos still exhibit a developmental block during the maternal-to-zygotic transition (MZT), which is considered the consequence of incomplete epigenetic reprogramming in somatic genomes.

In mouse cloning, this incomplete reprogramming has been successfully rectified. Histone 3 lysine 9 trimethylation (H3K9me3) was the first confirmed epigenetic barrier to prevent MZT. Overexpression with H3K9 demethylases in SCNT embryos, such as lysine-specific demethylase 4D (KDM4D) and KDM4B, was able to improve embryonic genome activation (EGA) and the blastocyst rate (~90%) (Liu et al., 2016; Matoba et al., 2014). Another repressive mark, H3K27me3, turned out to be more complicated. Injection of either *KDM6A* mRNA or *KDM6B* small interfering RNA reduced H3K27me3 enrichments in two-cell embryos and promoted blastocyst formation (Yang et al., 2018). A previous report also demonstrated that H3K4me3 impeded *Xenopus* nuclear reprogramming by maintaining the transcriptional memory of somatic cells (Hormanseder et al., 2017). However, anomalous H3K4me3 might not lead to the EGA arrest of mouse SCNT embryos (Liu et al., 2016). Before histone methylation resetting, DNA hypermethylation was identified in mouse cloning without an effective rescue approach (Tsuji et al., 2009; Yang et al., 2007). Recently, knocking down of DNA methyltransferase was determined to improve mouse cloned EGA and the blastocyst rate, and this rate was even higher (> 95%) when combined with histone demethylase overexpression (Gao et al., 2018). All these studies inspired us to investigate epigenetic barriers and defective factors during pig cloned MZT, which previously have not been comprehensively elucidated.

 © 2021 The Author(s).
This is an open access article under the CC BY license (http://creativecommons.org/licenses/by/4.0/).

In the present study, we combined RNA sequencing (RNA-seq), ultra-low-input native chromatin immunoprecipitation sequencing (ULI-NChIP-seq), and whole-genome bisulfite sequencing (WGBS) to investigate histone modification and DNA methylation on the donor genome in pig SCNT embryos. More importantly, insufficient activation of thymine DNA glycosylase (TDG), a DNA demethylation regulator specifically expressed during the pig MZT, was defined as a novel barrier for nuclear reprogramming. Taken together, our study reveals a unique interplay between histone modification and DNA methylation in pig nuclear reprogramming and provides effective methods to overcome multiple epigenetic barriers in this process.

RESULTS

Identification of reprogramming-resistant genes and regions in pig SCNT embryos

Large-scale analyses of the embryonic transcriptomes have reported different MZT timing between *in vivo*-fertilized (IVO) embryos (four-cell stage) and SCNT embryos (eight-cell stage) (Cao et al., 2014a; He et al., 2019). Here, we investigated the RNA-seq profiles of two- to eight-cell IVO embryos (IVO2c, IVO4c, IVO8c), four- to eight-cell SCNT embryos (SCNT4c, SCNT8c), and the donor pig fetal fibroblasts (PFF) (Table S1). Both hierarchical clustering and principal component analysis (PCA) (Figures S1A and S1B) indicated that the transcriptional patterns of SCNT8c, but not SCNT4c, were close to IVO4c, reconfirming the MZT delay in SCNT embryos. Nevertheless, 93.4% of SCNT embryos displayed a developmental block at the four-cell stage when treated with transcriptional inhibitor α-amanitin (Table S2), indicating that the newly synthesized RNA of SCNT4c is critical for the subsequent cleavage. Thus, we decided to analyze the transcriptional difference between IVO and SCNT embryos at the same four-cell stage.

By comparative analysis, 3,070 differentially expressed genes (DEGs) (fold change [FC] > 3, fragments per kilobase of exon per million mapped fragments [FPKM] > 5 in IVO4c, $p < 0.05$) were highly expressed in IVO4c compared with IVO2c, termed EGA-ON genes (Figure S1C and Table S3). Among these, 734 and 1,106 genes were fully (FC $\leq$ 2, IVO4c versus SCNT4c) and partially (2 < FC $\leq$ 5) activated in SCNT4c, while 1,230 genes failed to be activated (EGA-OFF; FC > 5). Gene ontology (GO) analysis suggested that SCNT4c displayed competence for maternal mRNA decay and the defects for activating genes enriched in DNA-dependent transcription (Figure S1D). Next, 4,947 downregulated DEGs were identified in IVO4c compared with PFF (FC > 3, FPKM > 5 in PFF, $p < 0.05$), termed PFF-OFF genes (Figure S1E and Table S3). Most genes were fully (3,678; FC $\geq$ 5, PFF versus SCNT4c) and partially (740; 2 $\leq$ FC < 5) decreased in SCNT4c, which are implicated in the Wnt signaling pathway, cell junction, and epithelium morphogenesis. Only 529 genes (PFF-ON; FC < 2) were still highly expressed during cloned MZT, and were partially involved in DNA damage response and protein ubiquitination (Figure S1F).

To comprehensively dissect the difference of protein-coding and non-coding transcripts, we also performed a sliding window strategy as previously reported (Chung et al., 2015; Matoba et al., 2014). Compared with IVO2c, IVO4c harbored 2,704 EGA-ON genomic regions (FC > 5, reads of exon per million mapped reads [RPM] > 10 in IVO4c, $p < 0.01$) (Figure 1A and Table S4). Among these, 708 regions were fully activated (FC $\leq$ 2, IVO4c versus SCNT4c), 1,251 regions were partially activated (2 < FC $\leq$ 5), and 745 regions were deficiently activated in SCNT4c (EGA-OFF; FC > 5). We also identified 3,380 PFF-OFF regions that were silenced in IVO4c compared with PFF (FC > 5, RPM > 10 in PFF, $p < 0.01$) (Figure 1B and Table S4). Among these, 589 regions kept their expression levels in SCNT4c as those in PFF (FC < 2, PFF versus SCNT4c), termed PFF-ON regions. Meanwhile, 1,721 and 1,070 regions were fully (FC $\geq$ 5) or partially (2 $\leq$ FC < 5) restored to the transcriptional quiescence state in SCNT4c. Here, all these EGA-OFF/PFF-ON genes and regions are termed "reprogramming-resistant genes and regions" in SCNT4c.

EGA-OFF regions are enriched for H3K9me3 and H3K27me3 in SCNT embryos

Having ascertained reprogramming-resistant genes and regions in SCNT4c, we then asked whether their anomalous expression patterns are stabilized by epigenetic modifications from donor cells. Therefore, we performed ULI-NChIP-seq on PFF and SCNT4c with 200 cells, and generated the genome-wide maps of H3K4me3, H3K9me3 and H3K27me3 (Table S1). Strikingly, compared with the chromatin immunoprecipitation sequencing (ChIP-seq) using 10e7 PFF (Gao et al., 2019), over 70% of H3K4me3 peaks were detected in our low-input ChIP-seq data (Figures S2A and S2B). The correlation coefficients between two replicates ranged from 0.85 to 0.98 (Figure S2C), indicating the high reproducibility of this procedure.

We first assessed the histone methylation on EGA-ON/OFF regions. As shown in Figure 1C, the ChIP-seq intensities of all three marks in PFF were obviously higher on EGA-OFF regions compared with those on EGA-ON regions. Notably, EGA-OFF regions still preserved H3K9 and H3K27 hypermethylation in SCNT4c, suggesting that these two modifications are pre-existing epigenetic barriers for cloned EGA. A representative region shows the H3K9me3 and H3K27me3 depositions in PFF and SCNT4c with unfaithful gene activation in SCNT4c (Figure 1D). Simultaneously, EGA-OFF regions were deficiently

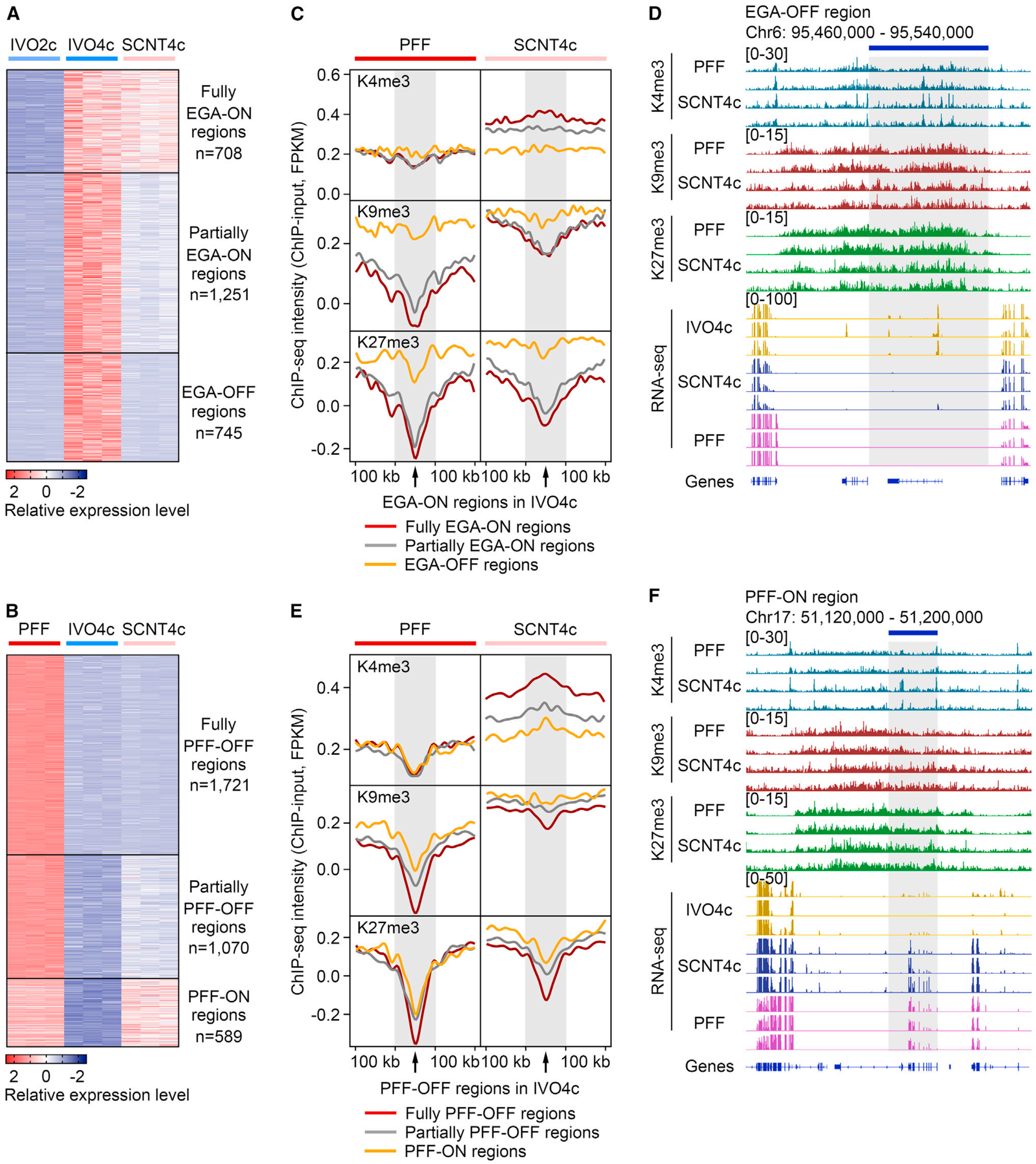

Figure 1. Reprogramming-resistant regions are enriched for H3K9me3 and H3K27me3

(A and B) Heatmaps showing the expression levels of EGA-ON regions (A) and PFF-OFF regions (B) in IVO4c. Each row represents the normalized RPM of a region and each column represents a replicate. EGA-ON regions, ranging from 50 to 575 kb, are classified into three groups based on the fold change (FC) in expression differences between IVO4c and SCNT4c (Fully EGA-ON, FC $\leq$ 2; Partially EGA-ON, 2 < FC $\leq$ 5; EGA-OFF, FC > 5). PFF-OFF regions, ranging from 50 to 560 kb, are classified into three groups based on their expression differences between PFF and SCNT4c (Fully PFF-OFF, FC $\geq$ 5; Partially PFF-OFF, 2 $\leq$ FC < 5; PFF-ON, FC < 2).

(legend continued on next page)

enriched for H3K4me3 in SCNT4c (Figure 1C), suggesting that H3K4 hypomethylation is also responsible for EGA failure. We next determined the chromatin structures of EGA-ON/OFF regions by analyzing the distributions of protein-coding genes and repetitive sequences. As expected, EGA-OFF regions contained relatively few genes compared with EGA-ON regions, and they were enriched for repetitive sequences such as LINE and LTR (Figures S2D and S2E). These findings indicate that EGA-OFF regions are generally located in heterochromatin regions, which prevents transcriptional activation of the donor genome.

PFF-ON genes and regions are not involved in excessive H3K4me3 in SCNT embryos

By further assessing the histone methylation on PFF-ON/OFF regions, no difference in H3K4me3 was detected among these regions in PFF, and its enrichments were even lower on PFF-ON regions in SCNT4c (Figure 1E). Moreover, except for H3K27me3 in PFF between partially PFF-OFF and PFF-ON regions, H3K9me3 and H3K27me3 levels in PFF and SCNT4c were significantly higher on PFF-ON regions than those on PFF-OFF regions (Figure 1E), an example of which is shown in Figure 1F. Since H3K9me3 and H3K27me3 are associated with gene repression (Dambacher et al., 2010), we thus wondered whether transcriptional activation still occurred in these H3K9me3/H3K27me3-marked regions. Perhaps PFF-ON regions also possess heterochromatin features to maintain the leaky expression from PFF (Saksouk et al., 2015; Vanrobays et al., 2017), because we confirmed that PFF-ON regions were also relatively gene-poor regions enriched with LINE and LTR (Figures S2D and S2E).

Recently, donor-cell-specific genes expressed in *Xenopus* SCNT embryos have been reported to exhibit H3K4me3 hypermethylation on their transcription start sites (TSSs) (Hormanseder et al., 2017). However, PFF-ON genes in pigs exhibited the lowest H3K4me3 enrichments around their TSSs (±5 kb) in PFF and SCNT4c when compared with PFF-OFF genes, while EGA-OFF genes only exhibited relatively low H3K4me3 enrichments in PFF when compared with fully EGA-ON genes (Figures 2A–2D). Meanwhile, high levels of H3K9me3 and H3K27me3 were observed around the TSSs of EGA-OFF/PFF-ON genes (Figures 2A–2D), consistent with the results of EGA-OFF/PFF-ON regions. Taking these data together, we propose that PFF transcriptional profiles preserved in SCNT4c are stabilized by H3K9me3 and H3K27me3, but not H3K4me3.

KDM4A overexpression and GSK126 incubation improve SCNT developmental potential

To overcome aberrant H3K9 and H3K27 methylation on EGA-OFF/PFF-ON regions, we injected 1,000 ng/μL mRNA encoding the pig histone demethylases, KDM4A and KDM6A, into one-cell SCNT embryos (Figure S3A and Table S6). Global histone modifications were then determined by immunofluorescence in SCNT4c (Figures S3B and S3C). In the control group, 100% and 65.2% of embryos were positively stained with H3K9me3 and H3K27me3, respectively. These percentages were decreased to ~5%–10% when embryos were injected with *KDM4A* and *KDM6A* individually or in combination (Figure S3C). Nonetheless, compared with the controls (16.6%), *KDM6A* injection showed no improvement on blastocyst formation (19.5%), and even impaired the beneficial effect of *KDM4A* (27.5%) when both mRNAs were injected (15.3%) (Figures S3D and S3E; Table S2). Thus, we decided to find another approach to reduce H3K27me3.

Suppression of EZH2 activity, the methyltransferase responsible for establishing H3K27me3, also contributed to nuclear reprogramming (Xie et al., 2016; Zhou et al., 2019). Hence, an EZH2-specific inhibitor, GSK126, was used during cloned MZT (Figure 3A). As shown in Figures 3B and 3C, the majority of embryos (85.8%) exhibited no H3K27me3 staining when they were treated with 0.1 μM GSK126 for 48 h. More importantly, comparable blastocyst rates were observed in the GSK126-treated (24.7%) and *KDM4A*-injected groups (26.8%), and this rate was even higher when combining these two methods (33.5%) (Figures 3D and 3E; Table S2). Blastocyst quality was then evaluated by SOX2 immunostaining and TUNEL assay. The results revealed a significant increase of total cell number and inner cell mass cell number in the experimental group, while the number of apoptotic cells showed no decline (Figures S3F and S3G). Furthermore, the experimental group also showed an increased outgrowth colony-forming number with alkaline phosphatase-positive staining (Figures 3F and 3G), suggesting its improved competence for post-implantation development.

Combination of *KDM4A* and GSK126 restores the transcriptome of SCNT embryos

To determine the effects of *KDM4A* + GSK126 treatment, we performed RNA-seq of combined-treated SCNT4c (SCNT4c-KG). The hierarchical clustering and PCA analysis results revealed that the transcriptional patterns of SCNT4c-KG were

(C and E) ChIP-seq intensity of H3K4me3, H3K9me3, and H3K27me3 are shown within EGA-ON/OFF (C) and PFF-ON/OFF (E) regions, and their 100-kb flanking regions. Read counts are normalized by input, total mapped reads, and region length.
(D and F) Genome browser view of H3K4me3, H3K9me3, and H3K27me3 enrichments and transcriptional levels in the representative EGA-OFF (D) and PFF-ON (F) regions.
See also Figure S2; Tables S1 and S4.

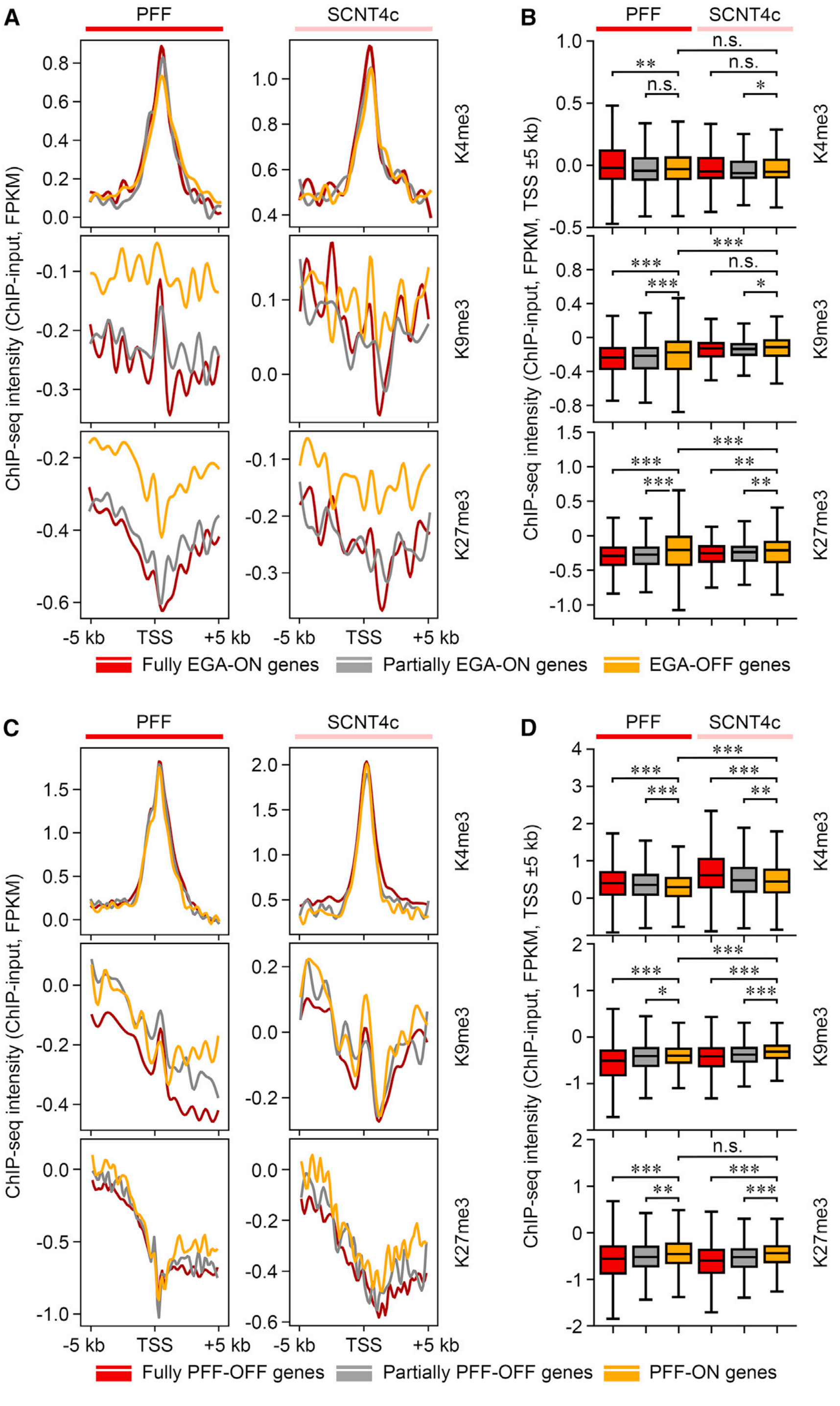

Figure 2. Reprogramming-resistant genes are enriched for H3K9me3 and H3K27me3 around their TSSs

(A and C) ChIP-seq intensity of H3K4me3, H3K9me3, and H3K27me3 are shown around the transcription start sites (TSSs; ±5 kb) of EGA-ON/OFF (A) and PFF-ON/OFF (C) genes. Read counts are normalized by input and total mapped reads.

(B and D) Box plots comparing the average intensity of H3K4me3, H3K9me3, and H3K27me3 around the TSSs (±5 kb) of EGA-ON/OFF (B) and PFF-ON/OFF (D) genes. *$p < 0.05$, **$p < 0.01$, ***$p < 0.001$; n.s., not significant; two-tailed Student's t test. Reads counts are normalized by input and total mapped reads.

See also Figure S1 and Table S3.

close to IVO4c, but different from SCNT4c (Figures 4A and 4B). By profiling the data, as much as 53.9% of the 1,230 EGA-OFF genes (FC > 2, SCNT4c-KG versus SCNT4c) and 58.2% of the 529 PFF-ON genes (FC < 0.5) were restored by *KDM4A* + GSK126 treatment, and it also upregulated 40.3% of the 745 EGA-OFF regions and downregulated 72.7% of the 589 PFF-ON regions in SCNT4c (Figures 4C–4F and S4A; Tables S3 and S4). Figure 4G displays the representative genome browser view of two candidate reprogramming-resistant regions containing several restored genes in

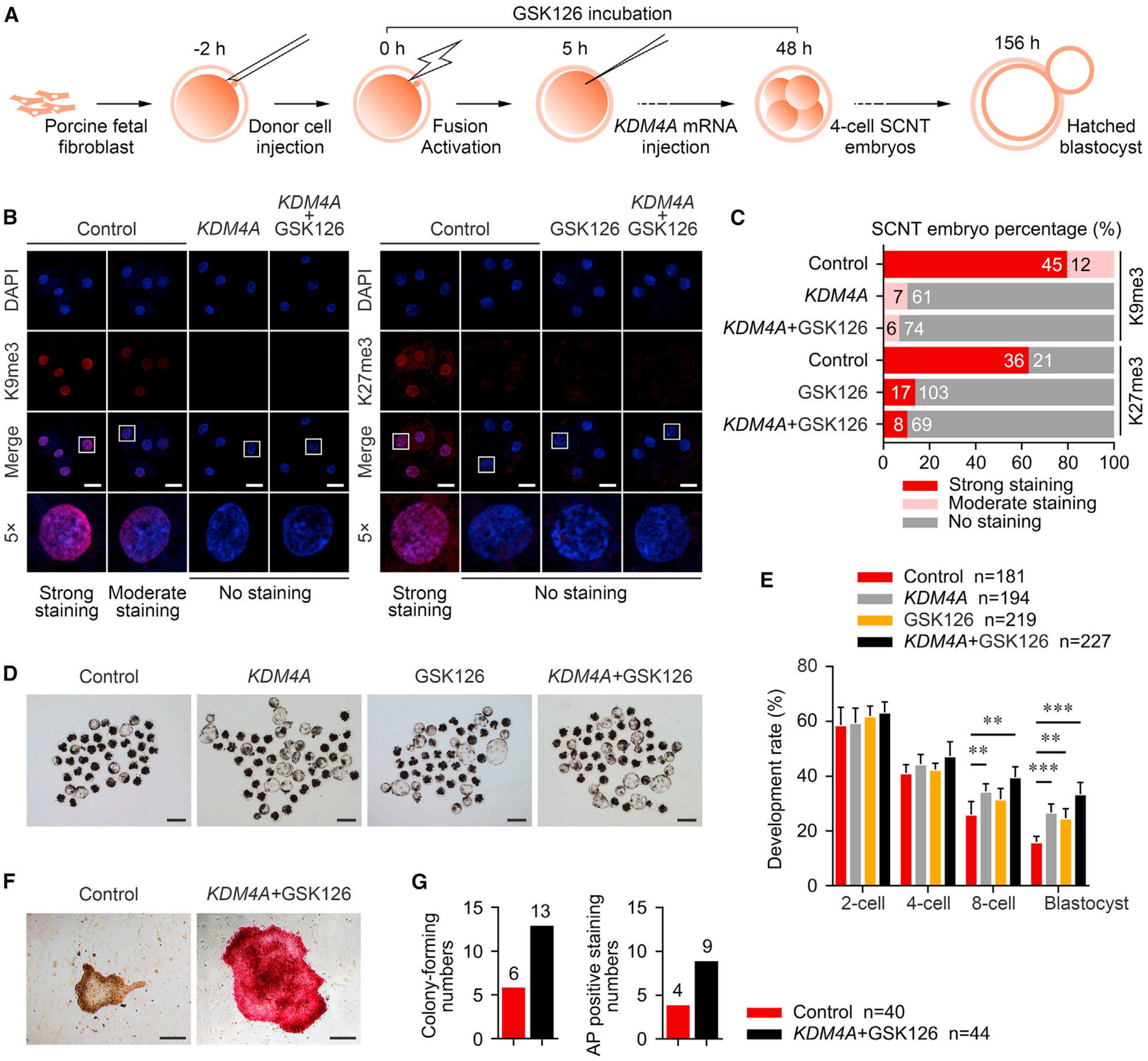

Figure 3. *KDM4A* + GSK126 treatment improves SCNT embryonic development

(A) Experimental design of the treatment procedure. SCNT embryos were injected with *KDM4A* mRNA at 5 h after activation, or treated by GSK126 at 0–48 h.

(B) Immunostaining of H3K9me3 and H3K27me3 (red) and DNA (blue) in SCNT4c derived from 1,000 ng/μL *KDM4A*-injected, 0.1 μM GSK126-treated, combined-treated, and non-treated groups. One of the nuclei in SCNT4c is magnified 5-fold. Scale bars, 50 μm.

(C) Bar plot showing the percentage of SCNT4c with positive H3K9me3 and H3K27me3 staining and no staining in different groups. Numbers of the total embryos analyzed from 3 to 5 replicates are shown in the bars.

(D) Representative images of different groups after culturing for 6.5 days *in vitro*. Scale bars, 200 μm.

(E) Bar plot showing the development rates in different groups. Error bars represent the SD. Numbers of the total embryos analyzed from five replicates are shown in the legend. $^{**}p < 0.01$, $^{***}p < 0.001$; two-tailed Student's t test.

(F) Representative images of outgrowth colonies in combined-treated group with alkaline phosphatase (AP)-positive staining and non-treated group with AP-negative staining. Scale bars, 200 μm.

(G) Bar plots comparing the colony-forming and AP-positive staining numbers in combined-treated and non-treated groups. Numbers of the total blastocysts analyzed from five replicates are shown in the legend.

See also Figure S3 and Table S2.

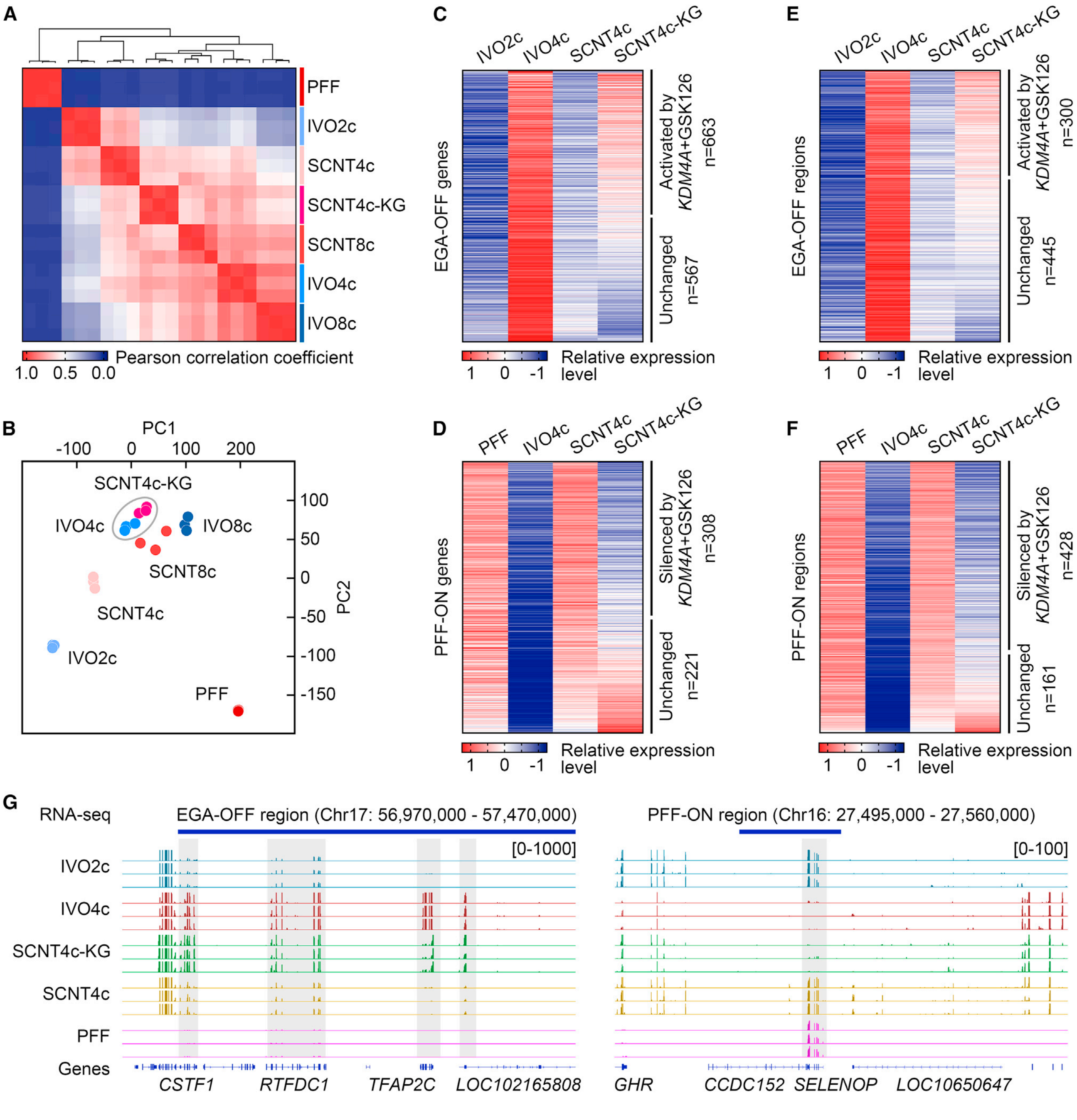

Figure 4. *KDM4A* + GSK126 treatment facilitates EGA initiation and somatic cell memory silencing

(A) Heatmap comparing the Pearson correlation coefficients of PFF, IVO, and SCNT embryos at different stages, and SCNT4c derived from combined *KDM4A* + GSK126 treatment group (SCNT4c-KG). Hierarchical clustering is shown in the top panel.

(B) Principal component analysis of expression patterns among different samples.

(C and D) Heatmap showing the expression levels of EGA-OFF genes (Figure S1C) among IVO2c, IVO4c, SCNT4c, and SCNT4c-KG (C), and PFF-ON genes (Figure S1E) among PFF, IVO4c, SCNT4c, and SCNT4c-KG (D). Each row represents the normalized FPKM of a transcript. FC > 2 are EGA-OFF genes reactivated in SCNT4c-KG (SCNT4c-KG versus SCNT4c), FC < 0.5 are PFF-ON genes silenced in SCNT4c-KG.

(legend continued on next page)

SCNT4c-KG. We also analyzed the expression of six EGA-OFF genes (Figures S5A and S5B) via qPCR, and found that *KDM4A* + GSK126 treatment successfully upregulated *KLF17*, *PNRC1*, *SUPT4H1*, *TFAP2C*, and *ZSCAN4* (Figure S5C and Table S6), partially confirming our RNA-seq results (Figure S5B).

In addition to protein-coding genes, repeatome also exhibits a shift during the MZT. Previous studies have identified several repetitive elements that possess EGA-ON profiles in mouse (MERVL and GSAT), human (HERVL and HSATII), and cattle (MLT1A0 and ERV1-2-I_BT) embryos (Halstead et al., 2020; Hendrickson et al., 2017). Moreover, the repression of satellite DNA and MERVL in mouse SCNT embryos was relieved by *KDM4D* overexpression (Matoba et al., 2014). In the pig, we identified 4,338 repeat sequences that were highly expressed in IVO4c compared with IVO2c (FC > 1, variance stabilizing transformation [VST] count > 5 in IVO4c) (Figures 5A and 5B; Table S5). Strikingly, these repeats were poorly activated in SCNT4c, and *KDM4A* + GSK126 treatment improved their transcriptions (Figure 5A). In detail, eight types of repetitive elements were successfully reactivated, including LTR and satellite, while LINE and DNA transposons were insensitive to the combined treatment (Figure S4B and Table S5). In particular, we defined SSRS1 (satellite), ERV1-2-I_SS (ERV1), ERV1-2B-LTR_SS (ERV1), MLT1E2 (ERVL), and LTR14B_SS (ERVK) as EGA indicators for pig IVO and SCNT embryos during the MZT (Figures 5C and 5D).

Unfaithful activation of *TDG* results in abnormal DNA demethylation in SCNT embryos

Among the EGA-OFF genes, we surprisingly found that the deficient *TDG* expression could not be rescued by *KDM4A* + GSK126 treatment (Figure 6A). Notably, *TDG* is specifically transcribed during pig MZT (He et al., 2019; Kong et al., 2020) and is barely detected at the MZT stage in mouse (Deng et al., 2014; Liu et al., 2016), human (Hendrickson et al., 2017; Yan et al., 2013), and cattle embryos (Graf et al., 2014; Jiang et al., 2014) (Figures 6B and S4C). TDG is a well-known enzyme for excising thymine, 5-hydroxymethyluracil (5hmU), 5-formylcytosine (5fC), and 5-carboxylcytosine (5caC), which are generated from either 5-methylcytosine (5mC) deamination by AICDA/APOBECs or 5mC hydroxylation by TET proteins (Figure 6C) (Cortellino et al., 2011; Maiti and Drohat, 2011). Accordingly, we surmise that insufficient activation of *TDG* may lead to an abnormal DNA demethylation in pig SCNT4c.

We first determined that *TDG* mRNA injection before MZT worsened SCNT blastocyst formation in a dose-dependent manner (Tables S2 and S6). A doxycycline-inducible donor cell line was then used to achieve transient *TDG* expression during cloned MZT (Figure 6D). By immunostaining, when compared with the controls (5mC, 79.7%; 5-hydroxymethylcytosine [5hmC], 82.6%), SCNT4c-KG exhibited loss of 5mC (48.8%) and 5hmC (64.4%), and these percentages (5mC, 35.5%; 5hmC, 44.4%) were much lower in SCNT4c with *TDG* overexpression (SCNT4c-TDG; Figures 6E and 6F). SCNT4c-KG also showed an obvious 5caC accumulation (51.9%), perhaps due to the upstream DNA demethylation process but still lacking *TDG* expression. By contrast, SCNT4c-TDG showed a small proportion of positive 5caC staining (8.9%) (Figures 6E and 6F). Excitingly, qPCR results revealed that transient *TDG* expression could reactivate several candidate EGA-OFF genes (Figure S5C and Table S6) and attenuate the developmental defects of SCNT embryos with an ~10% elevation in blastocyst rate (Figures 6G and 6H; Table S2).

To precisely analyze the DNA methylation changes among PFF, SCNT4c, and SCNT4c-TDG, we performed post-bisulfite adapter tagging sequencing (PBAT-seq), an ultra-low-input WGBS method, to generate their DNA methylation maps on a genome-wide scale. First, when compared with EGA-ON/PFF-OFF regions, DNA methylation levels were higher in EGA-OFF/PFF-ON regions in SCNT4c (Figure 7A). This confirms that DNA methylation exhibits the same reprogramming-resistant feature as H3K9me3 and H3K27me3 in SCNT4c. Second, the DNA methylation levels of EGA-OFF/PFF-ON regions in SCNT4c-TDG were significantly lower than those in PFF and SCNT4c, indicating an effective DNA demethylation achieved by transient *TDG* expression (Figure 7B). DNA demethylation by TDG could also be observed at EGA-OFF/PFF-ON gene promoters, no matter whether these promoters contained a CpG island or not (Figure 7C). Third, as examples, the DNA methylation levels of satellite DNA, candidate EGA-OFF gene promoters (*TFAP2C*, *SUPT4H1*, *KLF17*), and PFF-ON gene promoters (*UBC*, *USP5*, *TRIM8*) were examined by bisulfite sequencing PCR (Table S6), and their downtrends of DNA methylation were correlated with their transcriptional restorations in SCNT4c-TDG (Figures S5C and S6A–S6C; Tables S3 and S5). Fourth, we determined the transcriptional profile of SCNT4c-TDG, which was also close to IVO4c, like SCNT4c-KG (Figure 7D). Moreover, we found that most

(E and F) Heatmap showing the expression levels of EGA-OFF regions (Figure 1A) among IVO2c, IVO4c, SCNT4c, and SCNT4c-KG (E), and PFF-ON regions (Figure 1B) among PFF, IVO4c, SCNT4c, and SCNT4c-KG (F). Each row represents the normalized RPM of a region. FC > 2 are EGA-OFF regions reactivated in SCNT4c-KG (SCNT4c-KG versus SCNT4c), FC < 0.5 are PFF-ON regions silenced in SCNT4c-KG.
(G) Genome browser view of transcriptional levels in representative EGA-OFF/PFF-ON regions.
See also Figures S4 and S5; Tables S3 and S4.

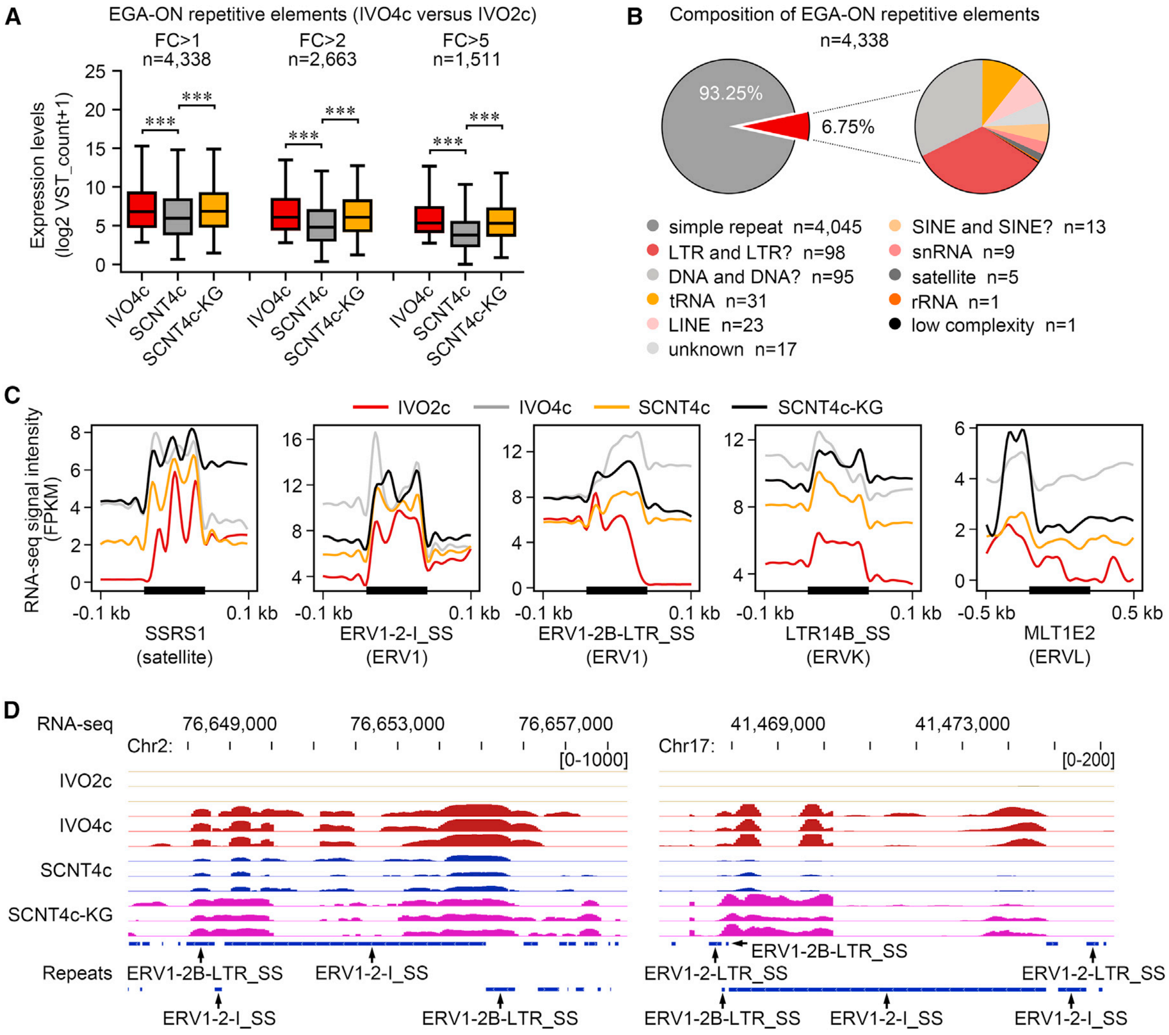

Figure 5. *KDM4A* + GSK126 treatment reactivates repetitive element transcription
(A) Box plots comparing the normalized VST count of EGA-ON repetitive elements among IVO4c, SCNT4c, and SCNT4c-KG. Repetitive elements are shown in three groups based on their expression differences between IVO2c and IVO4c (FC > 1, 2, 5). ***p < 0.001; two-tailed Student's t test.
(B) The composition of 4,338 EGA-ON repetitive elements.
(C) RNA read enrichments (FPKM) around candidate repetitive elements among IVO2c, IVO4c, SCNT4c, and SCNT4c-KG.
(D) Genome browser view of transcriptional levels in two genomic regions containing ERV1-2-I_SS and ERV1-2B-LTR_SS.
See also Figure S4 and Table S5.

restored genes and regions were shared in SCNT4c-KG and SCNT4c-TDG (Figures S7A–S7E; Tables S3 and S4), suggesting that the detailed gene network regulated by H3K9me3, H3K27me3, and DNA methylation are highly similar during pig cloned EGA.

DISCUSSION

In fertilized embryos, histone methylation and DNA methylation undergo a widespread decrease at the pre-implantation stage in mouse (Gao et al., 2018; Liu et al., 2004; Xu et al., 2020b), human (Xu et al., 2020b; Zhu et al., 2018), cattle (Liu et al., 2018; Zhou et al., 2019, 2020), and pig (Cao et al., 2014b, 2015). By contrast, SCNT embryos frequently exhibit abnormal remodeling of these modifications on the donor genome, which is believed to be the primary reason for the significant embryonic losses during cleavage. In this study, we demonstrated that H3K9me3 and H3K27me3, but not H3K4me3, were epigenetic barriers to prevent EGA initiation and somatic cell memory silencing during pig cloned MZT. Moreover, DNA

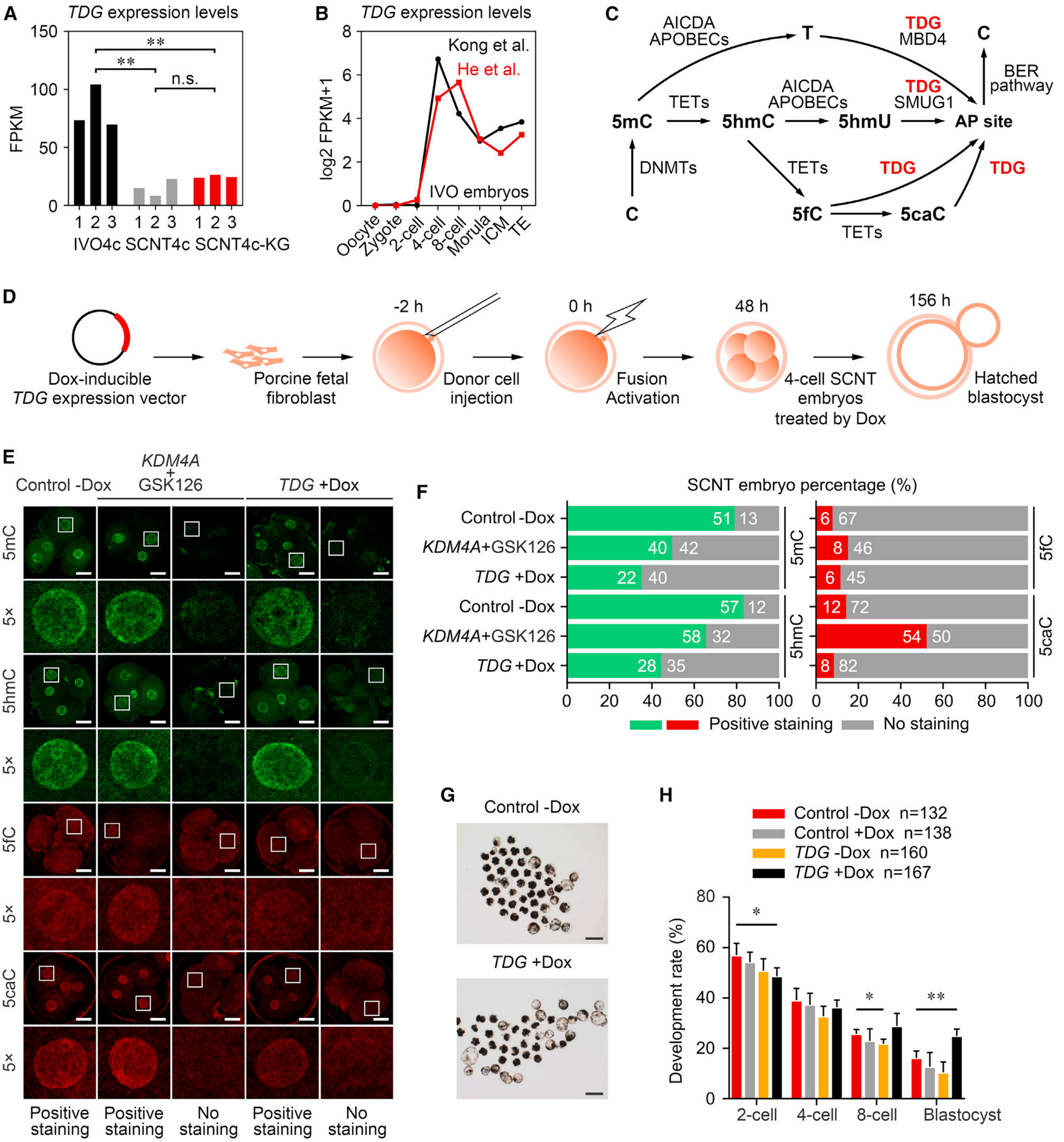

Figure 6. Transient *TDG* expression improves SCNT embryonic development

(A) Bar plots showing the FPKM of *TDG* in the RNA-seq data of IVO4c, SCNT4c, and SCNT4c-KG. **$p < 0.01$; n.s., not significant; two-tailed Student's t test.

(B) Line plots illustrating the dynamic transcriptional changes of *TDG* in pig IVO embryos (He et al., 2019; Kong et al., 2020).

(C) Schematic illustration of regulators related to the DNA methylation (C→5mC) and demethylation (5mC→C) process. AP site, abasic site; BER, base excision repair.

(D) Experimental design of the treatment procedure. SCNT embryos were reconstructed using PFF transfected with *TDG*-inducible expression vector, and induced to transiently express *TDG* by doxycycline (*TDG* + Dox) at 40–72 h after activation.

(legend continued on next page)

methylation was reduced along with H3K9me3 and H3K27me3 removal, whereas DNA demethylation could also be achieved by overexpressing a pig-specific epigenetic regulator, TDG (Figure 7E).

H3K9me3 and H3K27me3 have been verified as conserved reprogramming barriers among different species. In pig cloning, aberrant H3K9me3 and H3K27me3 have been observed at the one- to two-cell stage via immunofluorescence, while their detrimental effects to MZT remain unknown (Ruan et al., 2018; Xie et al., 2016). By using ULI-NChIP-seq, we showed for the first time that both H3K9me3 and H3K27me3 still existed during the MZT. More precisely, they were deposited on the genomic regions with unfaithful transcriptional patterns, namely EGA-OFF and PFF-ON regions. By contrast, EGA-OFF regions in mouse and human SCNT embryos were only enriched for H3K9me3, but not H3K27me3, and the information on PFF-ON regions was not mentioned (Chung et al., 2015; Matoba et al., 2014). Additionally, most studies utilized the corresponding demethylases to overcome these barriers, such as KDM4A for H3K9me3 in mouse and human (Chung et al., 2015), and KDM6A for H3K27me3 in mouse and cattle (Yang et al., 2018; Zhou et al., 2019). Our results confirmed their demethylation functions in pig SCNT embryos, but the blastocyst rate was not elevated by KDM6A, regardless of mRNA concentrations (Table S2). Previous studies have individually utilized KDM4A and GSK126 to increase the pig SCNT blastocyst rate (Ruan et al., 2018; Xie et al., 2016). Here, we combined these two methods with a longer GSK126 treatment time (up to 48 h) to further facilitate blastocyst formation, which might be due to an obvious reduction in cloned MZT defect.

In addition to EGA failure, persistent expression of donor-cell-specific genes, namely PFF-ON genes, also derives from incomplete reprogramming. Currently, excessive H3K4me3 has been detected in cattle eight-cell SCNT embryos (Zhou et al., 2020) and on the TSSs of PFF-ON genes in *Xenopus* donor cells (Hormanseder et al., 2017). Here, we found that PFF-ON regions or the TSSs of PFF-ON genes in pig cloning were enriched for H3K9me3 and H3K27me3, but not H3K4me3. Remarkably, most PFF-ON genes and regions were silenced through H3K9me3 and H3K27me3 removal. We also noticed that PFF-ON genes in *Xenopus* and cattle SCNT embryos were involved in donor cell identity, such as endoderm- and fibroblast-specific genes (Hormanseder et al., 2017; Zhou et al., 2020). In contrast, only nine PFF-ON genes were fibroblast-specific genes when we compared our PFF-ON genes with a list of 563 marker genes for mouse fibroblasts (Table S3) (Kim et al., 2010). Therefore, although SCNT embryos harbor PFF-ON genes in many species, the composition of PFF-ON genes and how they maintain their transcriptions are likely different between pig and other species. Another difference between our work and previous studies is the effect of H3K4me3 removal for reprogramming. H3K4 demethylation has been proved to promote development in mouse, cattle, and *Xenopus* cloning (Hormanseder et al., 2017; Liu et al., 2016; Zhou et al., 2020). Accordingly, we injected *KDM5A* mRNA (Table S6) and tested its H3K4 demethylase function in four-cell embryos (Figures S7F and S7G). However, *KDM5A* overexpression could not rescue the EGA defect and the poor developmental phenotype of pig SCNT embryos (Figure S5C and Table S2).

Soon after the establishment of the SCNT technology, DNA hypermethylation was the first determined epigenetic barrier. Deficient 5mC and 5hmC demethylation has been found in pig and cattle SCNT embryos by immunostaining (Cao et al., 2014b; Zhou et al., 2020). Most studies utilized 5-aza-2-deoxycytidine (5AZA), a DNA methyltransferase inhibitor, to deplete 5mC. However, improvements could be observed in human and pig (Huan et al., 2013; Sun et al., 2012) but not in mouse and cow (Ding et al., 2008; Tsuji et al., 2009). In this study, we investigated the interplay between histone modification and DNA methylation, since *KDM4A* + GSK126 treatment decreased the numbers of embryos with 5mC- and 5hmC-positive staining. Of note, *DNMT1* and *DNMT3B* are downregulated by this treatment (Table S3), similar to the effect of 5AZA. Furthermore, TDG is likely to serve as a rate-limiting factor for DNA demethylation in pig nuclear reprogramming, because its expression not only removes 5caC but also removes the upstream 5mC and 5hmC. In this study, we also combined *KDM4A* + GSK126 treatment with transient *TDG* expression to verify whether this triple treatment could raise the efficiency of nuclear reprogramming. Unexpectedly, it showed no more improvement of cloned blastocyst formation (Table S2). We speculate that

(E) Immunostaining of 5mC (green), 5hmC (green), 5fC (red), and 5caC (red) in SCNT4c derived from *TDG* + Dox, combined-treated, and non-treated groups. One of the nuclei in SCNT4c is magnified 5-fold. Scale bars, 50 μm.
(F) Bar plots showing the percentage of SCNT4c derived from different groups with positive and no staining of DNA methylation. Numbers of the total embryos analyzed from 3 to 4 replicates are shown in the bars.
(G) Representative images of *TDG* + Dox and non-treated groups after culturing for 6.5 days *in vitro*. Scale bars, 200 μm.
(H) Bar plot showing the development rates in the *TDG* ± Dox, Dox-treated, and non-treated groups. Error bars represent the SD. Numbers of the total embryos analyzed from four replicates are shown in the legend. *$p < 0.05$, **$p < 0.01$; two-tailed Student's t test.
See also Figures S4–S7 and Table S2.

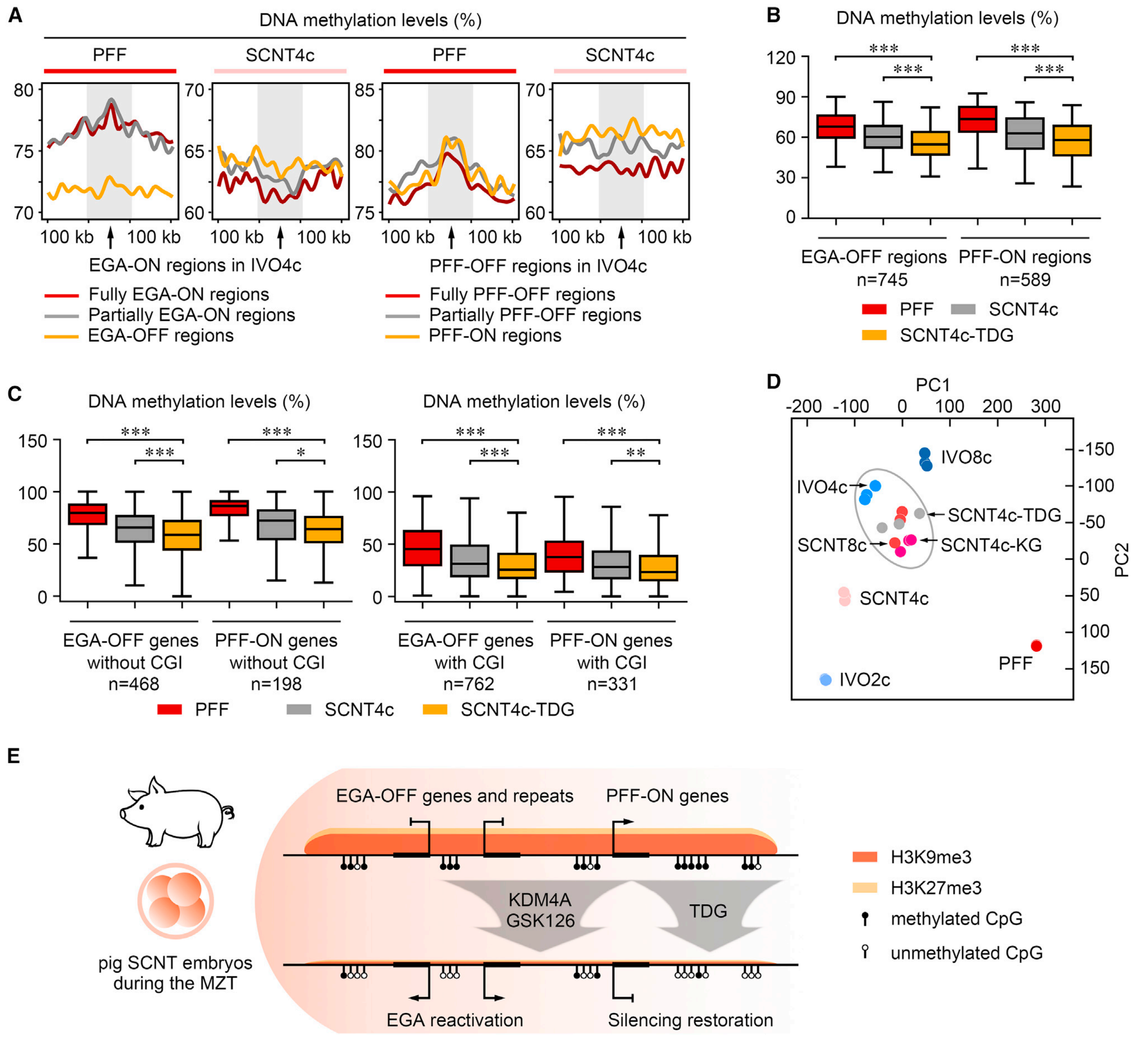

Figure 7. TDG regulates aberrant DNA methylation and transcription in SCNT embryos

(A) DNA methylation levels of EGA-ON/OFF and PFF-ON/OFF regions with their 100-kb flanking regions in PFF and SCNT4c. These levels are calculated as the average ratios of the methylated CpG to all CpG.

(B) DNA methylation levels of EGA-OFF and PFF-ON regions in PFF, SCNT4c, and SCNT4c overexpressed with *TDG* (SCNT4c-TDG). EGA-OFF regions are ranging from 50 to 575 kb and PFF-ON regions are ranging from 50 to 425 kb. These levels are calculated as the average ratios of the methylated CpG to all CpG. ***$p < 0.001$; two-tailed Student's t test.

(C) DNA methylation levels of EGA-OFF and PFF-ON genes in PFF, SCNT4c, and SCNT4c-TDG. These levels are calculated as the average ratios of the methylated CpG to all CpG. EGA-OFF and PFF-ON genes are shown in two groups: gene promoters (TSSs ±5 kb) with or without CpG island (CGI). *$p < 0.05$, **$p < 0.01$, ***$p < 0.001$; two-tailed Student's t test.

(D) Principal component analysis of expression patterns among different samples.

(E) A schematic illustrating how multiple epigenetic barriers in pig cloning can be overcome. Abnormal H3K9me3, H3K27me3, and DNA methylation are enriched in EGA-OFF and PFF-ON regions during cloned MZT, resulting in the failure of embryonic genome activation and

(legend continued on next page)

the developmental improvement achieved by TDG is mainly related to 5mC demethylation, which is shared by *KDM4A* + GSK126 treatment. Perhaps 5caC accumulation in SCNT4c-KG may not be a reprogramming barrier for MZT, thus further removing 5caC by TDG may not show a further improvement by triple treatment. Future studies also should elucidate two additional questions: (1) 5mC and 5hmC were comparably enriched in SCNT embryos when compared with PFF, while 5fC and 5caC were reduced in SCNT embryos (Figures 6F and S7H). What causes this difference? (2) AICDA and SMUG1 process EGA-specific profiles and fail to be activated in SCNT embryos (Table S3). What is the role of 5mC deamination in nuclear reprogramming?

In summary, our study advances the understanding of epigenetic remodeling in pig SCNT embryos on a genome-wide scale and provides an effective combination strategy to remove multiple barriers during the MZT. Meanwhile, we defined TDG as a novel and species-specific regulator to potentiate the developmental competence of SCNT embryos. Recently, the H3K9me3 methyltransferase inhibitor chaetocin exhibited a beneficial effect in pig cloning (Weng et al., 2020). Therefore, combinational use of chaetocin, GSK126, and 5AZA will require optimization to further improve the cloning efficiency. This work will accelerate the practical use of the SCNT technique for pig model production and contribute to the studies of human disease, xenotransplantation, and molecular breeding in agriculture.

EXPERIMENTAL PROCEDURES

All experimental procedures were approved by the Animal Care Commission of Huazhong Agriculture University, Wuhan, China.

RNA sequencing and data processing

The total RNA of PFF and 30 SCNT embryos were extracted by TRIzol (Invitrogen) or a PicoPure RNA Isolation Kit (Applied Biosystems), respectively. Double-stranded cDNA was then synthesized by a SMARTer Pico PCR cDNA Synthesis Kit (Clontech) and an Advantage 2 PCR Kit (Clontech), and purified by AMPure XP beads (Beckman Coulter). After fragmentation with a Bioruptor Sonication System (Diagenode), cDNA libraries were generated by a VAHTS Universal V6 RNA-seq Library Prep Kit (Vazyme). Paired-end 150-bp sequencing was performed on an Illumina HiSeq X Ten platform.

Illumina Casava (v1.8.2) was used for base calling. Low-quality reads were removed by Trimmomatic (v0.39). The filtered reads were mapped to the pig reference genome (Sscrofa11.1) with STAR (v2.5.3a) to obtain uniquely mapped reads. Transcripts were reconstructed by StringTie (v2.1.2) to define protein-coding novel transcripts by GffCompare (v0.10.2) and CPC2. Complete reference annotation consisted of novel and known transcripts. Gene expression was then quantified to FPKM using Cufflinks (v2.2.1). The expression levels of repetitive elements were assessed by bedtools (v2.29.2), and normalized by VST count. The RNA-seq signal intensities of repetitive elements were quantified and visualized by deeptools (v3.1.3). RepeatMasker annotation was downloaded from the UCSC Genome Browser. Pearson correlation calculation, hierarchical clustering analysis, and PCA were performed using cor, hclust, and princomp functions in R (v3.5.2), respectively. Mapping reads of genes and repeats were visualized using Integrative Genomics Viewer (IGV) software (v2.7.2).

Identification of differentially expressed genes

FC and adjusted p values among samples were analyzed by DESeq2 (v1.22.1), based on the reads count obtained by HTSeq (v0.9.1). DEGs in Figure S1 were extracted using the following criteria: EGA-ON genes in IVO4c, $FC^{IVO4c/IVO2c} > 3$, average FPKM in IVO4c > 5; PFF-OFF genes in IVO4c, $FC^{PFF/IVO4c} > 3$, average FPKM in PFF > 5. Both genes were classified into three groups based on the expression differences among IVO4c, PFF, and SCNT4c. We also defined $FC^{SCNT4c\text{-}KG/SCNT4c} > 2$ and $FC^{SCNT4c\text{-}TDG/SCNT4c} > 2$ as EGA-OFF genes reactivated in SCNT4c-KG/TDG, while $FC^{SCNT4c/SCNT4c\text{-}KG} > 2$ and $FC^{SCNT4c/SCNT4c\text{-}TDG} > 2$ were PFF-ON genes silenced in SCNT4c-KG/TDG. Adjusted p values of all DEGs were < 0.05. GO analysis was performed using clusterProfiler function in R.

Identification of differentially expressed regions

The expression levels of genomic regions among samples were assessed through a sliding window (size 50 kb, step size 20 kb) made by bedtools, and normalized by RPM. Differentially expressed regions (DERs) in Figure 1 were extracted using the following criteria: EGA-ON regions in IVO4c, $FC^{IVO4c/IVO2c} > 5$, average RPM in IVO4c > 5; PFF-OFF regions in IVO4c, $FC^{PFF/IVO4c} > 5$, average RPM in PFF > 5. Both regions were classified into three groups based on the expression differences among IVO4c, PFF, and SCNT4c. We also defined $FC^{SCNT4c\text{-}KG/SCNT4c} > 2$ and $FC^{SCNT4c\text{-}TDG/SCNT4c} > 2$ as EGA-OFF regions reactivated in SCNT4c-KG/TDG, while $FC^{SCNT4c/SCNT4c\text{-}KG} > 2$ and $FC^{SCNT4c/SCNT4c\text{-}TDG} > 2$ were PFF-ON regions silenced in SCNT4c-KG/TDG. Fisher's exact test p values of all DERs were < 0.05.

ULI-NChIP sequencing and data processing

ULI-NChIP was performed as previously described (Brind'Amour et al., 2015). For each immunoprecipitation reaction, 200 PFF or 50 four-cell embryos were added in Nuclear Isolation buffer and MNase Master Mix to digest chromatin for 7 min at 25°C. Chromatin was diluted in Complete Immunoprecipitation buffer, and incubated with 2 μg of antibody-bead complexes (H3K4me3,

somatic cell memory silencing. Combining *KDM4A* overexpression and GSK126 incubation could remove H3K9me3, H3K27me3, and DNA methylation, restore the global transcriptome and repeatome, and provide a double increase for cloned blastocyst formation. Moreover, DNA demethylation and developmental improvement could also be achieved by *TDG* overexpression.
See also Figures S6 and S7; Tables S3 and S4.

ab8580; H3K9me3, ab8898; H3K27me3, 07-449; Dynabeads Protein G, Invitrogen) overnight at 4°C. After washing with Low Salt Wash buffer and High Salt Wash buffer, DNA was eluted in Elution buffer for 2 h at 65°C, and purified by phenol chloroform. Raw ChIP material was then used for library construction by a KAPA HyperPrep Kit (Roche). Paired-end 150-bp sequencing was performed on an Illumina HiSeq X Ten platform.

Illumina Casava (v1.8.2) was used for base calling. Sequenced reads were trimmed for adapter sequence, low-quality, and low-complexity reads, then mapped to Sscrofa11.1 reference genome using Bowtie2 (v2.3.3.1). Multiple mapped reads, PCR duplicates, and reads with a map quality score of less than 30 were removed. The correlation coefficients of replicates, ChIP-seq intensity, and the occupying rates of exonic and repetitive sequences were quantified and visualized by deeptools (v3.1.3). Peak calling was performed by MACS2 (v2.2.5) with the parameters "macs2 callpeak –n Sample –g 2.2e9 -B -p 0.05" relative to input samples. Mapping reads of representative genomic regions were visualized using IGV software.

PBAT sequencing and data processing

A pool of PFF and four-cell SCNT embryos (50 cells) was harvested for PBAT-seq as previously reported (Miura et al., 2012; Smallwood et al., 2014). In brief, cell lysate was bisulfite converted using a MethylCode Bisulfite Conversion Kit (Invitrogen), performing ten-round random priming by Klenow exo- (NEB) and scBS-seq-bio-P5-N9-oligo1 (CTA CAC GAC GCT CTT CCG ATC TNN NNN NNN N). After capturing biotinylated DNA on Dynabeads M-280 Streptavidin (Invitrogen), the second strand was synthesized using scBS-seq-P7-N9-oligo2 (AGA CGT GTG CTC TTC CGA TCT NNN NNN NNN). DNA was then amplified using 2× KAPA HiFi HotStart ReadyMix (Roche) and the index/universal primers (NEB). Paired-end 150-bp sequencing was performed on an Illumina NovaSeq 6000 platform.

Illumina Casava (v1.8.2) was used for base calling. Trimmed reads were then mapped to Sscrofa11.1 reference genome using bismark (v0.22.3). Multiple mapped reads, PCR duplicates, and reads with a map quality score of less than 30 were removed. The DNA methylation levels were quantified and visualized by bismark and deeptools (v3.1.3), respectively.

Statistical analysis

All experiments were repeated at least three times. All middle lines in the box-whisker plots indicate the median, the edges indicate the $25^{th}/75^{th}$ percentiles, and the whiskers indicate the $2.5^{th}/97.5^{th}$ percentiles. Development rates and qPCR results are presented as the mean ± SD. p values were calculated using two-tailed Student's t test with SPSS Statistics 20 software (IBM). Differences are shown with asterisks indicating *$p < 0.05$, **$p < 0.01$, and ***$p < 0.001$.

Data and code availability

The Gene Expression Omnibus accession numbers for the data reported in this paper are GEO: GSE161527 and GSE139512.

SUPPLEMENTAL INFORMATION

Supplemental information can be found online at https://doi.org/10.1016/j.stemcr.2021.09.012.

AUTHOR CONTRIBUTIONS

Conceptualization, Y.-L.M., Z.-X.C., and X.L.; methodology, X.L., T.W., L.C., and J. Zhou; software, Z.-X.C., L.C., J. Zhang, and W.Z.; validation, X.L. and J. Zhou; formal analysis, X.L., T.W., L.C., and J. Zhou; investigation, T.W., Z. Li, G.B., S.Y., D.W., C.D., T.X., H.H., L.Y., Z. Liu, and X.Z.; resources, T.W., Z. Li, G.B., S.Y., D.W., C.D., T.X., H.H., L.Y., Z. Liu, and X.Z.; data curation, L.C. and J. Zhang; writing – original draft, X.L., T.W., and L.C.; writing – review and editing, Y.-L.M., X.L., T.W., and L.C.; visualization, X.L., L.C., J. Zhang, and W.Z.; supervision and project administration, Y.-L.M. and Z.-X.C.; funding acquisition, Y.-L.M.

CONFLICTS OF INTERESTS

The authors declare no competing interests.

ACKNOWLEDGMENTS

We thank Prof. Shaorong Gao (Tongji University, Shanghai, China) for providing ULI-NChIP-seq guidance, Prof. Zhonghua Liu (Northeast Agricultural University, Harbin, China) for providing the RNA-seq data of pig IVO embryos, Prof. Shuai Gao (China Agricultural University, Beijing, China) for performing PBAT-seq, and Dr. Heide Schatten (University of Missouri-Columbia) for improving the language. This work was supported by the National Natural Science Foundation of China (no. 31970822), the National Key R&D Program of China, Stem Cell and Translational Research (2016YFA0100203), the Natural Science Foundation of Hubei Province (2018CFA015), and the Fundamental Research Funds for the Central Universities (2662018PY037).

Received: December 25, 2020
Revised: September 20, 2021
Accepted: September 21, 2021
Published: October 21, 2021

REFERENCES

Brind'Amour, J., Liu, S., Hudson, M., Chen, C., Karimi, M.M., and Lorincz, M.C. (2015). An ultra-low-input native ChIP-seq protocol for genome-wide profiling of rare cell populations. Nat. Commun. *6*, 6033.

Cao, S., Han, J., Wu, J., Li, Q., Liu, S., Zhang, W., Pei, Y., Ruan, X., Liu, Z., Wang, X., et al. (2014a). Specific gene-regulation networks during the pre-implantation development of the pig embryo as revealed by deep sequencing. BMC Genom. *15*, 4.

Cao, Z., Zhou, N., Zhang, Y., Zhang, Y., Wu, R., Li, Y., Zhang, Y., and Li, N. (2014b). Dynamic reprogramming of 5-hydroxymethylcytosine during early porcine embryogenesis. Theriogenology *81*, 496–508.

Cao, Z., Li, Y., Chen, Z., Wang, H., Zhang, M., Zhou, N., Wu, R., Ling, Y., Fang, F., Li, N., et al. (2015). Genome-wide dynamic profiling of histone methylation during nuclear transfer-mediated porcine somatic cell reprogramming. PLoS One *10*, e0144897.

Chung, Y.G., Matoba, S., Liu, Y., Eum, J.H., Lu, F., Jiang, W., Lee, J.E., Sepilian, V., Cha, K.Y., Lee, D.R., et al. (2015). Histone demethylase expression enhances human somatic cell nuclear transfer

efficiency and promotes derivation of pluripotent stem cells. Cell Stem Cell *17*, 758–766.

Cortellino, S., Xu, J., Sannai, M., Moore, R., Caretti, E., Cigliano, A., Le Coz, M., Devarajan, K., Wessels, A., Soprano, D., et al. (2011). Thymine DNA glycosylase is essential for active DNA demethylation by linked deamination-base excision repair. Cell *146*, 67–79.

Dambacher, S., Hahn, M., and Schotta, G. (2010). Epigenetic regulation of development by histone lysine methylation. Heredity (Edinb) *105*, 24–37.

Deng, Q., Ramskold, D., Reinius, B., and Sandberg, R. (2014). Single-cell RNA-seq reveals dynamic, random monoallelic gene expression in mammalian cells. Science *343*, 193–196.

Ding, X., Wang, Y., Zhang, D., Wang, Y., Guo, Z., and Zhang, Y. (2008). Increased pre-implantation development of cloned bovine embryos treated with 5-aza-2′-deoxycytidine and trichostatin A. Theriogenology *70*, 622–630.

Gao, R., Wang, C., Gao, Y., Xiu, W., Chen, J., Kou, X., Zhao, Y., Liao, Y., Bai, D., Qiao, Z., et al. (2018). Inhibition of aberrant DNA re-methylation improves post-implantation development of somatic cell nuclear transfer embryos. Cell Stem Cell *23*, 426–435.

Gao, X., Nowak-Imialek, M., Chen, X., Chen, D., Herrmann, D., Ruan, D., Chen, A.C.H., Eckersley-Maslin, M.A., Ahmad, S., Lee, Y.L., et al. (2019). Establishment of porcine and human expanded potential stem cells. Nat. Cell Biol. *21*, 687–699.

Graf, A., Krebs, S., Zakhartchenko, V., Schwalb, B., Blum, H., and Wolf, E. (2014). Fine mapping of genome activation in bovine embryos by RNA sequencing. Proc. Natl. Acad. Sci. U S A *111*, 4139–4144.

Halstead, M.M., Ma, X., Zhou, C., Schultz, R.M., and Ross, P.J. (2020). Chromatin remodeling in bovine embryos indicates species-specific regulation of genome activation. Nat. Commun. *11*, 4654.

He, X., Tan, C., Li, Z., Zhao, C., Shi, J., Zhou, R., Wang, X., Jiang, G., Cai, G., Liu, D., et al. (2019). Characterization and comparative analyses of transcriptomes of cloned and in vivo fertilized porcine pre-implantation embryos. Biol. Open *8*, bio039917.

Hendrickson, P.G., Dorais, J.A., Grow, E.J., Whiddon, J.L., Lim, J.W., Wike, C.L., Weaver, B.D., Pflueger, C., Emery, B.R., Wilcox, A.L., et al. (2017). Conserved roles of mouse DUX and human DUX4 in activating cleavage-stage genes and MERVL/HERVL retrotransposons. Nat. Genet. *49*, 925–934.

Hormanseder, E., Simeone, A., Allen, G.E., Bradshaw, C.R., Figlmuller, M., Gurdon, J., and Jullien, J. (2017). H3K4 methylation-dependent memory of somatic cell identity inhibits reprogramming and development of nuclear transfer embryos. Cell Stem Cell *21*, 135–143.

Huan, Y.J., Zhu, J., Xie, B.T., Wang, J.Y., Liu, S.C., Zhou, Y., Kong, Q.R., He, H.B., and Liu, Z.H. (2013). Treating cloned embryos, but not donor cells, with 5-aza-2′-deoxycytidine enhances the developmental competence of porcine cloned embryos. J. Reprod. Dev. *59*, 442–449.

Jiang, Z., Sun, J., Dong, H., Luo, O., Zheng, X., Obergfell, C., Tang, Y., Bi, J., O'Neill, R., Ruan, Y., et al. (2014). Transcriptional profiles of bovine in vivo pre-implantation development. BMC Genom. *15*, 756.

Kim, K., Doi, A., Wen, B., Ng, K., Zhao, R., Cahan, P., Kim, J., Aryee, M.J., Ji, H., Ehrlich, L.I., et al. (2010). Epigenetic memory in induced pluripotent stem cells. Nature *467*, 285–290.

Kong, Q., Yang, X., Zhang, H., Liu, S., Zhao, J., Zhang, J., Weng, X., Jin, J., and Liu, Z. (2020). Lineage specification and pluripotency revealed by transcriptome analysis from oocyte to blastocyst in pig. FASEB J. *34*, 691–705.

Liu, H., Kim, J.M., and Aoki, F. (2004). Regulation of histone H3 lysine 9 methylation in oocytes and early pre-implantation embryos. Development *131*, 2269–2280.

Liu, W., Liu, X., Wang, C., Gao, Y., Gao, R., Kou, X., Zhao, Y., Li, J., Wu, Y., Xiu, W., et al. (2016). Identification of key factors conquering developmental arrest of somatic cell cloned embryos by combining embryo biopsy and single-cell sequencing. Cell Discov. *2*, 16010.

Liu, X., Wang, Y., Gao, Y., Su, J., Zhang, J., Xing, X., Zhou, C., Yao, K., An, Q., and Zhang, Y. (2018). H3K9 demethylase KDM4E is an epigenetic regulator for bovine embryonic development and a defective factor for nuclear reprogramming. Development *145*, dev158261.

Maiti, A., and Drohat, A.C. (2011). Thymine DNA glycosylase can rapidly excise 5-formylcytosine and 5-carboxylcytosine: potential implications for active demethylation of CpG sites. J. Biol. Chem. *286*, 35334–35338.

Matoba, S., Liu, Y., Lu, F., Iwabuchi, K.A., Shen, L., Inoue, A., and Zhang, Y. (2014). Embryonic development following somatic cell nuclear transfer impeded by persisting histone methylation. Cell *159*, 884–895.

Miura, F., Enomoto, Y., Dairiki, R., and Ito, T. (2012). Amplification-free whole-genome bisulfite sequencing by post-bisulfite adaptor tagging. Nucl. Acids Res. *40*, e136.

Niu, D., Wei, H.J., Lin, L., George, H., Wang, T., Lee, I.H., Zhao, H.Y., Wang, Y., Kan, Y., Shrock, E., et al. (2017). Inactivation of porcine endogenous retrovirus in pigs using CRISPR-Cas9. Science *357*, 1303–1307.

Ruan, D., Peng, J., Wang, X., Ouyang, Z., Zou, Q., Yang, Y., Chen, F., Ge, W., Wu, H., Liu, Z., et al. (2018). XIST derepression in active X chromosome hinders pig somatic cell nuclear transfer. Stem Cell Reports *10*, 494–508.

Saksouk, N., Simboeck, E., and Dejardin, J. (2015). Constitutive heterochromatin formation and transcription in mammals. Epigenetics Chromatin *8*, 3.

Smallwood, S.A., Lee, H.J., Angermueller, C., Krueger, F., Saadeh, H., Peat, J., Andrews, S.R., Stegle, O., Reik, W., and Kelsey, G. (2014). Single-cell genome-wide bisulfite sequencing for assessing epigenetic heterogeneity. Nat. Methods *11*, 817–820.

Sun, L., Wu, K.L., Zhang, D., Wang, H.Y., Wang, Y., Xu, Z.Y., Huang, X.Y., Chen, Z.J., and Liu, H.Q. (2012). Increased cleavage rate of human nuclear transfer embryos after 5-aza-2′-deoxycytidine treatment. Reprod. Biomed. Online *25*, 425–433.

Tsuji, Y., Kato, Y., and Tsunoda, Y. (2009). The developmental potential of mouse somatic cell nuclear-transferred oocytes treated with trichostatin A and 5-aza-2′-deoxycytidine. Zygote *17*, 109–115.

Vanrobays, E., Thomas, M., and Tatout, C. (2017). Heterochromatin positioning and nuclear architecture. Annu. Plant Rev. Online *46*, 157–190.

Weng, X.G., Cai, M.M., Zhang, Y.T., Liu, Y., Liu, C., and Liu, Z.H. (2020). Improvement in the in vitro development of cloned pig embryos after kdm4a overexpression and an H3K9me3 methyltransferase inhibitor treatment. Theriogenology *146*, 162–170.

Xie, B., Zhang, H., Wei, R., Li, Q., Weng, X., Kong, Q., and Liu, Z. (2016). Histone H3 lysine 27 trimethylation acts as an epigenetic barrier in porcine nuclear reprogramming. Reproduction *151*, 9–16.

Xu, K., Zhou, Y., Mu, Y., Liu, Z., Hou, S., Xiong, Y., Fang, L., Ge, C., Wei, Y., Zhang, X., et al. (2020a). CD163 and pAPN double-knockout pigs are resistant to PRRSV and TGEV and exhibit decreased susceptibility to PDCoV while maintaining normal production performance. eLife *9*, e57132.

Xu, R.M., Li, C., Liu, X.Y., and Gao, S.R. (2020b). Insights into epigenetic patterns in mammalian early embryos. Protein & Cell *12*, 7–28.

Yan, L., Yang, M., Guo, H., Yang, L., Wu, J., Li, R., Liu, P., Lian, Y., Zheng, X., Yan, J., et al. (2013). Single-cell RNA-Seq profiling of human preimplantation embryos and embryonic stem cells. Nat. Struct. Mol. Biol. *20*, 1131–1139.

Yan, S., Tu, Z., Liu, Z., Fan, N., Yang, H., Yang, S., Yang, W., Zhao, Y., Ouyang, Z., Lai, C., et al. (2018). A huntingtin knockin pig model recapitulates features of selective neurodegeneration in Huntington's disease. Cell *173*, 989–1002.

Yang, L., Song, L., Liu, X., Bai, L., and Li, G. (2018). KDM6A and KDM6B play contrasting roles in nuclear transfer embryos revealed by MERVL reporter system. EMBO Rep. *19*, e46240.

Yang, X., Smith, S.L., Tian, X.C., Lewin, H.A., Renard, J.P., and Wakayama, T. (2007). Nuclear reprogramming of cloned embryos and its implications for therapeutic cloning. Nat. Genet. *39*, 295–302.

Yue, Y., Xu, W., Kan, Y., Zhao, H.Y., Zhou, Y., Song, X., Wu, J., Xiong, J., Goswami, D., Yang, M., et al. (2020). Extensive germline genome engineering in pigs. Nat. Biomed. Eng. *5*, 134–143.

Zhang, H., Huang, J., Li, Z., Qin, G., Zhang, N., Hai, T., Hong, Q., Zheng, Q., Zhang, Y., Song, R., et al. (2018). Rescuing ocular development in an anophthalmic pig by blastocyst complementation. EMBO Mol. Med. *10*, e8861.

Zhao, J., Whyte, J., and Prather, R.S. (2010). Effect of epigenetic regulation during swine embryogenesis and on cloning by nuclear transfer. Cell Tissue Res *341*, 13–21.

Zheng, Q., Lin, J., Huang, J., Zhang, H., Zhang, R., Zhang, X., Cao, C., Hambly, C., Qin, G., Yao, J., et al. (2017). Reconstitution of UCP1 using CRISPR/Cas9 in the white adipose tissue of pigs decreases fat deposition and improves thermogenic capacity. Proc. Natl. Acad. Sci. U S A *114*, E9474–E9482.

Zhou, C., Wang, Y., Zhang, J., Su, J., An, Q., Liu, X., Zhang, M., Wang, Y., Liu, J., and Zhang, Y. (2019). H3K27me3 is an epigenetic barrier while KDM6A overexpression improves nuclear reprogramming efficiency. FASEB J. *33*, 4638–4652.

Zhou, C., Zhang, J., Zhang, M., Wang, D., Ma, Y., Wang, Y., Wang, Y., Huang, Y., and Zhang, Y. (2020). Transcriptional memory inherited from donor cells is a developmental defect of bovine cloned embryos. FASEB J. *34*, 1637–1651.

Zhu, P., Guo, H., Ren, Y., Hou, Y., Dong, J., Li, R., Lian, Y., Fan, X., Hu, B., Gao, Y., et al. (2018). Single-cell DNA methylome sequencing of human preimplantation embryos. Nat. Genet. *50*, 12–19.

Stem Cell Reports, Volume *16*

Supplemental Information

TDG is a pig-specific epigenetic regulator with insensitivity to H3K9 and H3K27 demethylation in nuclear transfer embryos

Xin Liu, Lu Chen, Tao Wang, Jilong Zhou, Zhekun Li, Guowei Bu, Jingjing Zhang, Shuyuan Yin, Danya Wu, Chengli Dou, Tian Xu, Hainan He, Wei Zhu, Longtao Yu, Zhiting Liu, Xia Zhang, Zhen-Xia Chen, and Yi-Liang Miao

Supplemental Figures

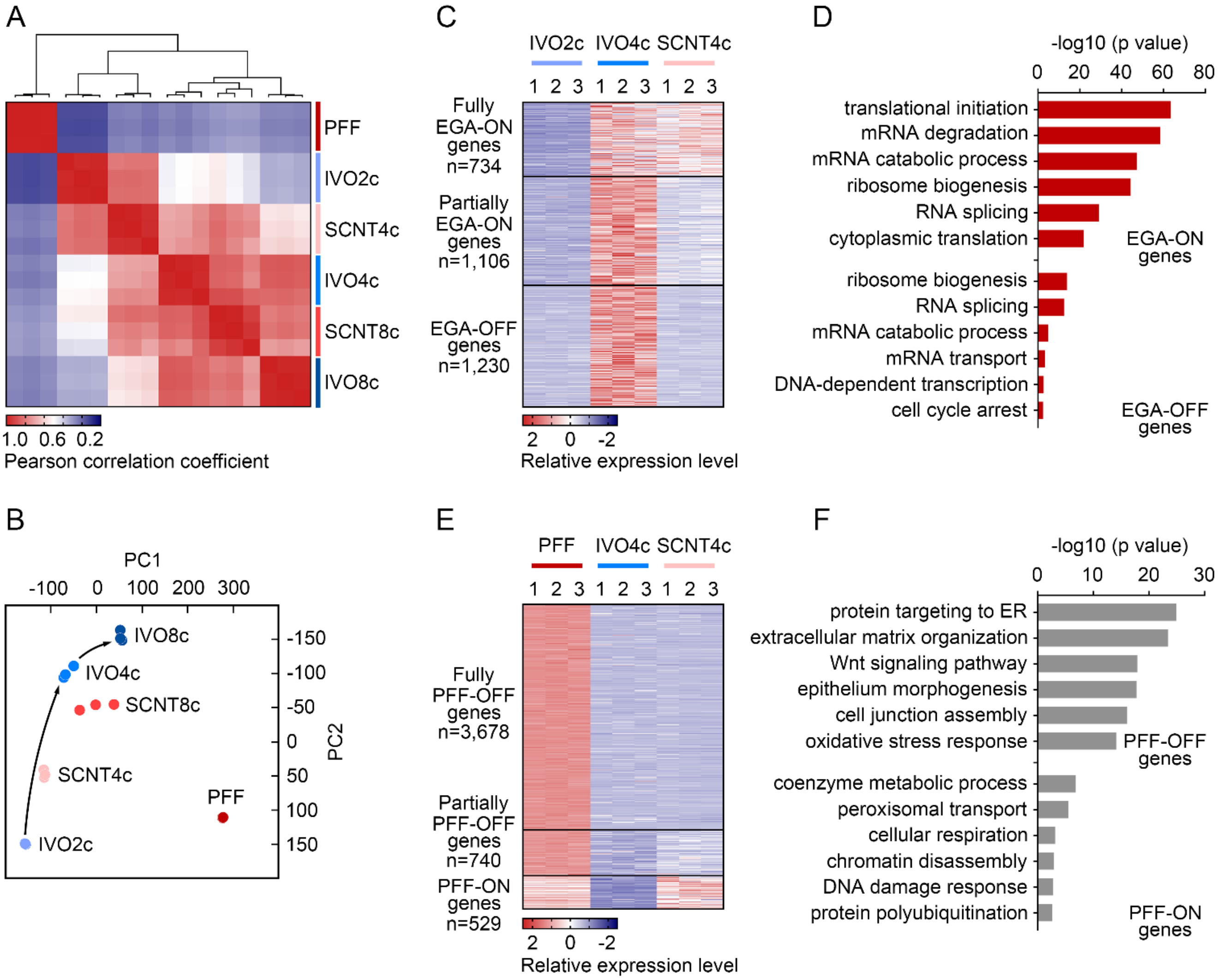

Figure S1. Differentially expressed genes in four-cell SCNT embryos, related to Figure 2.

(A) Heatmap showing the Pearson correlation coefficients in PFF, IVO and SCNT embryos at different stages. Hierarchical clustering is shown in the top panel. 2c, 4c, 8c indicates 2-cell, 4-cell, 8-cell embryos, respectively. RNA-seq data of IVO embryos are from a previous study (Kong et al., 2020).

(B) Principal component analysis of expression patterns among all samples.

(C and E) Heatmap showing the expression levels of differentially expressed genes (DEGs) [fold change (FC) > 3] between IVO2c/PFF and IVO4c, which are classified into six groups by comparing SCNT4c with IVO4c (Fully EGA-ON, FC ≤ 2; Partially EGA-ON, 2 < FC ≤ 5; EGA-OFF, FC > 5) and PFF (Fully PFF-OFF, FC ≥ 5; Partially PFF-OFF, 2 ≤ FC < 5; PFF-ON, FC < 2). Each row represents the normalized FPKM (fragments per kilobase of exon per million mapped fragments) of a transcript and each column represents a replicate.

(D and F) Bar plots showing the gene ontology analysis of EGA-ON/OFF and PFF-ON/OFF genes.

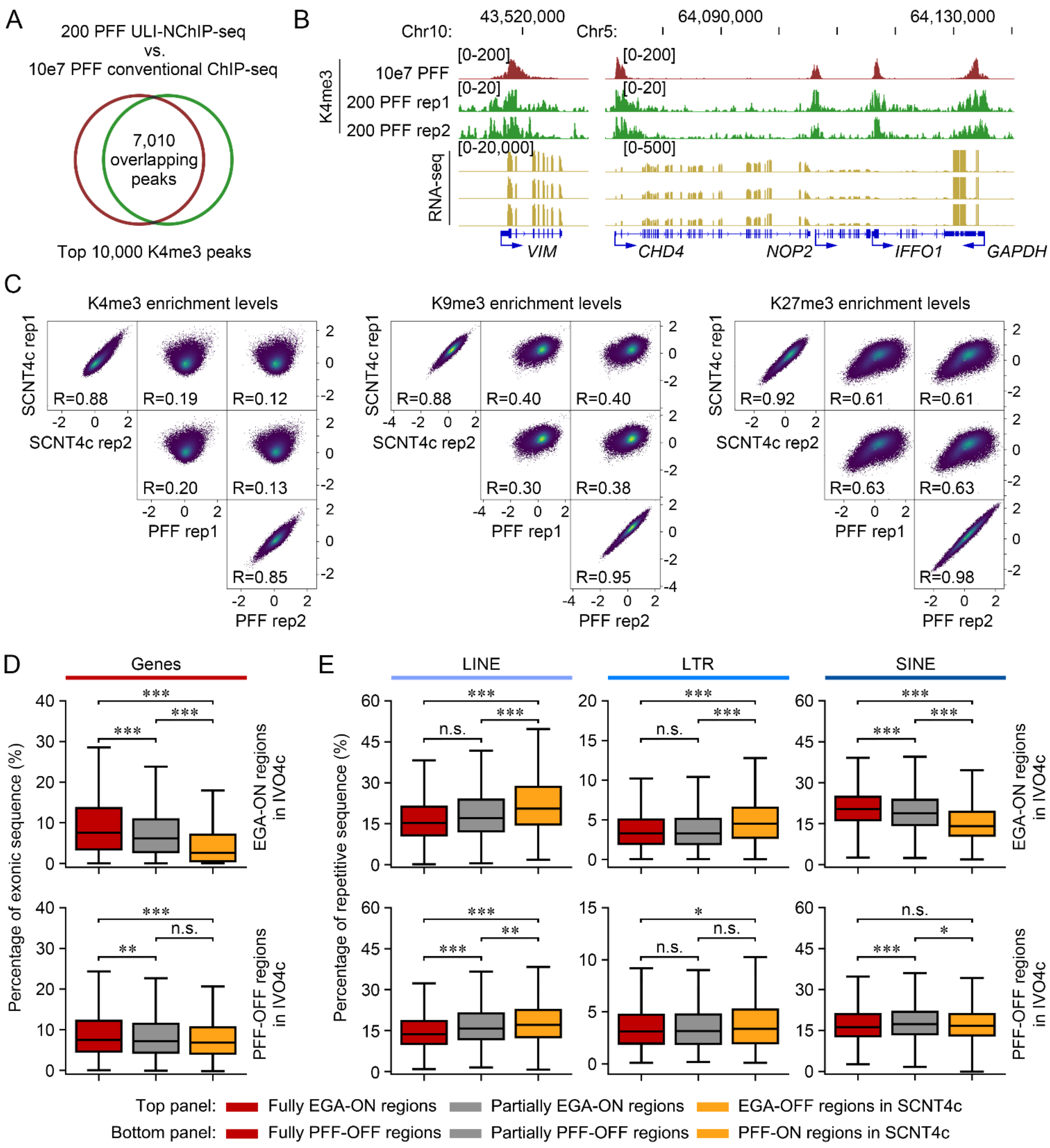

Figure S2. Validation and high reproducibility of ULI-NChIP-seq, related to Figure 1.

(A) The number of overlapping peaks between the top 10,000 H3K4me3 peaks detected from ultra-low-input native chromatin immunoprecipitation sequencing (ULI-NChIP-seq) using 200 PFF and conventional ChIP-seq using 10e7 PFF (Gao et al., 2019).

(B) Genome browser view showing H3K4me3 peaks on the transcription start sites (TSSs) of representative genes from ULI-NChIP-seq and conventional ChIP-seq.

(C) Scatter plots showing the correlations of H3K4me3, H3K9me3, H3K27me3 enrichments among ULI-NChIP-seq samples.

(D) Box plots comparing the average percentage of exonic sequences in EGA-ON/OFF and PFF-ON/OFF

regions in SCNT4c. $^{**}p < 0.01$, $^{***}p < 0.001$; n.s., not significant; two-tailed Student's *t* test.

(E) Box plots comparing the average percentage of repetitive sequences in EGA-ON/OFF and PFF-ON/OFF regions in SCNT4c. $^{*}p < 0.05$, $^{**}p < 0.01$, $^{***}p < 0.001$; n.s., not significant; two-tailed Student's *t* test.

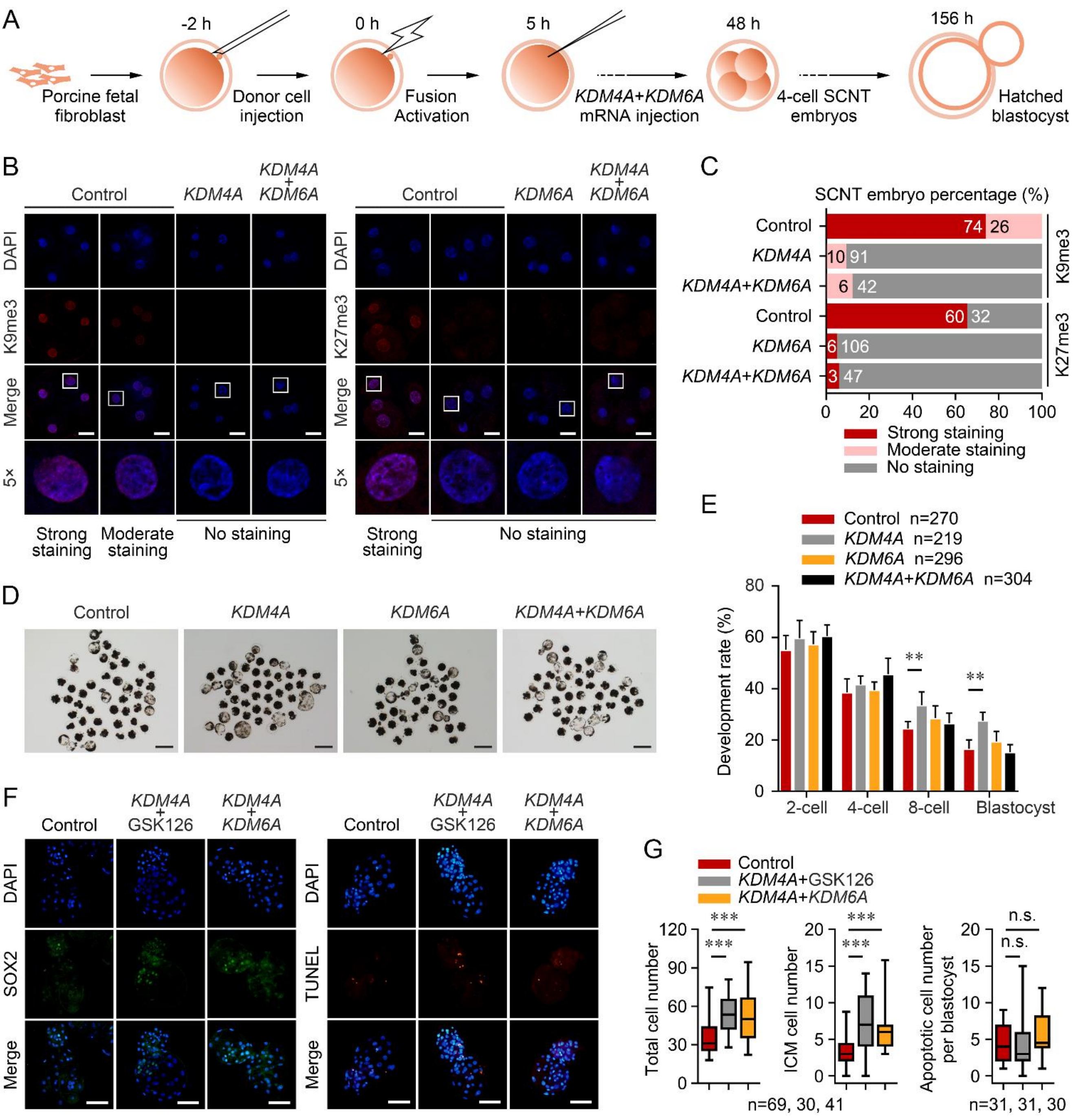

Figure S3. *KDM4A+KDM6A* co-injection cannot improve SCNT embryonic development, related to Figure 3.

(A) Experimental design of the microinjection procedure. SCNT embryos were injected with *KDM4A* and *KDM6A* mRNA at 5 h after activation.

(B) Immunostaining of H3K9me3 and H3K27me3 (red) and DNA (blue) in SCNT4c derived from 1,000 ng/µL *KDM4A*-injected, *KDM6A*-injected, co-injected and non-injected groups. One of the nuclei in SCNT4c is magnified five-fold. Scale bars, 50 µm.

(C) Bar plot showing the percentage of SCNT4c with positive H3K9me3 and H3K27me3 staining and no staining in different groups. Numbers of the total embryos analyzed from 4-5 replicates are shown in the bars.

(D) Representative images of different groups after culturing for 6.5 d *in vitro.* Scale bar, 200 µm.

(E) Bar plot showing the development rates in different groups. Error bars represent the SD. Numbers of the total

embryos analyzed from five replicates are shown in the legend. **$p < 0.01$; two-tailed Student's *t* test.
(F) Labeling of SOX2 (green), apoptotic cells (red) and DNA (blue) in blastocysts derived from *KDM4A*+GSK126 combined-treated, *KDM4A+KDM6A* co-injected and non-treated groups. Scale bars, 100 μm.
(G) Box plots comparing the total cell numbers, inner cell mass (ICM) cell numbers, and apoptotic cell numbers of blastocysts derived from different groups. Numbers of the total blastocysts analyzed from three replicates are shown under the plot. ***$p < 0.001$; n.s., not significant; two-tailed Student's *t* test.

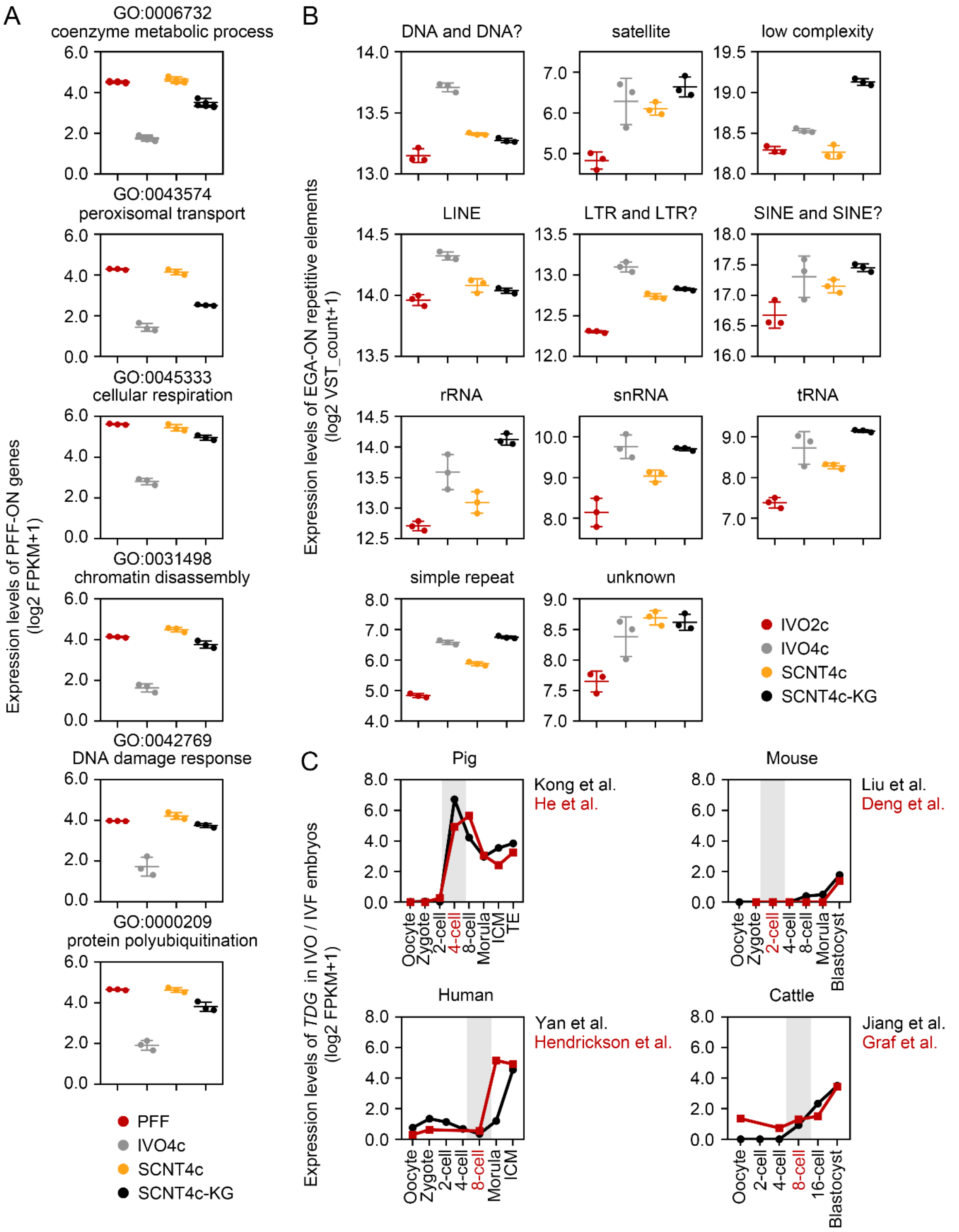

Figure S4. Expression levels of PFF-ON genes, EGA-ON repetitive elements and *TDG*, related to Figures 4-6.

(A) Scatter dot plots comparing the expression levels of candidate PFF-ON genes (Figure S1F) in PFF, IVO4c, SCNT4c and SCNT4c with combined *KDM4A*+GSK126 treatment (SCNT4c-KG). Each plot shows the mean and the SD error bars in three replicates.

(B) Scatter dot plots comparing the expression levels of each EGA-ON repetitive element in IVO2c, IVO4c, SCNT4c and SCNT4c-KG. Each plot shows the mean and the SD error bars in three replicates.

(C) Line plots illustrating the dynamic transcriptional changes of *TDG* in the fertilized embryos of pig (He et al., 2019; Kong et al., 2020), mouse (Deng et al., 2014; Liu et al., 2016), human (Hendrickson et al., 2017; Yan et al., 2013) and cattle (Graf et al., 2014; Jiang et al., 2014). Developmental stage colored in red with gray shade indicates the EGA timing in each species.

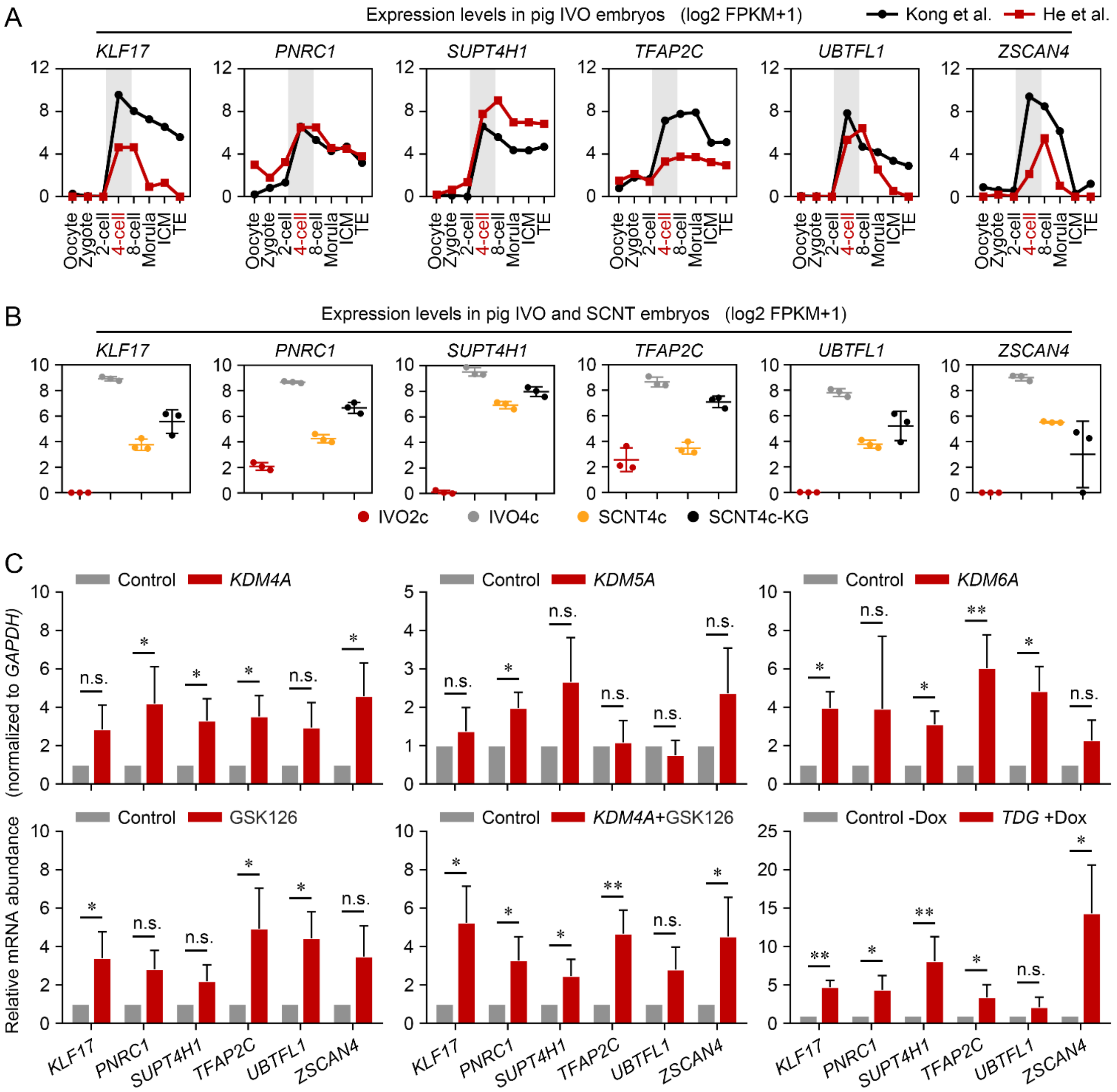

Figure S5. Expression levels of six EGA-OFF genes in four-cell SCNT embryos, related to Figures 4 and 6.

(A) Line plots illustrating the dynamic transcriptional changes of six genes in porcine IVO embryos (He et al., 2019; Kong et al., 2020). Developmental stage colored in red with gray shade indicates the EGA timing in porcine IVO embryos.

(B) Scatter dot plots comparing the expression levels of six genes in IVO2c, IVO4c, SCNT4c and SCNT4c-KG. Each plot shows the mean and the SD error bars in three replicates.

(C) Bar plots showing the mRNA abundances of six EGA-OFF genes in SCNT4c derived from *KDM4A*+GSK126 combined-treated, *TDG* +Dox, *KDM4A*-injected, *KDM5A*-injected, *KDM6A*-injected, GSK126-treated and their corresponding non-treated groups. The results from non-treated groups are set as 1. Error bars represent the SD in 3-4 replicates. *p < 0.05, **p < 0.01; n.s., not significant; two-tailed Student's *t* test.

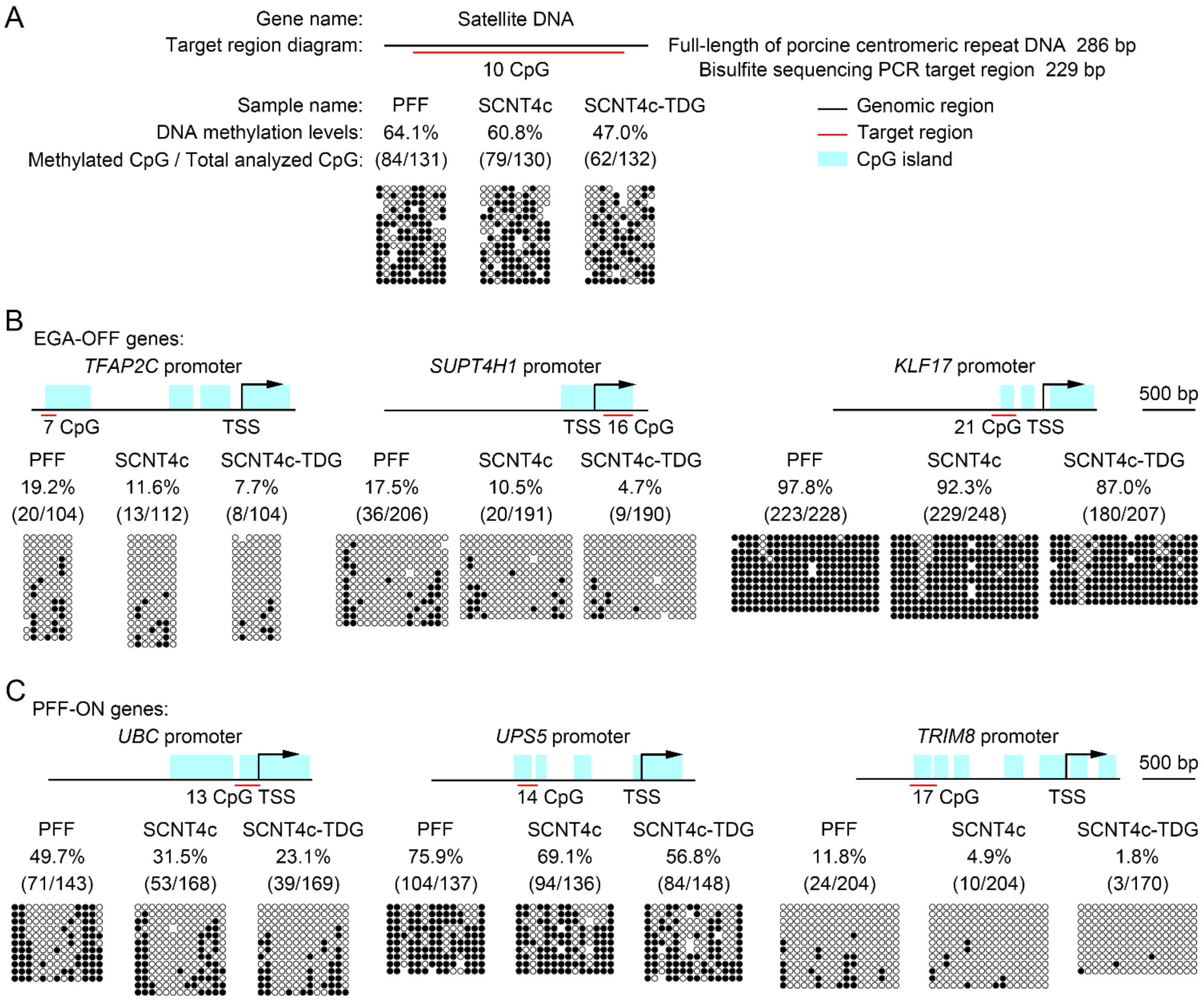

Figure S6. TDG reduces DNA methylation levels in four-cell SCNT embryos, related to Figures 6 and 7. (A-C) DNA methylation statuses of satellite DNA (A), EGA-OFF gene promoters (B) and PFF-ON gene promoters (C) in PFF, SCNT4c and SCNT4c overexpressed with *TDG* (SCNT4c-TDG). DNA methylation levels are determined by bisulfite sequencing PCR (BS-PCR), and are shown as the percentage of methylated CpG relative to the total analyzed CpG. Numbers in parentheses indicate methylated CpG number and total analyzed CpG number, respectively. White and black circles represent unmethylated and methylated CpG, respectively. Each horizontal line represents one individual clone. The diagram in each figure denotes the BS-PCR target regions (red lines) near CpG islands (blue regions) in gene promoters (upstream 2,000 bp and downstream 500 bp from TSSs). CpG number in target region is shown under red lines.

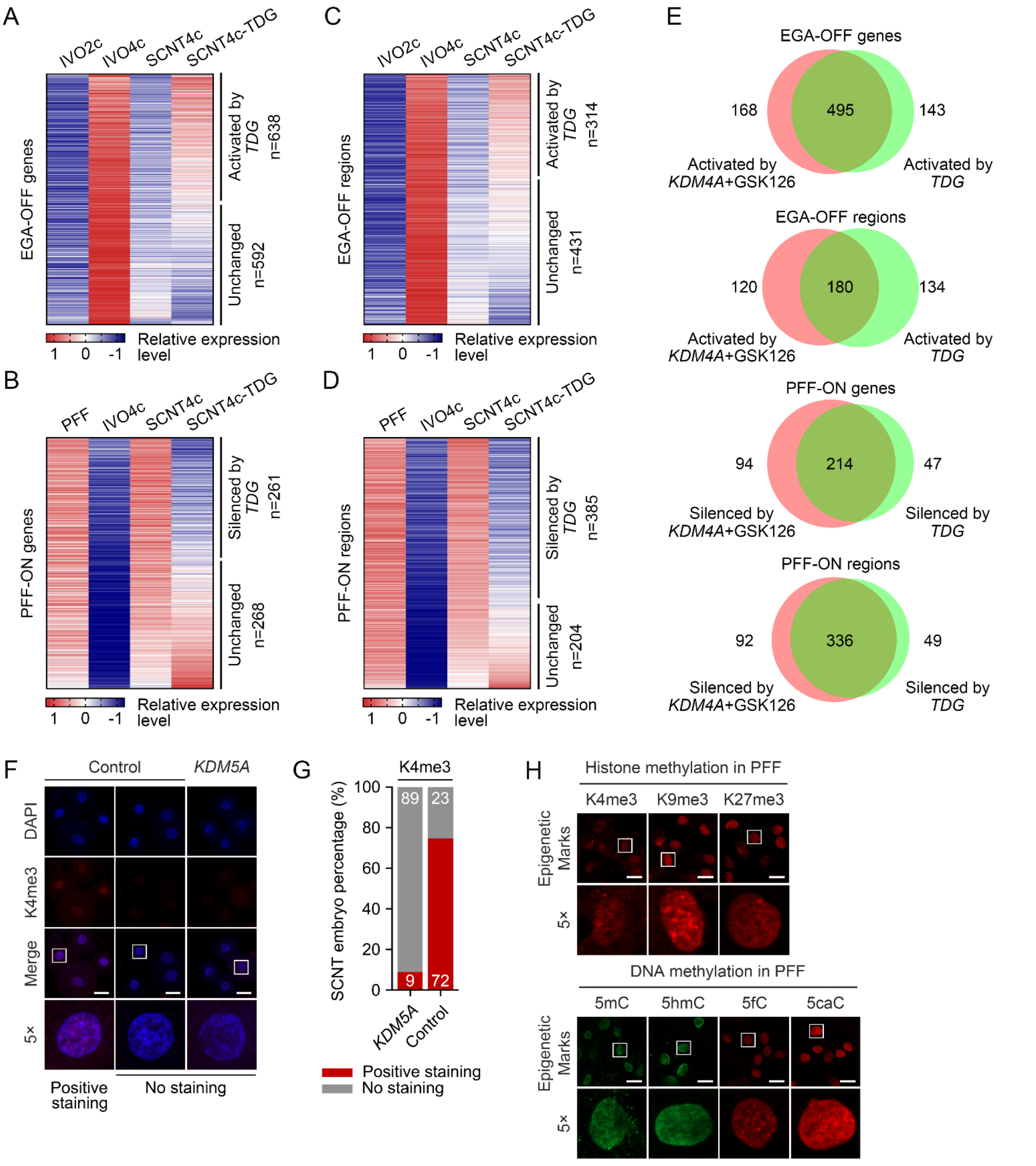

Figure S7. TDG facilitates EGA initiation and somatic cell memory silencing, related to Figures 6 and 7.

(A and B) Heatmap showing the expression levels of EGA-OFF genes (Figure S1C) among IVO2c, IVO4c, SCNT4c, SCNT4c-TDG, and PFF-ON genes (Figure S1E) among PFF, IVO4c, SCNT4c, SCNT4c-TDG. Each row represents the normalized FPKM of a transcript. FC > 2 are EGA-OFF genes reactivated in SCNT4c-TDG (SCNT4c-TDG versus SCNT4c), FC < 0.5 are PFF-ON genes silenced in SCNT4c-TDG.

(C and D) Heatmap showing the expression levels of EGA-OFF regions (Figure 1A) among IVO2c, IVO4c, SCNT4c, SCNT4c-TDG, and PFF-ON regions (Figure 1B) among PFF, IVO4c, SCNT4c, SCNT4c-TDG. Each

row represents the normalized RPM of a region. FC > 2 are EGA-OFF regions reactivated in SCNT4c-TDG (SCNT4c-TDG versus SCNT4c), FC < 0.5 are PFF-ON regions silenced in SCNT4c-TDG.

(E) The overlapping number of restored genes and regions between SCNT4c-KG and SCNT4c-TDG.

(F) Immunostaining of H3K4me3 (red) and DNA (blue) in SCNT4c derived from 1,000 ng/μL *KDM5A*-injected and non-injected groups. One of the nuclei in SCNT4c is magnified five-fold. Scale bars, 50 μm.

(G) Bar plot showing the percentage of SCNT4c derived from different groups with positive H3K4me3 staining and no staining. Numbers of the total embryos analyzed from four replicates are shown in the bars.

(H) Immunostaining of H3K4me3 (red), H3K9me3 (red), H3K27me3 (red), 5mC (green), 5hmC (green), 5fC (red), and 5caC (red) in donor cell PFF. One of the nuclei in PFF is magnified five-fold. Scale bars, 20 μm.

Supplemental Tables

Table S1. RNA-seq and ChIP-seq experiment summary, related to Figure 1.

See supplemental Excel file.

Table S2. *In vitro* development of SCNT embryos with different treatments, related to Figures 3 and 6.

Group	Treatment	Number of replicates	Number of fused one-cell embryos	Cleaved per one-cell (%)	Four-cell per one-cell (%)	Eight-cell per one-cell (%)	Blastocyst per one-cell (%)
Experiment 1	α-amanitin treatment 20 μg/mL	5	276	60.42 ± 5.03 [a]	42.28 ± 4.64 [a]	6.63 ± 1.04 [a]	0.86 ± 1.23 [a]
	DMSO treatment	5	229	54.99 ± 4.79 [a]	40.42 ± 5.46 [a]	27.76 ± 4.38 [b]	20.98 ± 4.18 [b]
	No treatment Control	5	208	58.48 ± 6.36 [a]	43.01 ± 4.03 [a]	26.48 ± 3.61 [b]	18.01 ± 3.47 [b]
Experiment 2	*KDM4A* mRNA injection						
	1,000 ng/uL	3	139	47.66 ± 4.18 [a]	N.A.	N.A.	23.57 ± 4.01 [ab]
	500 ng/uL	3	109	54.01 ± 4.47 [a]	N.A.	N.A.	25.94 ± 3.11 [a]
	100 ng/uL	3	110	53.70 ± 6.42 [a]	N.A.	N.A.	21.66 ± 6.58 [ab]
	20 ng/uL	3	111	55.37 ± 8.06 [a]	N.A.	N.A.	16.30 ± 4.63 [bc]
	Non-injected Control	3	135	48.77 ± 2.93 [a]	N.A.	N.A.	12.44 ± 3.80 [c]
Experiment 3	*KDM5A* mRNA injection						
	1,000 ng/uL	3	142	58.43 ± 4.94 [a]	N.A.	N.A.	12.84 ± 3.94 [a]
	500 ng/uL	3	100	56.32 ± 4.51 [a]	N.A.	N.A.	14.21 ± 2.63 [a]
	100 ng/uL	3	110	55.42 ± 7.01 [a]	N.A.	N.A.	19.58 ± 4.17 [a]
	20 ng/uL	3	104	62.71 ± 2.82 [a]	N.A.	N.A.	18.54 ± 3.44 [a]
	Non-injected Control	3	130	61.12 ± 6.33 [a]	N.A.	N.A.	14.57 ± 4.04 [a]

Experiment 4	*KDM6A* mRNA injection						
	1,000 ng/uL	3	138	56.10 ± 7.96 [ab]	N.A.	N.A.	19.02 ± 5.46 [a]
	500 ng/uL	3	129	54.77 ± 7.77 [ab]	N.A.	N.A.	21.69 ± 2.15 [a]
	100 ng/uL	3	120	64.90 ± 1.14 [a]	N.A.	N.A.	24.62 ± 3.53 [a]
	20 ng/uL	3	150	52.62 ± 2.92 [b]	N.A.	N.A.	23.28 ± 4.63 [a]
	Non-injected Control	3	120	57.08 ± 5.20 [ab]	N.A.	N.A.	17.11 ± 4.69 [a]
Experiment 5	*KDM4A* mRNA injection 1,000 ng/uL	5	219	59.75 ± 6.91 [a]	41.69 ± 3.31 [ab]	33.61 ± 5.14 [a]	27.53 ± 3.30 [a]
	KDM6A mRNA injection 1,000 ng/uL	5	296	57.28 ± 4.95 [a]	39.50 ± 3.11 [ab]	28.51 ± 4.80 [ab]	19.46 ± 3.89 [b]
	KDM4A + *KDM6A* mRNA co-injection	5	304	60.53 ± 4.29 [a]	45.64 ± 6.25 [a]	26.51 ± 3.92 [b]	15.29 ± 2.87 [b]
	Non-injected Control	5	270	55.16 ± 5.59 [a]	38.58 ± 5.28 [b]	24.51 ± 2.66 [b]	16.56 ± 3.51 [b]
Experiment 6	GSK126 treatment						
	0.05 μM for 48 h	3	130	57.10 ± 4.31 [a]	N.A.	N.A.	24.09 ± 2.07 [ab]
	0.1 μM for 48 h	3	110	61.84 ± 5.47 [a]	N.A.	N.A.	28.68 ± 3.67 [b]
	0.5 μM for 48 h	3	135	62.65 ± 7.16 [a]	N.A.	N.A.	25.85 ± 4.90 [b]
	No treatment Control	3	120	54.61 ± 7.41 [a]	N.A.	N.A.	16.82 ± 3.38 [a]
Experiment 7	*KDM4A* mRNA injection 1,000 ng/uL	5	194	59.47 ± 5.41 [a]	44.40 ± 3.55 [ab]	34.43 ± 2.77 [a]	26.78 ± 3.04 [a]
	GSK126 treatment 0.1 μM for 48 h	5	219	61.89 ± 3.63 [a]	42.42 ± 2.34 [ab]	31.60 ± 3.93 [a]	24.71 ± 3.47 [a]
	KDM4A mRNA injection + GSK126 treatment	5	227	63.31 ± 3.78 [a]	47.34 ± 5.21 [a]	39.70 ± 3.70 [b]	33.49 ± 4.22 [b]

	No treatment Control	5	181	58.67 ± 6.48 [a]	41.13 ± 3.09 [b]	26.04 ± 4.76 [c]	15.97 ± 2.07 [c]
Experiment 8	*TDG* mRNA injection						
	1,000 ng/uL	3	156	26.52 ± 6.53 [a]	N.A.	N.A.	0.00 ± 0.00 [a]
	500 ng/uL	3	109	46.82 ± 4.50 [b]	N.A.	N.A.	0.93 ± 1.60 [a]
	100 ng/uL	3	93	50.57 ± 5.42 [bc]	N.A.	N.A.	12.11 ± 4.98 [b]
	20 ng/uL	3	157	54.11 ± 4.34 [bc]	N.A.	N.A.	15.49 ± 3.43 [b]
	Non-injected Control	3	148	58.67 ± 7.83 [c]	N.A.	N.A.	18.34 ± 4.60 [b]
Experiment 9	Dox-inducible *TDG* expression						
	TDG donor cell +Dox	4	167	48.64 ± 3.37 [a]	36.29 ± 2.85 [a]	28.84 ± 5.04 [a]	24.97 ± 2.71 [a]
	TDG donor cell -Dox	4	160	50.76 ± 4.81 [ab]	32.75 ± 3.94 [a]	21.83 ± 1.81 [b]	10.49 ± 4.11 [b]
	Control donor cell +Dox	4	138	54.17 ± 4.02 [ab]	37.34 ± 4.55 [a]	23.01 ± 4.80 [ab]	12.67 ± 5.70 [b]
	Control donor cell -Dox	4	132	56.94 ± 4.73 [b]	39.06 ± 4.77 [a]	25.70 ± 1.76 [ab]	16.20 ± 2.81 [b]
Experiment 10	*KDM4A* + GSK126 + *TDG* (Triple Treatment)	3	128	64.00 ± 5.12 [a]	52.34 ± 5.05 [a]	43.00 ± 3.28 [a]	34.44 ± 3.09 [a]
	KDM4A mRNA injection + GSK126 treatment	3	121	69.75 ± 3.81 [a]	55.66 ± 4.29 [a]	44.69 ± 2.26 [a]	38.65 ± 3.87 [a]
	Non-treated Control	3	115	66.95 ± 6.55 [a]	49.76 ± 3.04 [a]	27.52 ± 2.40 [b]	17.36 ± 3.87 [b]

Data are shown as the mean ± SD. N.A., not available.

[a, b, c] Values with different superscripts differ significantly ($p < 0.05$) in the same group from each other (one-way ANOVA with Duncan test).

Table S3. Expression levels of EGA-ON and PFF-OFF genes among porcine PFF, IVO and SCNT embryos, related to Figures 2, 4, 7.

See supplemental Excel file.

Table S4. Expression levels of EGA-ON and PFF-OFF regions among porcine PFF, IVO and SCNT embryos, related to Figures 1, 4, 7.

See supplemental Excel file.

Table S5. Expression levels of porcine EGA-ON repetitive elements, related to Figure 5.

See supplemental Excel file.

Table S6. Sequences of cDNA amplification primers, quantitative PCR primers, and bisulfite sequencing PCR primers, related to Supplemental Experimental Procedures.

Name	Gene Accession	Experiments	Sense (5′ to 3′)	Antisense (5′ to 3′)
KDM4A	XM_021096835.1	Full-length cDNA amplification	GGAGGGAAATGGCTTCTGAATCTGAAAC	CCGCTGGAAGCACCTACTCCATGATG
		Amplification with T7 promotor primers	GCGAAATTAATACGACTCACTATAGGGAGAGCCACCATGGCTTCTGAATCTGAAAC	GCCGGTTTAGACTACTCCATGATGGCCCGGTACAGCGCAGGCTCGATGTAATC
KDM5A	XM_021092486.1	Full-length cDNA amplification	GCCCCGACCTGAGAGGAAAAG	CCAGCCACCCCAGAACTCAATC
		Amplification with T7 promotor primers	GGCAGCTCGAAATTAATACGACTCACTATAGGGAGACCCAAGCCACCATGGCGGGCATTGGGCCGGGGGGCTACGCGGCGGAGTTTG	GCGGGTTTAAACTAACTGGTCTCTTTAAGATCCTCCACTGGTAGTTTG
KDM6A	XM_021079778.1	Full-length cDNA amplification	GGTGTCGGCGTTGGAGTTGTGA	GTCTGTATGATGTTGCCAGTTTTG
		Amplification with T7 promotor primers	GCGAAATTAATACGACTCACTATAGGGAGAGCCACCATGAAATCCTGCGGAGTGTCGCTCGCTACCGCCGCCGCTGCCGCCGCCGCTTTCGGTGATGAGGAAAAG	GCGGGTTTGAATCAAGATGAGGAGGATGGTAATGGAGGAGCTAATG
TDG	XM_021092634.1	Full-length cDNA amplification	GCCATCGAGTCCAAGAGACTG	GCAGGGCTGAGAAACACTAGTC
		Amplification with T7 promotor primers	GCGAAATTAATACGACTCACTATAGGGAGAGCCACCATGGAAGCAGAGAACGCGAGCAGCTATTC	GCGGGTTTAACTTAAGCACAGTTTCCTTCTTCCTGCTCTTGCGTCCCACGGTGATTGTTAAAG
KLF17	NM_001164010.1	qPCR	AAGATTATGAGGCAGGGTGGAA	CGGAAAACTGTCAAAGACCAAGA
PNRC1	XM_021089951.1	qPCR	CCACCCTCTTTCTCCCTCACT	CTTCTTTCGCCGCTTCTTTGG

SUPT4H1	XM_003131646.4	qPCR	GCTTGTTTGCTGTGTTCGCT	TGTAGGCCACTCCTCGACTT
TFAP2C	NM_001123201.1	qPCR	GATCAGACGGTCATTCGCAAAG	AGAAAACCTCGCTGGGATTCAT
UBTFL1	XM_021102676.1	qPCR	GGCAGTGGGGATGAAGATGAAT	CTTTTGGTGGGAAGTCCTCTGT
ZSCAN4	XM_021097584.1	qPCR	ACAGACGATGGCATGAAGCA	TCGAGGAGTCCAGAGAGGTG
GAPDH	NM_001206359.1	qPCR	TCGGAGTGAACGGATTTG	CCTGGAAGATGGTGATGG
Satellite DNA	Z75640.1	BS-PCR	TTTGTAGAATGTAGTTTTTAGAAG	AAAATCTAAACTACCTCTAACTC
TFAP2C promoter	NM_001123201.1	BS-PCR	AAGGGTAATTAGGATTTGGGGT	CCTAAAATACAACTTAAACAAATTATCT
KLF17 promoter	NM_001164010.1	BS-PCR	TGATGAAAGTTTGATTTTTTTTGTTAGT	CCCTACTCCATCTCAACCTAAAAT
SUPT4H1 promoter	XM_003131646.4	BS-PCR	AGAGGATGGTTTTGGAGATAGTGT	CCTATACTAAAAACTAAAATTACAAAAACT
TRIM8 promoter	XM_001928904.7	BS-PCR	TTTAATTTTAGATAGGGAAAAGGGG	AAAAATAATATCCCTACATCAAAAC
UBC promoter	XM_003483411.4	BS-PCR	TATAGGAAAGTATTTTAGGAGTGAAA	CCTCACCAAATAACAATAACAACAA
USP5 promoter	XM_021092411.1	BS-PCR	GTTTAGGGTATTTGGATGGGAGT	ACCTAAATCACTTCTCACAAACATC

Supplemental Experimental Procedures

Donor cell preparation

Passage 2-5 PFFs were cultured in DMEM medium (Gibco) containing 10% (v/v) fetal bovine serum (Hyclone) for 72 h to induce contact inhibition, and digested by trypsin-EDTA solution to use as donor cells. For cell line establishment, PFFs were transfected with *TDG*-inducible expression vector by using the U023 program on Nucleofector 2b (Lonza), and then selected in culture medium containing 2 μg/mL puromycin for 7 d. The vector construction is based on TLCV2 backbone (Addgene plasmid # 87360).

SCNT

Matured oocytes were collected as a previous report (Liu et al., 2020). Enucleation was then performed with a 20-μm glass pipette by aspirating the first polar body and adjacent cytoplasm of oocytes in manipulation medium containing 7.5 mg/mL cytochalasin B, and a single donor cell was injected into the perivitelline space. Reconstructed embryos were placed in an activation chamber with electrodes 1 mm apart containing activating medium. Two successive direct current pulses (1.2 kV/cm, 30 μs) were applied on an Electro-Cell Manipulator ECM2100 (BTX). After activation (0 h), embryos were cultured in porcine zygote medium-3 (PZM-3) medium (Liu et al., 2020) for 6.5 d under 5% CO_2 in air at 38.5°C, and captured by using Ti-E microscopy (Nikon).

Microinjection and treatment

Full lengths of *KDM4A*, *KDM5A*, *KDM6A* and *TDG* were amplified from pig ovarian cDNA with primers containing T7 promoters by PrimeSTAR GXL DNA Polymerase (Takara) (Table S6). mRNAs were synthesized by mMESSAGE mMACHINE T7 Ultra Kit (Invitrogen) according to the manufacturer's instructions. For microinjection, 5 h-post-activated SCNT embryos were placed in manipulation medium and injected with 20-1,000 ng/μl mRNAs by FemtoJet 4i (Eppendorf), and cultured in PZM-3 medium for 6.5 d. For treatment, reconstructed embryos were cultured in PZM-3 medium containing 0.05-0.5 μM GSK126 (S7061, Selleck) at 0-48 h, 500 ng/mL doxycycline (631311, Clontech) at 40-72 h, or 20 μg/mL α-amanitin (A2236, Sigma-Aldrich) for 6.5 d.

Outgrowth

Outgrowth experiment of pig blastocysts was performed according to a previous study (Zhang et al., 2019). Briefly, SCNT blastocysts cultured for 7 d were seeded on mitotically inactivated mouse embryonic fibroblasts in KO-DMEM-based pig pluripotent stem cell medium. After culturing for 7 d under 5% O_2, 5% CO_2, 90% N_2 at 39°C, the outgrowth colonies were fixed in 4% paraformaldehyde for 15 min, stained with alkaline phosphatase by Fast Red TR/α-Naphthol AS-MX mixture (Sigma-Aldrich) for 15 min, and captured under bright-field microscopy.

Quantitative PCR

Total RNA was isolated from 30 four-cell embryos by RNAprep Pure Micro Kit (TIANGEN). First-strand cDNAs were synthesized by HiScript II Q RT SuperMix Kit plus gDNA wiper (Vazyme), and quantified by ChamQ Universal SYBR qPCR Master Mix (Vazyme) on CFX96 Real-Time PCR Detection System (Bio-Rad). The results were normalized to the internal control gene *GAPDH* (Kuijk et al., 2007). Primer sequences are listed in Table S6. Data are shown as the fold change = $2^{-\Delta\Delta Ct}$ mean ± SD.

Immunofluorescence

For histone methylation staining, samples were fixed in 4% paraformaldehyde for 30 min, permeabilized in 0.5% Triton X-100 for 30 min, blocked in 5% bovine serum albumin for 2 h, and incubated with primary antibodies (H3K4me3, ab8580, Abcam; H3K9me3, ab8898, Abcam; H3K27me3, 07-449, Merck; 1:500) overnight at 4°C. After three washes, embryos were incubated with corresponding secondary antibodies (Abbkine) for 1 h. For

DNA methylation staining, permeabilized samples were denatured with 4 N HCl for 10 min, neutralized with 100 mM Tris-HCl (pH 8.5) for 20 min, and incubated with primary (5mC, 39649; 5hmC, 39999; 5fC, 61228; 5caC, 61230; Active Motif; 1:100) and secondary antibodies as mentioned above. For cell number counting, permeabilized blastocysts were blocked and incubated with SOX2 antibody (sc-365823, Santa Cruz; 1:200). The TUNEL signals of permeabilized blastocysts were detected by using One Step TUNEL Apoptosis Assay Kit (Beyotime). All samples were mounted on glass slides in VECTASHIELD antifade medium (Vector Laboratories) containing 4,6-diamidino-2-phenylindole (DAPI), and captured on an LSM 800 confocal microscope (ZEISS). All steps were performed at room temperature unless stated otherwise.

Bisulfite sequencing PCR

A pool of 30 SCNT embryos and 96-well PFFs was harvested and bisulfite conversion was conducted by using EZ DNA Methylation-Direct Kit (Zymo Research) according to the manufacturer's instructions. Bisulfite sequencing PCR (BS-PCR) was then performed by using ZymoTaq DNA Polymerase (Zymo Research) and the primers designed from MethPrimer (v2.0 and v1.0) based on candidate gene promoter sequences (upstream 2,000 bp and downstream 500 bp from the TSSs) (Table S6). BS-PCR primers for porcine satellite DNA was from a previous study (Kang et al., 2001) (Table S6). We sequenced 10-16 clones from three independent samples. DNA methylation levels were evaluated as the ratio of methylated CpG number to the total CpG number by BiQ Analyzer (v2.02).

Supplemental References

Deng, Q., Ramskold, D., Reinius, B., and Sandberg, R. (2014). Single-cell RNA-seq reveals dynamic, random monoallelic gene expression in mammalian cells. Science *343*, 193-196.

Gao, X., Nowak-Imialek, M., Chen, X., Chen, D., Herrmann, D., Ruan, D., Chen, A.C.H., Eckersley-Maslin, M.A., Ahmad, S., Lee, Y.L., et al. (2019). Establishment of porcine and human expanded potential stem cells. Nat. Cell Biol. *21*, 687-699.

Graf, A., Krebs, S., Zakhartchenko, V., Schwalb, B., Blum, H., and Wolf, E. (2014). Fine mapping of genome activation in bovine embryos by RNA sequencing. Proc. Natl. Acad. Sci. U S A *111*, 4139-4144.

He, X., Tan, C., Li, Z., Zhao, C., Shi, J., Zhou, R., Wang, X., Jiang, G., Cai, G., Liu, D., et al. (2019). Characterization and comparative analyses of transcriptomes of cloned and in vivo fertilized porcine pre-implantation embryos. Biol. Open *8*, bio039917.

Hendrickson, P.G., Dorais, J.A., Grow, E.J., Whiddon, J.L., Lim, J.W., Wike, C.L., Weaver, B.D., Pflueger, C., Emery, B.R., Wilcox, A.L., et al. (2017). Conserved roles of mouse DUX and human DUX4 in activating cleavage-stage genes and MERVL/HERVL retrotransposons. Nat. Genet. *49*, 925-934.

Jiang, Z., Sun, J., Dong, H., Luo, O., Zheng, X., Obergfell, C., Tang, Y., Bi, J., O'Neill, R., Ruan, Y., et al. (2014). Transcriptional profiles of bovine in vivo pre-implantation development. BMC Genomics *15*, 756.

Kang, Y.K., Koo, D.B., Park, J.S., Choi, Y.H., Kim, H.N., Chang, W.K., Lee, K.K., and Han, Y.M. (2001). Typical demethylation events in cloned pig embryos. Clues on species-specific differences in epigenetic reprogramming of a cloned donor genome. J. Biol. Chem. *276*, 39980-39984.

Kong, Q., Yang, X., Zhang, H., Liu, S., Zhao, J., Zhang, J., Weng, X., Jin, J., and Liu, Z. (2020). Lineage specification and pluripotency revealed by transcriptome analysis from oocyte to blastocyst in pig. FASEB J. *34*, 691-705.

Kuijk, E.W., du Puy, L., van Tol, H.T., Haagsman, H.P., Colenbrander, B., and Roelen, B.A. (2007). Validation of reference genes for quantitative RT-PCR studies in porcine oocytes and preimplantation embryos. BMC Dev. Biol. *7*, 58.

Liu, W., Liu, X., Wang, C., Gao, Y., Gao, R., Kou, X., Zhao, Y., Li, J., Wu, Y., Xiu, W., et al. (2016). Identification

of key factors conquering developmental arrest of somatic cell cloned embryos by combining embryo biopsy and single-cell sequencing. Cell Discov. *2*, 16010.

Liu, X., Hao, Y., Li, Z., Zhou, J., Zhu, H., Bu, G., Liu, Z., Hou, X., Zhang, X., and Miao, Y.L. (2020). Maternal Cytokines CXCL12, VEGFA, and WNT5A Promote Porcine Oocyte Maturation via MAPK Activation and Canonical WNT Inhibition. Front. Cell Dev. Biol. *8*, 578.

Yan, L., Yang, M., Guo, H., Yang, L., Wu, J., Li, R., Liu, P., Lian, Y., Zheng, X., Yan, J., et al. (2013). Single-cell RNA-Seq profiling of human preimplantation embryos and embryonic stem cells. Nat. Struct. Mol. Biol. *20*, 1131-1139.

Zhang, X., Xue, B., Li, Y., Wei, R., Yu, Z., Jin, J., Zhang, Y., and Liu, Z. (2019). A novel chemically defined serum- and feeder-free medium for undifferentiated growth of porcine pluripotent stem cells. J. Cell Physiol. *234*, 15380-15394.
